# Cryo-EM structures of human A2ML1 elucidate the protease-inhibitory mechanism of the A2M family

**DOI:** 10.1038/s41467-022-30758-x

**Published:** 2022-05-31

**Authors:** Nadia Sukusu Nielsen, Alessandra Zarantonello, Seandean Lykke Harwood, Kathrine Tejlgård Jensen, Katarzyna Kjøge, Ida B. Thøgersen, Leif Schauser, Jesper Lykkegaard Karlsen, Gregers R. Andersen, Jan J. Enghild

**Affiliations:** 1 Department of Molecular Biology and Genetics, Aarhus University, Aarhus, Denmark; 2 Cordeliers Research Center, Sorbonne University, Paris, France; 3 QIAGEN Aarhus A/S, Aarhus, Denmark

**Keywords:** Cryoelectron microscopy, Proteases, Complement cascade

## Abstract

A2ML1 is a monomeric protease inhibitor belonging to the A2M superfamily of protease inhibitors and complement factors. Here, we investigate the protease-inhibitory mechanism of human A2ML1 and determine the structures of its native and protease-cleaved conformations. The functional inhibitory unit of A2ML1 is a monomer that depends on covalent binding of the protease (mediated by A2ML1’s thioester) to achieve inhibition. In contrast to the A2M tetramer which traps proteases in two internal chambers formed by four subunits, in protease-cleaved monomeric A2ML1 disordered regions surround the trapped protease and may prevent substrate access. In native A2ML1, the bait region is threaded through a hydrophobic channel, suggesting that disruption of this arrangement by bait region cleavage triggers the extensive conformational changes that result in protease inhibition. Structural comparisons with complement C3/C4 suggest that the A2M superfamily of proteins share this mechanism for the triggering of conformational change occurring upon proteolytic activation.

## Abstract (web summary)

A2ML1 is a human protease inhibitor belonging to the A2M protein family. In this study, the authors determine structures of A2ML1 before and after protease inhibition and investigate its mechanism of action.

## Introduction

Human alpha-2 macroglobulin-like 1 (A2ML1) is a monomeric member of the A2M superfamily (A2MF) of protease inhibitors and complement factors. A2ML1 is abundant in the epidermal granular layer of the skin but is also found in the placenta, thymus, and testis^1^. The physiological role of A2ML1 is unknown, although it is suspected to regulate epidermal proteases involved in keratinocyte differentiation and desquamation^2,3^. In addition, it has been implicated in the pathogenesis of paraneoplastic pemphigus^4^ and otitis media^5,6^. A2ML1 and other A2MF proteins share a common domain architecture. They contain eight macroglobulin (MG) domains with ~100 residues organized in two antiparallel β-sheets. The ~120 residue CUB domain (positioned between MG7 and MG8) likewise contains two β-sheets. The thioester (TE) domain is an α-helical domain of ~300 residues inserted within the CUB domain. Finally, the LNK (linker) region and bait region (BR) form a single continuous insert in the MG6 domain. In the complement members of the A2MF, the bait region is replaced by an anaphylatoxin domain separated from the LNK region in the mature complement factor by furin processing. Complement components C3, C4, and C5 contain an additional C-terminal domain that interacts with complement-specific proteases and subunits of the membrane attack complex^7,8^.

A2MF protease inhibitors utilize a unique trapping mechanism that pseudo-inhibits proteases regardless of their catalytic class. Trapped proteases remain catalytically active but are prevented from cleaving protein substrates through steric hindrance imposed by the inhibitor following a proteolytically triggered conformational change^9–11^. The A2MF protease inhibitors exist as functional monomers in amphibians, reptiles, birds, and rodents^12–15^, as dimers in octopus vulgaris^15^ and human pregnancy zone protein (PZP)^16^, and as tetramers such as human A2M^17^. With a few exceptions (e.g., complement C5), A2MF proteins contain an internal thioester formed between the cysteine and a glutamine side chain in a Cys-Gly-Glu-Gln motif. After bait region cleavage or anaphylatoxin release, this thioester is exposed and can react with nucleophilic amine and hydroxyl groups on proteins and carbohydrates. As a result, the nascent activated A2MF protein may become covalently linked to the bait region-cleaving protease as seen in A2MF protease inhibitors or the complement-activating surface in the case of C3 and C4. A2ML1 can react with both amine and hydroxyl groups^18^, similarly to C3 and C4B^19^, as the nascent thioester is attacked by a nearby histidine residue to form an acyl-imidazole intermediate. The acyl-imidazole group has an increased reactivity that allows it to react with hydroxyl groups in addition to amine groups.

Structural studies of complement factors such as C3 and C4^20,21^ provide a detailed understanding of their conformational change following proteolytic cleavage. In contrast, A2MF protease inhibitors are relatively poorly structurally characterized, excepting a low-resolution structure of methylamine-treated A2M that suggests a subunit conformation different from that of complement C3b and C4b^22^ and a recently determined cryo-EM structure of a native tetrameric ovostatin (which strongly resembles A2M) from the frog *X. laevis*^23^. Structures for two bacterial A2MF protease inhibitors, thought to originate from horizontal gene transfer from animals, have been determined but show that these proteins are structurally quite distinct from their animal counterparts^24–26^.

To provide a structural explanation for the unique mechanism of protease inhibition by A2MF proteins, we determined structures of native A2ML1 and three different protease-cleaved forms of A2ML1, and assessed the ability of an A2ML1-bound protease to cleave substrates. Our results demonstrate that the functional inhibitory unit of A2ML1 is a monomer that requires the formation of a covalent bond between a protease and A2ML1 in order to achieve protease inhibition by steric hindrance. Based on our structures, we propose that disordered regions in A2ML1 provide a pivotal contribution to protease sequestration and we suggest a unified mechanism for the conformational change occurring upon proteolytic activation of A2MF proteins.

## Results and discussion

### Protease inhibition by A2ML1 is dependent on covalent protease conjugation

**Fig. 1:**
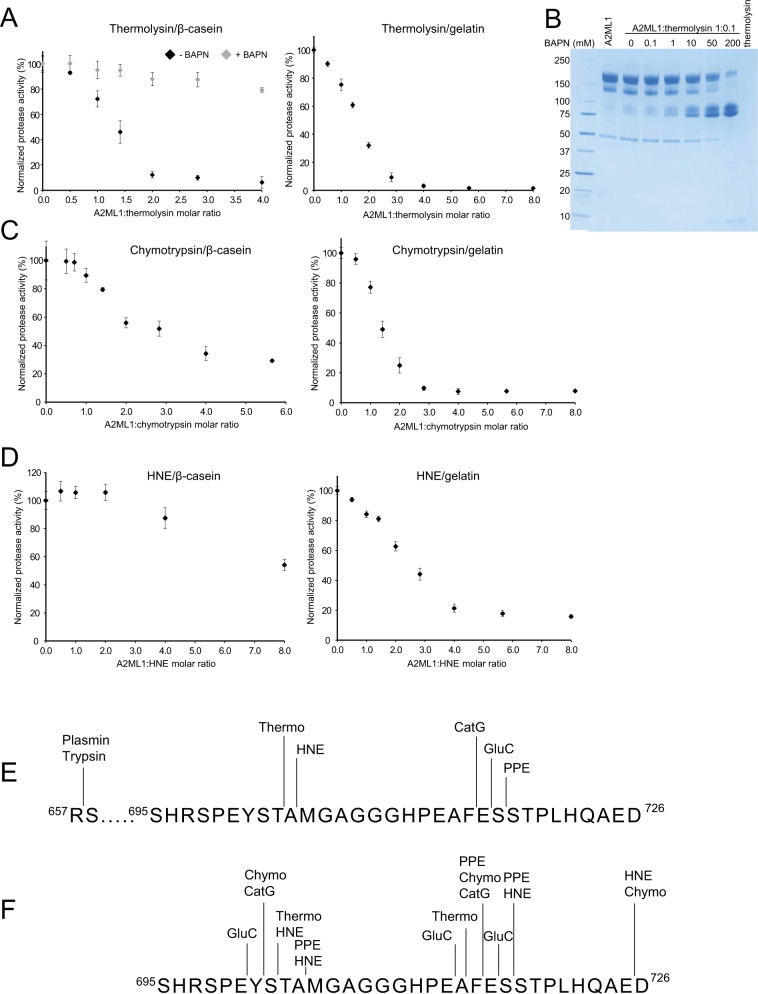
A2ML1 is a monomer and prevents access of large substrates to a conjugated protease. **A** Thermolysin was incubated with the indicated molar ratios of A2ML1 to thermolysin with and without BAPN and the residual protease activity was then measured against β-casein (left) and gelatin without BAPN (right). For both substrates, a 2–3-fold excess of A2ML1 was required for full inhibition of thermolysin, whereas A2ML1 demonstrated little to no inhibition of thermolysin in the presence of BAPN. Data are represented as the mean values +/− standard deviation, *n* = 3 independently prepared samples. **B** SDS-PAGE analysis of A2ML1 cleaved using a 1:0.1 molar ratio of A2ML1 to thermolysin with a titration series of BAPN revealed increased cleavage of intact A2ML1 with increasing BAPN. This supports A2ML1’s dependence on covalent conjugation to the protease in order to achieve inhibition. This image is representative of duplicate experiments. Molecular weight markers are shown on the left-hand side in kDa. **C**, **D** Protease activity assays with chymotrypsin and HNE. In both cases, a higher molar ratio of A2ML1 to the protease is needed to inhibit the protease when casein is used as the substrate compared to the larger gelatin. Source data are provided in the Source data file, see Data availability. Data are represented as the mean values +/− standard deviation, *n* = 3 independently prepared samples. **E**, **F** Cleavage sites for the indicated proteases were determined by Edman sequencing (**E**) or LC-MS/MS (**F**), showing that most tested proteases cleave within the predicted bait region sequence with the exception of the Arg-specific proteases trypsin and plasmin.

The monomeric A2MF protease inhibitor rat A1I3 requires thioester-mediated covalent binding to inhibit proteases^14^, whereas this is not required by tetramers like A2M. A2ML1 is present as a monomer in human skin and remains monomeric after bait region cleavage by proteases (see Supplementary Note 1 and Supplementary Fig. 1 for details). We therefore investigated A2ML1’s dependence on thioester-mediated protease conjugation by determining its inhibitory capacity in the presence or absence of β-aminopropionitrile (BAPN), a nucleophile that prevents conjugation of proteases by competitively reacting with the thioester exposed after proteolytic activation^27^. In the absence of BAPN, thermolysin was almost completely inhibited by a two-fold molar excess of A2ML1 to thermolysin, while BAPN almost completely prevented inhibition even at a four-fold molar excess of A2ML1 (Fig. 1A). In addition, a titration of BAPN with a constant 1:10 thermolysin:A2ML1 molar ratio increased the extent of bait region cleavage to near completion at 200 mM of BAPN (Fig. 1B). This suggests that when protease conjugation is prevented, one protease molecule cleaves multiple A2ML1 molecules without being inhibited. Therefore, protease inhibition by A2ML1 depends on its ability to bind the protease covalently.

The inhibitory mechanism of A2MF protease inhibitors relies on steric hindrance. Trapped proteases are prevented from accessing substrate proteins and larger substrates are sequestered from the protease to a greater extent^14^. Previously, it has been shown that A2ML1 can shield thermolysin and chymotrypsin from accessing a very large substrate, hide powder azure^1^. The ability of A2ML1 to shield proteases from access to substrates of varying sizes was compared by investigating protease inhibition against β-casein (24 kDa), gelatin (average molecular weight of 100 kDa), and A2M (720 kDa) (Fig. 1A, C, D and Supplementary Fig. 2). A2ML1 inhibited 80–95% of the gelatin-cleaving activity of thermolysin, chymotrypsin, and human neutrophil elastase (HNE) at a 4:1 A2ML1:protease molar ratio. With β-casein as the substrate, thermolysin remained ≈95% inhibited at a 4:1 A2ML1:protease molar ratio whereas chymotrypsin and HNE showed greatly decreased inhibition (≈65% and ≈10%). Using A2M as the substrate, a 4:1 A2ML1:protease molar ratio prevented thermolysin or chymotrypsin from cleaving A2M, and this inhibition was not observed when BAPN was added (Supplementary Fig. 2A). Overall, our data indicate that A2ML1’s inhibitory ability depends on substrate size, with less sequestering of the trapped proteases from smaller substrates. Furthermore, the extent of sequestering is protease-dependent, as exemplified by the different extent to which A2ML1 prevents thermolysin and HNE from cleaving β-casein.

### Limited proteolysis of A2ML1 identifies its bait region and a limited repertoire of proteolytic sites

**Fig. 2:**
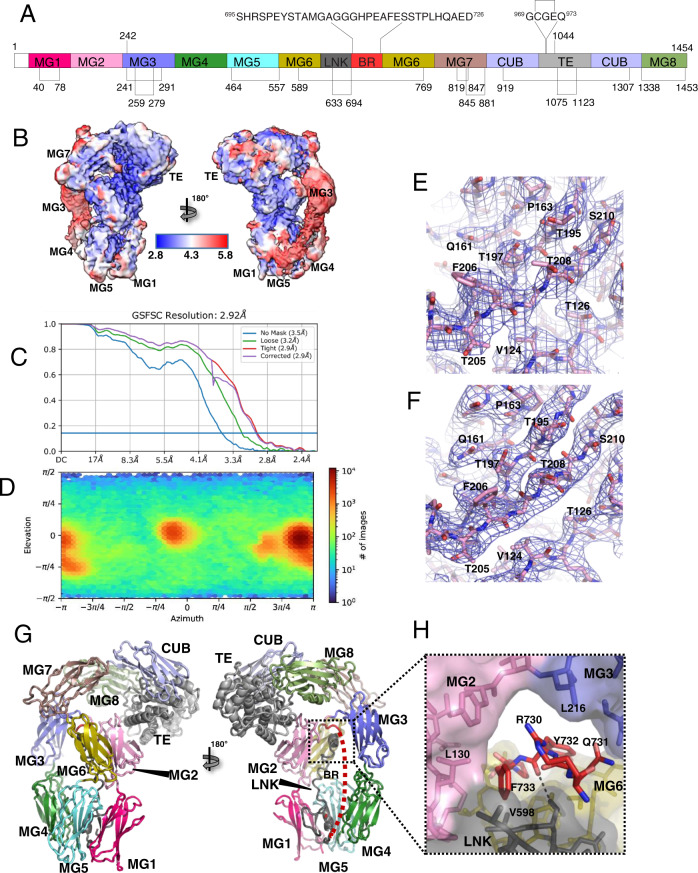
Structures of native A2ML1. **A** Schematic representation of the domains of A2ML1 with disulfides and the internal thioester indicated. Notice that the LNK and BR are inserted in the MG6 domain, whereas the TE domain is inserted in the CUB domain. The MG8 domain is also known as the receptor-binding domain, RBD. **B** Local resolution of the native A2ML1 cryo-EM map in two orientations. **C** Fourier shell correlation indicates a resolution of 2.9 Å. **D** The plot of the particle orientation distribution demonstrates the presence of preferred orientations. **E** Example of EM map quality for native A2ML1 for residues in the MG2 domain. **F** As in panel **E**, but displaying the X-ray map obtained after molecular replacement and density modification at 4.4 Å resolution. **G** Native A2ML1 in a cartoon representation. The domains are colored as in panel **A**. To the right, the dashed red line indicates flexible BR residues not modeled. **H** Close-up on the inter-domain channel accommodating the C-terminal residues of the bait region.

The predicted bait region sequence Ser695-Asp726 (Fig. 2A) in A2ML1 has not been experimentally verified. Thermolysin, cathepsin G (CatG), GluC, and HNE were previously shown to cleave A2ML1 into an N-terminal fragment and a C-terminal fragment where the protease may be conjugated to the cleaved thioester^18^. To assess A2ML1 cleavage by other proteases, we incubated A2ML1 with titration series of chymotrypsin, porcine pancreatic elastase (PPE), and trypsin in the presence and absence of BAPN (Supplementary Fig. 2B). SDS-PAGE analysis showed that PPE cleaves A2ML1 into distinct N- and C-terminal fragments, while the N- and C-terminal fragments were difficult to distinguish for chymotrypsin (Supplementary Fig. 2B), and only one band containing the C-terminal part of A2ML1 was evident after trypsin cleavage. The addition of BAPN significantly increased the cleavage of A2ML1 by chymotrypsin and PPE but not trypsin, suggesting that chymotrypsin and PPE are inhibited by A2ML1, but not trypsin. N-terminal sequencing of the C-terminal fragment and LC-MS/MS analysis were performed to map the A2ML1 cleavage sites for plasmin, trypsin, thermolysin, chymotrypsin, HNE, CatG, GluC, and PPE (Fig. 1E, F). All sequenced cleavage sites were located inside the predicted bait region of A2ML1 except for the plasmin and trypsin sites. These proteases both cleaved at Arg657 outside of the predicted bait region which, along with the lack of increased cleavage of A2ML1 by trypsin in the presence of BAPN (Supplementary Fig. 2B), indicates that cleavage at this site does not result in protease trapping. Importantly, these results indicate that Arg697 is not accessible to cleavage by arginine-specific proteases despite its position in the predicted bait region sequence. In addition to trypsin and plasmin, many physiologically relevant human proteases such as the coagulation factors, FIXa, FXa, and plasma kallikrein are arginine-specific. This implies that a more restricted range of proteases are inhibited by A2ML1 compared to other A2MF protease inhibitors such as A2M.

### Structure determination reveals the native A2ML1 conformation with threading of the bait region through a hydrophobic channel

Structures of native and protease-cleaved A2ML1 were determined to obtain starting point and endpoint of the conformational transition triggered by bait region cleavage and provide a structural foundation for understanding A2ML1’s protease-inhibitory mechanism. Structures of native A2ML1 were determined by X-ray crystallography and cryo-EM at resolutions of 4.4 and 3.1 Å, respectively (Fig. 2B–F, Supplementary Tables 1 and 2, Supplementary Fig. 3). An initial crystallographic map was obtained by molecular replacement with a map excised from an early stage cryo-EM map with a resolution of 3.8 Å. Density modification with 70% solvent and two-fold averaging resulted in an outstanding electron density for the X-ray structure in many regions despite the resolution (Fig. 2F). Overall, the cryo-EM and X-ray structures are very similar with an overall root mean square deviation (RMSD) of 2.0 Å for the entire molecule. Multiple regions in the α-helical TE domain appeared to have better electron density in the X-ray structure than in the EM map, and the Gln1175-Glu1188 loop could only be modeled in this map. These differences in loop flexibility are likely to be an effect of crystal packing interactions involving the TE domain.

Native A2ML1 is organized in two structural units connected by the MG7 domain. The N-terminal residues Glu19-Phe787 constitute the MG-ring formed by MG domains 1–6, the associated LNK region (Fig. 2A, G), and the bait region. The C-terminal domains CUB, TE, and MG8 encompassing residues His904-Glu1454 form a compact structure with extensive CUB-TE and TE-MG8 interactions, which we call the CTM8 body. While the CTM8 body shows minor overall differences between the X-ray and the EM structures, the orientations of the MG3 and MG4 domains within the MG-ring differ significantly due to crystal packing interactions (Supplementary Fig. 4A). In both structures, the map quality for the MG3 and MG4 domains is low compared to the remaining parts of the structure (Fig. 2B and Supplementary Fig. 4B), and the temperature factors are elevated, and map-model correlations lower compared to other parts of the structure (Supplementary Fig. 3B). Residues 634–668 in A2ML1’s LNK region could not be modeled, suggesting that this region adopts multiple conformations.

There are now medium/high-resolution structures for seven A2MF proteins in their native conformation: A2ML1, C3, C4, C5, TEP1r/s from the mosquito *Anopheles gambiae*, a monomeric protease inhibitor from *Salmonella enterica*, and a recently described tetrameric ovostatin from the frog *Xenopus laevis*^21,23,24,28–30^. The packing of the CUB, TE, and MG8 domains within the CTM8 is conserved in all these structures (Supplementary Fig. 4C). This domain arrangement protects the internal thioester located at the MG8-TE domain interface from spontaneous hydrolysis, although it can react with small nucleophilic amines such as methylamine. Both the EM and the X-ray maps for native A2ML1 demonstrate an intact Cys970-Gln973 thioester that is shielded from bulk water through its location in a hydrophobic pocket formed primarily by aromatic sidechains from the MG8 domain and a neighboring loop in the TE domain. The conformation of the MG-ring in A2ML1 is closer to that observed in C3 than to the conformation in TEP1r/s (Supplementary Fig. 4D).

Structures of complement A2MF members C3, C4, and C5^21,29–31^ revealed that an extended stretch of residues (called the Nt-α′ region) located between the ANA domain and the C-terminal half of the MG6 domain passes through a channel formed by the LNK region, the MG2 and MG3 domains, and the MG2-MG3 linker. Upon cleavage, the Nt-α′ region retracts from this channel and relocates to a position between the MG6 and MG7 domains. In A2ML1, the equivalent of Nt-α′ corresponds to the residues C-terminal of the scissile bond in the bait region and for this reason we refer to it as the BR-C region. In the structures of TEP1r/s, the corresponding region is not modeled, suggesting a higher mobility or that it is disordered. In both the EM and the X-ray structures of native A2ML1, it is not possible to model residues His696-Asp726, suggesting substantial flexibility of the intact bait region in accordance with its accessibility toward proteases (Fig. 1E, F). However, in both structures, it is evident that the strictly conserved residues Arg730-Pro734 pass through the same narrow opening that houses the Nt-α′ in complement A2MF proteins. In particular, Tyr732 and Phe733 in the BR-C region form a hydrophobic plug inserted between the highly conserved Leu130, Leu216, and Val598 (Fig. 2H and Supplementary Fig. 4E). We note that Arg730 and Phe733 are strictly conserved in all human A2MF proteins (Supplementary Fig. 4F). Furthermore, the conservation of the surrounding residues suggests that the BR-C regions of A2MF proteins for which native structures have not been determined, e.g., A2M, CD109, CPAMD8, OVOS2, and PZP, passes through a similar narrow opening, equivalent to what has been seen in complement A2MF proteins and now in A2ML1. This hypothesis is supported by the very recent finding that the corresponding BR-C residues in the native A2Moo subunit threads through a similar channel^23^.

In summary, two orthogonal techniques provided consistent atomic structures of native A2ML1. Our structures confirmed that within the CTM8 body, the domains interact in a very similar manner as in prior structures of native A2MF proteins. These structures also provide insight into dynamic properties, as the MG3 and MG4 domains appear to be somewhat mobile in native A2ML1.

### The structure of bait region-cleaved A2ML1 is independent of protease conjugation

**Fig. 3:**
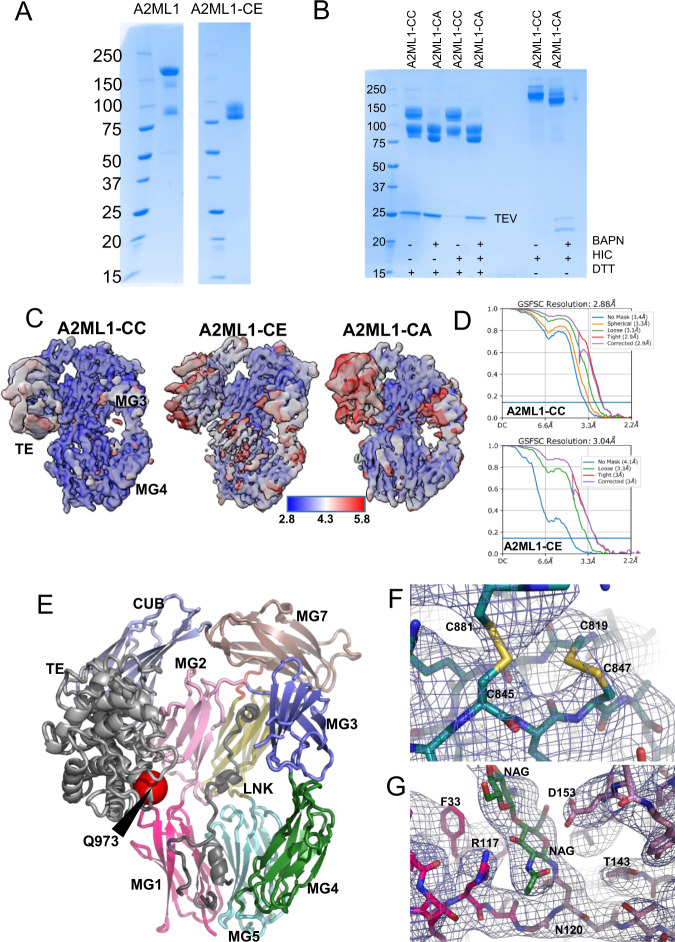
The structures of TEV-P-cleaved A2ML1. **A** SDS-PAGE analysis of A2ML1 and empty trap TEV-P cleaved A2ML1-CE and **B** A2ML1-CC and -CA samples (to the right, the samples are shown after hydrophobic interaction chromatography (HIC) which showed co-elution of TEV-P with A2ML1 even without covalent protease trapping in the A2ML1-CA sample). The A2ML1-CE sample and post-HIC A2ML1-CC and -CA samples that are shown here were used for preparation of grids for cryo-EM; these samples were prepared once. Molecular weight markers on shown on the left-hand side of each gel in kDa. **C** Local resolution maps for the three different monomeric TEV-P cleaved A2ML1. Notice the significantly lower resolution for the TE and MG3 domains compared to the rest of the molecule. **D** Fourier shell correlation plots suggest resolution of 2.9 Å and 3.0 Å for A2ML1-CC and A2ML1-CE, respectively. **E** TEV-P conjugated A2ML1-CC in a cartoon representation with the domains colored as in Fig. 2A. The orientation is similar to that in panel C. The location of thioester glutamine is indicated as a red sphere marked Q973. For comparison, native A2ML1 is presented with the same orientation of the MG-ring in the right part of Fig. 2G. **F** EM map of the MG7 domain in A2ML1-CC confirming two disulfides bridges formed by four cysteines that are located close to each other. **G** EM map for two NAG residues linked to Asn120 in the linker connecting the MG1 and MG2 domains in A2ML1-CC.

In native pore limit electrophoresis, the migration of A2ML1 was slowed after bait region cleavage or methylamine-induced thioester aminolysis (Supplementary Fig. 1D). To elucidate this apparent conformational change, we determined multiple structures of protease-cleaved A2ML1 using a modified A2ML1t protein where a tobacco etch virus protease (TEV-P) cleavage site replaced BR residues 700–706. Structure determination was performed with three distinct preparations of cleaved A2ML1t. First, A2ML1t was cleaved by TEV-P covalently immobilized on a resin, producing bait region-cleaved A2ML1t without conjugated TEV-P (Fig. 3A) which we refer to as A2ML1-CE (CLEAVED, EMPTY). Second, A2ML1t was cleaved by soluble TEV-P without BAPN and covalent A2ML1t-protease complexes were purified by hydrophobic interaction chromatography (Fig. 3B), producing A2ML1-CC (CLEAVED, CONJUGATED TEV-P). Third, A2ML1t was cleaved by soluble TEV-P in the presence of BAPN; unexpectedly, some TEV-P remained non-covalently associated with the cleaved A2ML1t during hydrophobic interaction chromatography (Fig. 3B), and accordingly this sample was referred to as A2ML1-CA (CLEAVED, ASSOCIATED TEV-P). The non-covalent TEV-P association was further investigated, and we concluded that the interaction is due to non-specific protein binding by cleaved A2ML1 not related to its inhibitory mechanism (Supplementary Note 2, Supplementary Fig. 5), similar to the exosite binding reported for A2M^32^. Structures were determined for each of the three types of cleaved A2ML1 monomers by cryo-EM (Supplementary Figs. 6A, 7A and Supplementary Table 1), and these all featured very similar overall conformations. However, a comparison of the local resolution in the three EM maps and map-model correlations (Fig. 3C and Supplementary Figs. 6B, 7B) indicated that A2ML1-CA adopts a wider spectrum of conformations than A2ML1-CC and A2ML1-CE. For this reason, we focus on the structure of A2ML1-CC determined at 2.9 Å resolution and use the structure of A2ML1-CE for comparison (Fig. 3D, E). For all three cleaved A2ML1 samples, ~1/6 of the observed particles picked from EM micrographs were dimers. A structure of the dimer observed in the A2ML1-CA sample was determined to 3.2 Å (Supplementary Figs. 8 and 9). Within the dimer, the subunits are related by an almost perfect two-fold rotation axis perpendicular to the plane of the dimer (Supplementary Fig. 9A, C and D), but the physiological relevance of these dimers is uncertain since dimers of TEV-cleaved A2ML1 were not observed in solution and the dimer interface is not conserved in mammalian A2ML1 sequences (Supplementary Fig. 9E). This dimer is also not related to the dimers within the tetramer of methylamine-treated A2M^22^ or the native A2Moo tetramer^23^ (Supplementary Note 3).

In cleaved A2ML1, the extensive network of domain–domain interactions present within the CTM8 unit of native A2ML1 has collapsed. Instead, the CUB and TE domains are located next to the MG2 domain in the MG-ring (Fig. 3E), similar to the methylamine-treated A2M subunit^22^. The thioester is no longer shielded by the MG8 domain and is now at an exposed position opposite to the MG3 and MG4 domains (Fig. 3E). As described below, we did not observe density in the map that could be attributed to the conjugated TEV-P molecule in A2ML1-CC. Likewise, we were not able to locate the MG8 domain in any of the TEV-P-cleaved A2ML1 structures, implying that it is flexibly attached to the CUB domain in cleaved A2ML1 (see below). As in native A2ML1, it was not possible to model LNK residues Pro634-Pro668 and BR residues His696-Glu725 in cleaved A2ML1. The structures of native and TEV-P-cleaved A2ML1 were used to validate the predicted post-translational disulfide bond formation, for example Cys819-Cys847 and Cys845-Cys881 (Fig. 3F), and MS-identified N-glycans such as that of Asn120 (Fig. 3G). These results are elaborated in Supplementary Note 4 and Supplementary Table 3. In summary, our cryo-EM structures of cleaved A2ML1 demonstrated that bait region cleavage leads to a major conformational change that exposes the thioester, positions the TE domain next to the MG2 domain, and causes the MG8 domain to become unlocked from the remaining A2ML1. Furthermore, protease conjugation does not appear to significantly influence the conformation of cleaved A2ML1.

### A hydrophobic domain interface supports the conformation of cleaved A2ML1, distinguishing it from that of activated complement factors

**Fig. 4:**
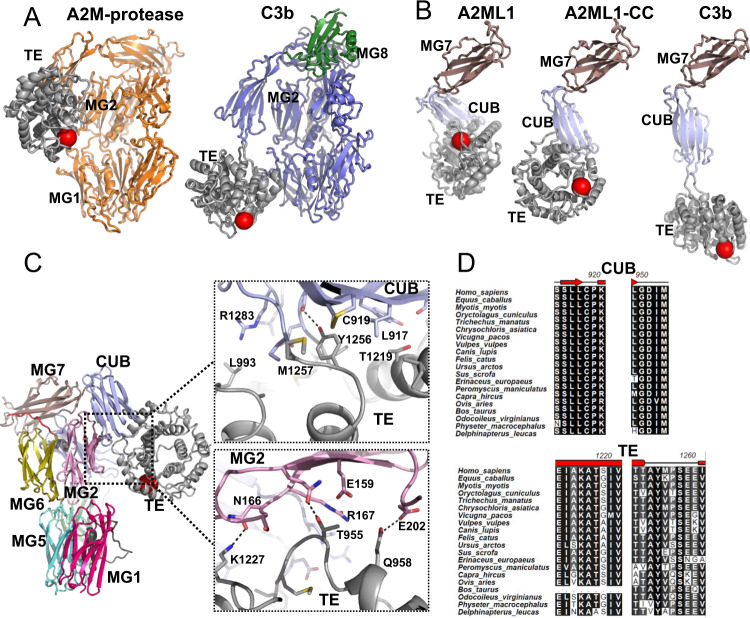
In cleaved A2ML1, the thioester and CUB domains associate through a conserved hydrophobic interface. **A** Conformation of the monomer from the A2M-MA tetramer and C3b for comparison with A2ML1 in Fig. 3E with the thioester glutamines indicated by the red spheres. In both A2ML1 and A2M, the TE and MG2 domains interact, whereas in C3b, the TE domain is instead adjacent to the MG1 domain. Notice the green MG8 domain in C3b that is stably associated with the remaining parts of C3b. A unique C-terminal domain in C3b, C345C, is not shown. **B** Comparison of the conformation of the MG7-CUB-TE domains in A2ML1, A2ML1-CC, and C3b. All structures were superimposed through their MG7 domains to emphasize the movement of the CUB and TE domains upon proteolytic activation of A2ML1 and C3. **C** Expanded views of the interactions formed by the TE domain with the CUB and MG2 domains, top, and bottom, respectively. Putative polar interactions are indicated by dotted lines. **D** Alignment of regions of the CUB and TE domains in mammalian A2ML1 supports that the interface is very highly conserved. The full alignment is presented in Supplementary Fig. 11.

Despite the tetrameric nature of A2M, the monomeric subunit of A2M-MA and A2ML1-CC are very similar, as the two structures superimpose with an RMSD of 3.4 Å over 1076 C_α_ positions (compare Figs. 3E and 4A). This similarity is further emphasized by the flexibility of the MG8 domain observed in both structures. In contrast, a major difference exists between structures of activated A2MF protease inhibitors and complement factors: the TE domain is situated adjacent to the MG2 domain in cleaved A2ML1 and A2M-MA, whereas it is located next to the MG1 domain in C3b and C4b^20,33^ (Fig. 4A). In contrast, the CUB domain is located in a similar location in cleaved A2ML1, A2M-MA, C3b, and C4b. Comparison of the structures for A2ML1, C3, and C4 before and after their proteolytic activation reveals that in all three proteins, the CUB domain rotates by 33–44° while remaining mostly in place, resulting in a center of mass translation of 10–15 Å (Fig. 4B). The differences in overall conformation between cleaved A2ML1 and complement C3b/C4b are therefore mainly due to differences in the rotation that the TE domain experiences relative to the CUB domain upon proteolytic activation. In A2ML1, the TE domain rotates by 69° with a center of mass translation of 10 Å, whereas in C3b, values of 137° and 48 Å are observed (Fig. 4B).

Cleaved A2ML1’s MG2-TE domain interface involves only a few polar contacts, suggesting that a more comprehensive interaction between the TE domain and the CUB domain might determine the position of the TE domain. PISA analysis^34^ of the CUB-TE domain interface revealed a buried surface area of 1380 Å^2^ and a relatively hydrophobic interface with a PISA Δ^i^G *P*-value of 0.18. The interface is organized around a hydrophobic core formed by Tyr1251 and Tyr1256 in the TE domain and Ile952, Tyr1309, Leu917, and the Cys916-Cys1307 from the CUB domain (Fig. 4C). In native A2ML1, Tyr1256 is solvent exposed and the burying of this residue in the hydrophobic interface is probably important for locking the TE domain to the CUB domain in the cleaved state. In agreement with this, Tyr1256 is strictly conserved in A2ML1 (Fig. 4D). Overall, our comparative analysis of the A2MF structures indicates that the protease-induced conformational change involves similar rotations of the CUB domain. In A2ML1, a conserved hydrophobic domain interface anchors the TE domain firmly to the CUB domain.

### The MG8 domain is highly flexible in cleaved and MA-treated A2ML1

**Fig. 5:**
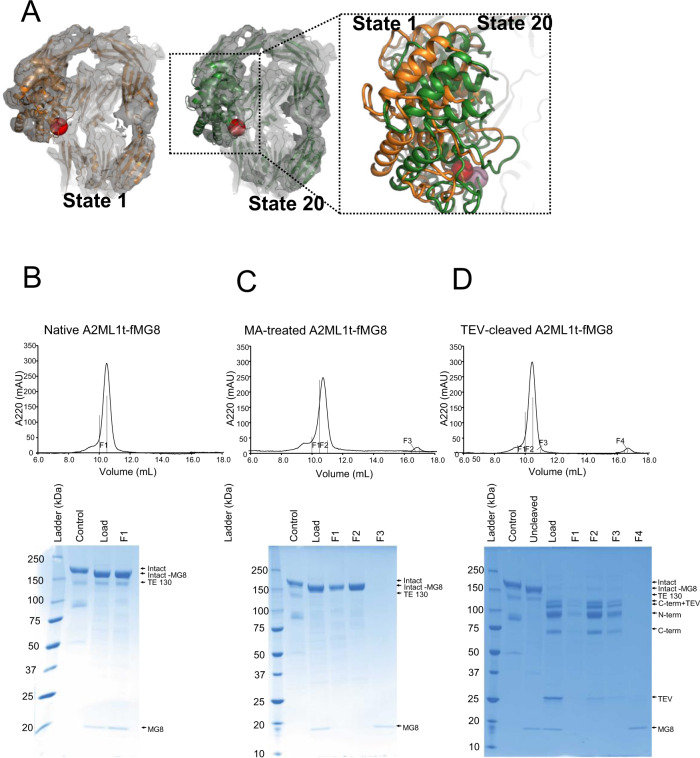
The MG8 domain is flexibly attached to the rest of cleaved A2ML1. **A** The two most extreme states observed in a 3D variability analysis of the A2ML1-CC with map and models superimposed. Except for the TE and CUB domains, only limited differences between the two states are observed. In the magnified view, the extreme positions of the TE domain are compared. **B**–**D** SDS-PAGE analyses of selected fractions from the SEC runs with native, methylamine-activated, and TEV-P cleaved A2ML1t-fMG8. Native A2ML1 was used as a control during SDS-PAGE analysis. The identities of the specific bands on the SDS-PAGE gels were confirmed by LC-MS/MS. In native A2ML1t-fMG8, the MG8 domain co-elutes with the rest of A2ML1 during SEC despite furin cleavage between CUB and MG8 (panel **B**), while the MG8 domain elutes independently of the rest of A2ML1 during SEC after activation by methylamine or TEV-P (panels **C**, **D**). This experiment was performed once.

The dynamic properties of cleaved A2ML1 were examined in a 3D variability analysis^35^ of A2ML1-CC particles, since they produced the EM map with the highest resolution. This analysis indicated that between the two extreme positions, the CUB domain can move 2.8 Å, whereas the TE domain may move 4.6 Å relative to the MG-ring (Fig. 5A). Hence, in cleaved A2ML1, there is a minor degree of flexibility of the CUB and TE domains, which may help to accommodate inhibition of proteases with variable size and surface properties. Based on the local resolution maps for the three cleaved A2ML1 samples (Fig. 3C), the TE and CUB domains are likely more flexible in the absence of protease conjugation.

In contrast to this limited flexibility of the CUB and TE domains, all our structures of cleaved A2ML1 suggested that the MG8 domain of A2ML1 becomes highly flexible upon cleavage, as none of the EM maps showed significant density after residue 1321 in the CUB-MG8 linker that could be attributed to the MG8 domain. This putative flexibility of the MG8 domain in solution was further analyzed by expressing the A2ML1t-fMG8 variant containing a furin cleavage site between the CUB and MG8 domains, in addition to the TEV-P cleavage site in the bait region. The furin cleavage site was correctly processed intracellularly, causing the MG8 to migrate separately from the rest of A2ML1 in denaturing SDS-PAGE (Fig. 5B, bottom). Native A2ML1t-fMG8 contained an intact thioester as apparent from its heat-induced fragmentation prior to SDS-PAGE, which produces the TE-130 band seen in Fig. 5B–D. The MG8 domain remained associated with the rest of A2ML1 during SEC, indicating a stable non-covalent complex (Fig. 5B, top). When A2ML1t-fMG8 was activated by methylamine or TEV-P cleavage of the bait region, the MG8 domain eluted in a separate peak during SEC (Fig. 5C, D). These results show that the MG8 domain forms strong non-covalent interactions with the rest of the protein in native A2ML1 but is flexibly attached following the conformational change induced by methylamine or bait region cleavage. These data also provide further evidence that thioester aminolysis (e.g., by methylamine) changes the conformation of A2ML1 similarly to proteolysis, as is the case for C3 and A2M.

### A structure-based model for A2ML1’s inhibitory mechanism

**Fig. 6:**
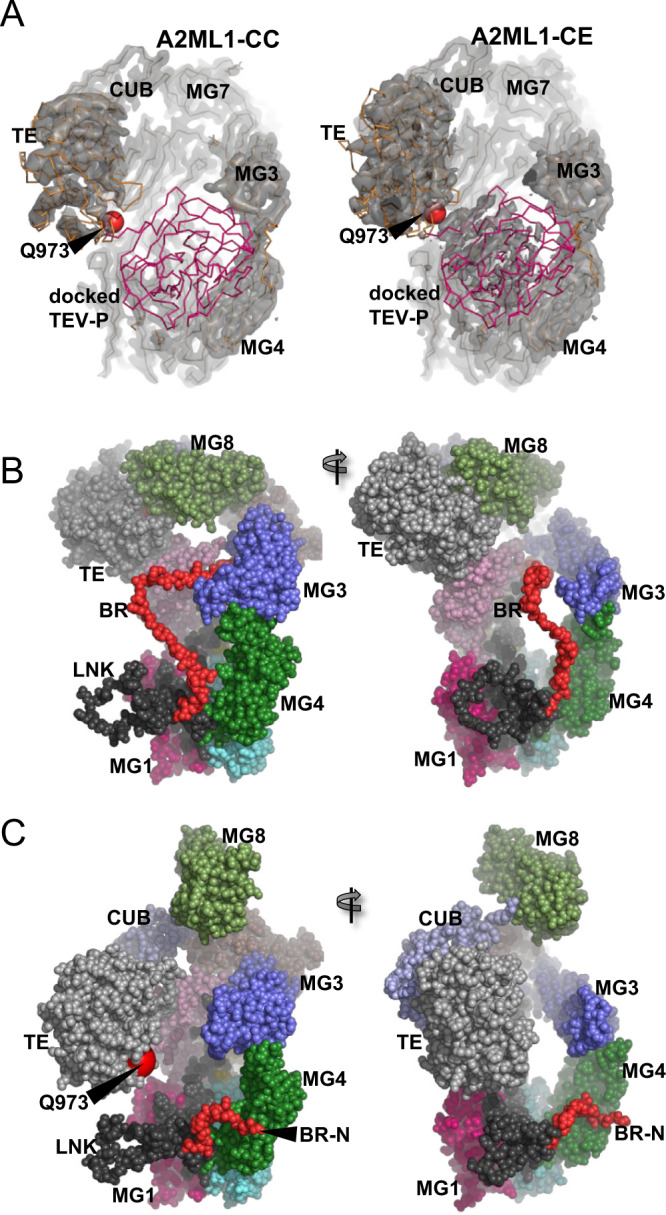
The conjugated protease has rotational freedom in an environment containing flexible extended regions. **A** Comparison of the EM maps for A2ML1-CC and A2ML1-CE with a docked TEV-P. The absence of EM map density close to the thioester suggests rotational freedom and multiple possible conjugation sites on the protease. Strikingly, weak density is present in the volume of the docked TEV-P in A2ML1-CE, possibly due to a few preferred conformations of the LNK region in the empty trap state. **B** Hypothetical full model of native A2ML1. LNK residues not modeled in any of the structures were taken from the alphafold2 prediction^57^. The bait region was modeled by hand in Coot^50^ with exposure of the experimental cleavage sites presented in Fig. 1E, F. **C** Hypothetical full model of cleaved A2ML1. Regions in LNK and the N-terminal part of the bait region (BR-N) not modeled in A2ML1-CC were modified manually in Coot starting from their conformation in panel **B**.

When proteases are conjugated by A2ML1’s thioester, they become covalently linked to Gln973 through amide or ester bonds^18^. However, no density was observed adjacent to Gln973 in our A2ML1-CC structure. Furthermore, a comparison of the EM maps for A2ML1-CC and A2ML1-CE did not reveal any difference in density elsewhere that could be assigned to the TEV-P molecule (Fig. 6A). This indicates that there are many possible conjugation sites on the surface of TEV-P, consistent with previous findings for A2M and A2ML1^36,37^, and the conjugated proteases may have many possible orientations relative to the TE domain. In cleaved A2ML1, Gln973 faces inward toward the hollow center of A2ML1. To identify regions in A2ML1 that surround a conjugated protease, we generated complete models of native A2ML1 and cleaved A2ML1 to account for flexible regions that could not be modeled in our EM and X-ray structures (Fig. 6B, C). We propose that the hollow center of A2ML1 constitutes a protease-trapping cavity in which proteases are surrounded by the TE domain on one side, the MG3 and MG4 domains on the opposite side, and a floor formed by MG domains, 1,2,5,6, and N-terminal residues in the LNK region (Fig. 6C). The partial flexibility of the CUB and TE domains relative to the MG-ring (Fig. 5A) may help to accommodate proteases of different sizes. Simple docking suggests that this cavity is suitable for a protease with the size of TEV-P (Fig. 6A). In native A2ML1 (Figs. 2G and 6B), this cavity is absent as the TE domain is more than 40 Å away. Similar cavities in each of the four subunits of the methylamine-treated A2M tetramer jointly form a large internal chamber wherein proteases are trapped^22,38^, even when they are not conjugated^27^. In contrast, our structures show that A2ML1 does not completely surround the trapped proteases, making it less clear how sequestration is achieved. Upon inspection of our model of cleaved A2ML1, we noticed that several disordered regions (in total 142 residues, i.e., 10% of A2ML1) either could not be modeled or have a weak electron density. These disordered regions, as well as glycans on Asn328 and Asn857, are oriented toward the protease-binding cavity (Fig. 6C). We hypothesize that these disordered regions contribute to the sequestering of a trapped protease through non-specific and dynamic interactions, akin to fuzzy complexes between folded proteins and intrinsically disordered proteins^39^. Some of these putative protease-interacting flexible regions are in the TE domain and are buried or shielded by interactions with the MG8 and CUB domains in native A2ML1. Bait region cleavage might also make LNK residues Pro634-Gly668 and the N-terminal end of the bait region more available for such dynamic interactions with the protease. This mesh of flexible regions is likely to limit the rotation of a trapped protease and contribute to the steric hindrance that limits access of large substrates to the active site.

Based on these observations and our biochemical characterization of A2ML1, we propose a model of protease inhibition which requires both thiol ester-mediated conjugation for protease trapping and a dynamic mesh of exposed flexible regions that help to shield the trapped protease from substrates. This model provides a possible explanation for how an A2MF monomer can inhibit proteases without completely surrounding a trapped protease. Could this mechanism extend to other monomeric relatives of A2ML1? With respect to insect TEP proteins, no protease-inhibiting ability has been demonstrated, and these proteins are believed to have complement-like functions^40^. In contrast, bacterial monomeric A2MFs from *Salmonella* and *E. coli* have been shown to inhibit proteases, and high-resolution structures of a native *Salmonella* A2MF^24^ and trypsin- or elastase-cleaved *E. coli* A2MF (ECAM)^25,26^ have been determined. Both bacterial proteins have two additional N-terminal MG domains and significantly altered MG-ring configurations compared to the animal A2MFs. Comparison of the native *Salmonella* A2MF and cleaved ECAM structures reveals a very large reorganization of the MG ring after bait region cleavage, whereas the MG ring is mostly static in animal A2MFs. The TE domain in cleaved ECAM is located in an extended position where it does not directly contact the MG ring and may be stabilized by the ECAM equivalent of the MG8 domain, which has a defined position in the structure associating with the MG ring. Furthermore, in the bacterial proteins, the additional N-terminal MG domains that are not present in animal A2MFs may contribute to shielding conjugated proteases. In contrast to A2ML1 which sequestered proteases from medium-sized substrates (≥24 kDa) in this study, ECAM only prevented trapped proteases from cleaving very large substrates (≥160 kDa)^25^. The rat A2MF protease inhibitor A1I3 can also sequester proteases from medium-sized proteins (≥22 kDa)^14^ and although its structure has not been determined, its sequence indicates it largely resembles A2ML1. These major structural and functional differences indicate that monomeric protease inhibitors from bacteria and animals are mechanistically distinct despite the dependence of both on covalent protease conjugation.

### The mechanistic steps of the protease-induced conformational change

**Fig. 7:**
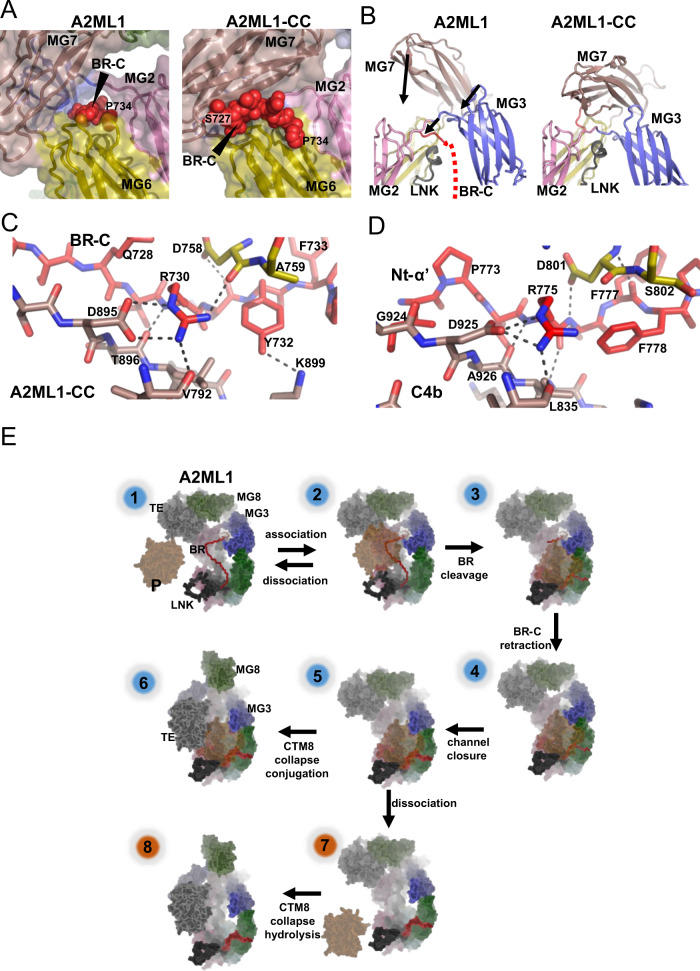
A conserved mechanism for the conformational change in A2MF proteins. **A** The BR-C retracts through the channel upon bait region cleavage and associates with a novel binding site formed by the MG6 and MG7 domains. The BR-C is presented in a back view compared to the front view in Fig. 2H. **B** Upon retraction of the BR-C, the MG3 and MG7 domains can move toward the LNK region to close the BR-C accommodating channel. Arrows indicate direction and magnitude of movement between the native (left) and cleaved state (right). **C** Magnified view of the BR-C in A2ML1-CC featuring the tight recognition of Arg730. **D** Equivalent view of the Nt-α′ region in complement C4b. **E** A hypothetical series of events from reversible protease-A2ML1 association and formation of the covalent bond (conjugation) between cleaved A2ML1 and the protease (steps 1–6). In step 3, the protease has cleaved and associates non-covalently with A2ML1. The BR-C has not retracted, and no overall conformational change has taken place. In step 4, the BR-C has retracted and the MG2-3-LNK delimited channel is open. In step 5, the channel has closed upon movement of the MG3 and MG7 domains. In step 6, the conformational change is complete, and the TE domain has formed a covalent bond to the protease. The protease may escape at any step between bait region cleavage and conjugation; steps 7–8 show one scenario where the protease escapes at a point late in the conformational change.

In native A2ML1, residues Arg730-Pro734 at the C-terminal end of the bait region (BR-C) form a plug within a channel formed by the MG2 and MG3 domains on either side, the MG2-MG3 linker from above, and the first α-helix in the LNK region from below (Fig. 2H). Based on the two highly conserved sequence stretches, Tyr214-Pro217 and Arg730-Pro734, we propose that disruption of this plug-in-channel arrangement is an essential initial event in the conformational change of A2MF proteins (Supplementary Fig. 4F). Our structures of cleaved A2ML1 all show that the plug and the preceding BR-C region must have been retracted through the channel to reach their final position between the MG6 and MG7 domains; furthermore, the channel itself has collapsed (Fig. 7A). This collapse is due to rotation of the MG7 and MG3 domains, which interact more extensively in cleaved A2ML1 than in the native state. As a result, the MG2-MG3 linker in A2ML1–CC packs against the first α-helix of the LNK region; this is prevented in the native state because the channel is occupied by BR-C residues 731–735. The rotation of the MG3 and MG7 domains (Fig. 7B) also stabilizes the position of the MG3 and MG4 domains, resulting in improved map quality for these domains for A2ML1-CC (Supplementary Fig. 7B) compared to native A2ML1 (Supplementary Fig. 3B).

In both cleaved A2ML1 and C3b, the BR-C/Nt-α′ region moves from the plug-in-channel arrangement in the native state to a different conserved binding site after proteolytic cleavage. In cleaved A2ML1, the BR-C residues Asp726-Arg730 pair with the last β-strand in the MG7 domain through hydrogen bonds. In addition, the side chain of Arg730 is firmly held in place by polar interactions with Asp758 in MG6 and Asp895 and Val792 from the MG7 domain (Fig. 7C). Furthermore, the side chain of Tyr732 engages in hydrogen bonding and van der Waals interactions with Lys899 and Leu897 in the MG7 domain. The BR-C residues Arg730 and Phe733 residues are highly conserved in A2MF proteins and participate in highly similar interactions with the MG7 domain in C3b and C4b (Fig. 7D). This similarity leads us to propose that, (i) accommodation of these two side chains between the MG6 and MG7 domains provide an important force in pulling the BR-C region out of the channel following proteolysis in the bait region, and (ii) the observed relocation of the BR-C/Nt-α′ region is a general feature of the A2MF proteins that initiates the proteolytically induced conformational change. Interestingly, Arg730 and Phe733 mutations in A2ML1 have been genetically associated with the disease otitis media^41^, along with other mutations which we predict to destabilize domain interfaces (Supplementary Note 5, Supplementary Table 4).

Based on the idea that retraction of BR-C is a key event, we propose a model for the protease-triggered conformational change in A2ML1. First, the protease associates reversibly with A2ML1 (Fig. 7E, steps 1–2). The bait region is then cleaved, and the protease must remain in place until conjugation occurs to become trapped (Fig. 7E, step 3). Upon bait region cleavage, the BR-C retracts from the LNK/MG2/MG3 channel, driven by a ratcheting interaction between the MG7 domain and the BR-C (Fig. 7E, step 4). The channel then collapses, making the conformational change irreversible and resulting in a more extensive MG3-MG7 domain interface and the establishment of interactions between the MG2-3 linker and the LNK region (Fig. 7E, step 5). Due to the MG7 reorientation, the MG8 domain is liberated, and the CUB and TE domains move to their final positions upon being released from their MG8 interactions (Fig. 7E, step 6). At any point between bait region cleavage and conjugation, the protease may dissociate without being conjugated to A2ML1; the exposed thioester in the TE domain will in this case react with water (Fig. 7E, steps 7–8). We suggest that residues Tyr630-Phe668 in the LNK region, residues N-terminal of the scissile bond in the bait region, residues Thr264-Gly274 in MG3, and residues Asp423-Arg433 in MG4 may contribute to securing the position of the protease through dynamic interactions until thioester-protease conjugation occurs. Considering the similarity of cleaved A2ML1 and the A2M-MA subunit, it is likely that these mechanistic steps are broadly conserved in A2MF of protease inhibitors. Furthermore, the conservation of key MG2-MG3 and BR-C/Nt-α′ residues, as well as the existence of the plug-in-channel arrangement in native C3/C4/C5 and Nt-α′/MG7 interactions in C3b/C4b/C5b, indicate that the conformational change occurs in a similar manner in complement factors and that the underlying mechanism arose early in an ancestral A2MF protein. As previously observed^42^ and further described in Supplementary Note 6, the A2MF proteins belong to three major branches, (i) A2M-like proteins, (ii) C3-like proteins, and (iii) CD109-like proteins (Supplementary Fig. 13). In a phylogenic analysis, we have identified two CD109-like proteins but no other A2MFs in the placozoan *Trichoplax adhaerens* but no A2MF proteins in *Porifera* sponges, suggesting that the ancestral A2MF was a CD109-like protein that originated in animals after the divergence of poriferans and eumetazoans, but before the divergence of placozoans from planulozoans. Key residues in the plug-in-channel arrangement are conserved even in these distantly related placozoan A2MF proteins, further supporting its central role in the conformational change that underlies the protease responsivity of this unique protein family.

## Methods

### Human skin sample

A deidentified normal human skin sample was collected from an adult patient undergoing plastic surgery, with full informed consent, at Aarhus University Hospital, Aarhus, Denmark. The procedure was approved by the local ethical committee, Region Midtjylland, Denmark (approval number M-20110027) and carried out according to the Declaration of Helsinki Principles.

### Materials

Bovine pancreatic trypsin, bovine pancreatic α-chymotrypsin, endoproteinase C (GluC) from *Streptococcus aureus*, thermolysin from *Geobacillus stearothermophilus*, and porcine pancreatic elastase (PPE) were from Sigma-Aldrich, while human neutrophil elastase (HNE) and human cathepsin G (CatG) were from Athens Research & Technology. Plasminogen was purified from human plasma as previously described^43^ and activated by incubating with urokinase at a 1:100 w/w ratio at 37 °C for 2 h. A1I3 was purified as previously described from rat plasma^14^ and A2M from human plasma^44^. The human skin sample was obtained from a healthy donor after skin removal surgery, and the Declaration of Helsinki protocols were followed.

### Expression and purification of recombinant A2ML1

For expression of wildtype A2ML1, a gene encoding its canonical amino acid sequence (Uniprot ID A8K2U0) was synthesized and cloned into the pcDNA3.1(+) plasmid, as described previously^18^. A2ML1t and A2ML1t-fMG8 plasmids were prepared by mutagenesis performed by Genscript. All recombinant A2Ml1 sequences are given in Supplementary Note 7. A2ML1 plasmids were transfected using the polyethylenimine method into Freestyle HEK 293-F cells (Thermo Fisher Scientific, catalog number R79007) cultivated in Freestyle 293 Expression Medium (Thermo Fisher Scientific) at a cell density of 1 × 10^6^ cells/ml. After incubation for 96 h, the culture supernatant was collected and dialyzed overnight at 4 °C into 0.1 M sodium acetate, 0.8 M NaCl, pH 7.0. The dialyzed medium was filtered and applied to a HiTrap Chelating HP column with immobilized Zn^2+^. Bound A2ML1 was eluted with 0.1 M sodium acetate, 0.15 M NaCl, 50 mM EDTA, pH 8, and the eluate was dialyzed into 20 mM HEPES, pH 7.4 overnight at 4 °C. The dialyzed sample was subjected to anion exchange on a HiTrap Q column and A2ML1 was eluted with a gradient from 0 to 40 mM NaCl. Fractions containing A2ML1 were pooled, concentrated, and subjected to SEC on a Superdex 200 increase 10/300 GL column equilibrated in HBS.

### Analytical SEC

A total of 50 µg of A2ML1 and A1I3 and 20 µg of native A2ML1t and A2ML1-CC were subjected to calibrated SEC on a Superdex 200 Increase 10/300 GL column (GE Healthcare) equilibrated in HBS.

### PAGE

Native pore-limited PAGE was run using homemade TBE buffer (89 mM Tris-HCl, 89 mM boric acid, 2 mM EDTA, pH 8.3) gels with a 10–20% acrylamide gradient. Gels were run overnight at 100 V in recirculating TBE buffer, as previously described^45^. Denaturing SDS−PAGE was performed using homemade 5–15% (w/v) acrylamide gels and the discontinuous ammediol/glycine buffer system^46^. Samples were boiled for 5 min in SDS (1%) and 35 mM dithiothreitol (DTT). Gels were stained with Coomassie Brilliant Blue.

### Identification of A2ML1 in skin extract

A 0.3 mg sample of human skin was vortexed with 0.1% NP40, incubated for 30 min rotating, and sonicated in a water bath for 15 min. The extracted proteins were run on a native pore-limited PAGE gel, and the gel lane was cut into 10 equally sized slices prior to in-gel digestion and LC-MS/MS analysis. The gel slices were shrunk using acetonitrile, reduced (0.1 M DTT), and alkylated (0.2 M iodoacetamide) in 6 M Urea, 0.1 M ammonium bicarbonate, pH 8. The sample was diluted x6 using 0.1 M ammonium bicarbonate and digested with 25 ng/ml MS grade trypsin overnight at 37 °C (Thermo Fisher Scientific). The digested peptides were purified using homemade C18 stage tips and analyzed by LC-MS/MS. Online reverse-phase high-performance liquid chromatography (RP-HPLC) was done on an EASY-nLC 1200 (Thermo Fisher Scientific) with a 5–45% gradient of acetonitrile (ACN) over 30 min. Mass spectrometry was performed on a Q Exactive Orbitrap (Thermo Fisher Scientific) running in data-dependent acquisition mode with 70,000 resolution at the MS1 level and 35,000 resolution at the MS2 level; peptides were fragmented by high-energy collision dissociation (HCD) at 27% normalized collision energy. The data were searched using the Mascot search engine (version 2.5) with fully tryptic protease cleavage, using methionine oxidation as variable modification, with mass tolerances of 10 ppm for precursors and 0.2 Da for MS2 fragment ions, a minimum peptide mass of 400 Da, and a false-discovery rate of 0.05 at both peptide and protein levels. The taxonomy was set to Homo Sapiens and the SwissProt database was used.

### Mapping of A2ML1 glycosylation pattern

Recombinant A2ML1 was digested with trypsin alone or both trypsin and chymotrypsin in solution. Digested samples were purified using homemade C18 stage tips before analysis by LC-MS/MS. Online RP-HPLC fractionation using an EASY-nLC 1200 (Thermo Fisher Scientific) was performed with a 5–45% gradient of acetonitrile (ACN) over 30 min. Mass spectrometry was performed on an Orbitrap Eclipse Tribrid (Thermo Fisher Scientific) running a glycan-triggered MS2(HCD)_MS2(EThcd) data-dependent acquisition method with a resolution of 120,000 on the MS1 level and 60,000 on both MS2 levels. HCD fragmentation used stepped collison energies (27%, 30%, 32%) whereas EThcD used automatically adjusted ETD fragmentation augmented with 10% HCD. Data were searched with the Byonic search engine (Version 3.7.13. Protein Metrics) with a standard human glycosylation database and a PEP 2D score threshold/cut-off of 0.05.

### Proteolytic digests of A2ML1

Digestions with chymotrypsin, PPE, and trypsin were done with up to a 1:1 molar ratio of protease to A2ML1. All of the reactions were carried out in HBS with and without 50 mM BAPN. The reaction mixes were incubated for 15 min at 37 °C, after which digestion was stopped by inhibiting with 2 mM phenylmethanesulfonyl fluoride (PMSF) for 15 min before SDS-PAGE analysis.

Digests with thermolysin, chymotrypsin, and HNE were carried out with a molar ratio of 1:10 of protease to A2ML1 in the presence of a 0–0.2 M titration series of BAPN. The reactions were incubated for 15 min at 37 °C before residual protease activity was inhibited by adding 2 mM PMSF or 10 mM EDTA in the case of thermolysin before SDS-PAGE analysis.

### Identification of proteins and bait region cleavage sites in SDS-PAGE bands by Edman degradation and LC-MS/MS

To identify bait region cleavage sites by different proteases using Edman degradation, samples of A2ML1 incubated with a 1:1 molar ratio of trypsin, thermolysin, HNE, cathepsin G, V8, PPE, or chymotrypsin were separated by reducing SDS-PAGE and transferred to a polyvinylidene difluoride (PVDF) membrane. The C-terminal bait region cleavage bands were cut out and applied to TFA-treated glass fiber membranes. Automated Edman degradation was performed using a PPSQ-31B protein sequencer (Shimadzu Biotech) with in-line phenylthiohydantion analysis on an LC-20AT HPLC system. Data were obtained using Shimadzu PPSQ-31B software and the sequences were determined manually from the UV 269 nm chromatograms.

To identify bait region cleavage sites by different proteases using LC-MS/MS, A2ML1 was incubated with a 1:2 molar ratio of trypsin, thermolysin, HNE, cathepsin G, V8, PPE, or chymotrypsin to A2ML1. The liberated peptides were purified using homemade C18 stage tips and analyzed by LC-MS/MS on a Q Exactive Orbitrap (Thermo Fisher Scientific), using the same methods as described for the identification of A2Ml1 in skin extract. The data were searched using Proteome Discoverer v. 2.5 with no enzyme in the case of PPE, thermolysin, and cathepsin G, while the relevant protease specificity was selected for GluC, chymotrypsin, and HNE. Up to two missed cleavages were allowed, and mass tolerances of 10 ppm for precursors and 0.02 Da for MS^2^ fragment ions were selected; the false-discovery rate was 0.05 at the peptide and protein level. The following variable modifications were considered; methionine oxidation, proline oxidation, and glutamine/asparagine deamidation along with the following variable modifications of the N-terminal; acetylation, met-loss, and acetylation combined with met-loss. Cleavage sites that were identified more than once were reported.

### Protease inhibition assays

A total of 5.8 pmol of thermolysin was reacted with 0–23.1 pmol of A2ML1 with and without 50 mM BAPN, 12 pmol of chymotrypsin was reacted with 0–67.9 pmol of A2ML1, and 17 pmol of HNE was reacted with 0–136 pmol of A2ML1 in 120 µl of 200 mM HEPES pH 7.8, 20 mM CaCl_2_ for 15 min at 37 °C. All samples were subsequently incubated with 40 µl of 0.8% azocasein (Sigma-Aldrich) for 60 min (thermolysin), 120 min (chymotrypsin), or 240 min (HNE) at 37 °C before trichloroacetic acid (TCA) was added to a final concentration of 2% to terminate the reactions and precipitate intact substrate. The precipitated substrate was removed by filtration using a MultiScreen Solvinert Filter plate (Millipore) by centrifugation at 500 × *g* for 3 min. The filtrate was collected in a 96-well Nunclon Delta Surface plate (ThermoFisher Scientific) and neutralized by addition of 2 M Tris-HCl, pH 8.8. The residual proteolytic activity was determined by measuring the absorbance at 440 nm using a FLUOstar Omega plate reader. All reactions were performed in triplicates. The inhibition of thermolysin, chymotrypsin, and HNE were investigated using a fluorescently labeled gelatin substrate, DQ™ Gelatin From Pig Skin (Invitrogen). 8.0 pmol of chymotrypsin, 1.4 pmol of thermolysin, or 1.7 pmol of HNE were reacted with a 0–8 times molar excess of A2ML1 in 50 mM HEPES, 100 mM NaCl, 5 mM CaCl_2_ pH 8 for 15 min at 37 °C. The gelatin substrate was added to a final concentration of 0.1 mg/ml and the fluorescence (excitation at 485 nm and emission at 520 nm) of the digestion products of DQ gelatin were measured in a FLUOstar Omega plate reader (BMG LABTECH) after 4 min (thermolysin), 10 min (chymotrypsin), or 20 min (HNE) at 37 °C. All reactions were performed in triplicates.

### Crystallization of native A2ML1

Native A2ML1 was incubated with a 1:10 (w/w) ratio of bacterially expressed GST-tagged PNGase F, MBP-tagged Endo F2, and MBP-tagged Endo F3 to A2ML1 at room temperature for 24 h. The sample was concentrated prior to SEC on a Superdex 200 10/300 column. Fractions from SEC containing pure A2ML1 were pooled and concentrated to 5–6.5 mg/ml followed by centrifugation for 5 min immediately prior to crystallization. A2ML1 was screened for crystallization in sitting-drop vapor diffusion experiments using the commercial screens PEGRx and PEGIon (Hampton research). Drops of 150 nl A2ML1 mixed with 150 nl reservoir solution were generated with an Oryx4 crystallization robot (Douglas Instruments) and equilibrated against 50 µl reservoir solution in 96-well Swissci MRC crystallization plates at room temperature. The final optimal reservoir condition contained 0.1 M potassium phosphate dibasic, 10% PEG 3350 pH 9.2 in drops of 0.9 µl A2ML1 mixed with 0.1 µl seeds and 1 µl reservoir buffer. Crystals were soaked into the reservoir solution added 25% (v/v) ethylene glycol as cryoprotectant followed by flash cooling in liquid nitrogen. Data collection with MXCUBE3 from flash-frozen crystals was performed at BioMAX (MAX IV, Lund) at 100 K and processed with XDS^47^ built 20180808. Phenix programs from the 1.19.2-4158 version used for crystallographic and cryo-EM structural work. The structure was determined in phenix.phaser^48^ using two complementary density search models covering MG1-6 and CTM8, respectively, excised from a preliminary 3.8 Å cryo-EM map. After density modification with phenix.density_modification^49^, a near complete EM structure of native A2ML1 was placed domain-wise in the two molecules within the asymmetric unit present in the crystallographic map. The resulting model was in an iterative manner manually rebuild in Coot 0.9.3^50^ and refined in phenix.refine^51^ using positional, TLS, non-crystallographic symmetry restraints, and grouped B-factor refinement.

### Preparation of samples for cryo-EM

Recombinant native A2ML1 was concentrated to 1 mg/ml using a Vivaspin 2 centrifugal concentrator unit with a 30-kDa cut-off (GE Healthcare) before grid preparation for cryo-EM. To generate the A2ML1-CC and A2ML1-CA samples 600 µg of A2ML1t was incubated overnight at room temperature with a 1:1 molar ratio of A2ML1 to TEV-P in HBS with or without 200 mM BAPN. The cleaved A2ML1 samples were prepared for hydrophobic interaction chromatography on a HiTrap Phenyl HP 1 ml column by addition of 1.6 M sodium sulfate pH 7.4 to a final concentration of 800 mM. The samples were loaded onto the column and subsequently eluted with a gradient from 560 mM sodium sulfate pH 7.2 to 0 mM sodium sulfate pH 7.2.

To prepare a resin with immobilized TEV-P for preparation of the A2ML1-CE sample, 0.5 g of CNBr-activated Sepharose 4B beads (Sigma-Aldrich) were incubated with 1 mM HCl for 30 min at room temperature. The beads were washed 5 times in 1 mM HCl followed by 1 time in coupling buffer (0.1 M NaHCO_3_, 0.5 M NaCl pH 8.3) followed by incubation with 1.3 mg/ml of TEV-P in coupling buffer for 1.5 h. Beads were subsequently washed in coupling buffer and incubated with blocking buffer (0.1 M Tris-HCl pH 8) for 2 h followed by washing 6 times in coupling buffer. For cleavage, 500 µl TEV-P coupled beads were washed in HBS 3 times, incubated with 5 mM DTT in HBS for 15 min and washed in HBS 3 times. An amount of 800 µg of A2ML1t was incubated with the beads in HBS in the presence of 100 mM BAPN for 2 h. The cleaved A2ML1 sample was subjected to SEC on a Superdex 200 increase 10/300 GL column equilibrated in HBS. Fractions containing cleaved A2ML1 were pooled and concentrated prior to cryo-EM.

### Cryo-EM

Copper grids CF-1.2/1.3-3Cu-50 (Protochips) were glow discharged with a current of 15 mA for 45 s using a GloQube (Quorum Technologies) system. Cryo-EM samples were prepared with a Mark IV Vitrobot system (Thermo Fisher Scientific). Briefly, 3 µL of sample were applied to each grid, the temperature during grid preparation was 4 °C and the relative humidity in the chamber was set to 100%. The blot force was 0 or 1 and the blot time 4.5 s. Data collection using the EPU 2.7 software was done on a Titan Krios G3i (Thermo Fisher Scientific) equipped with a K3 detector (Gatan) with a Bioquantum (Gatan) energy filter (slit width of 20 eV) and a C2 aperture with a 50 μM diameter, spot size 3 and a beam diameter of 0.98–1.12 μm. Data were collected in counted super-resolution mode with gain correction on the fly followed by 2x binning in EPU resulting in movies output at physical pixel size (0.647 Å/pixel at the magnification used). For processing of all the datasets, the collected movies were imported into CryoSparc v3 (Structura Biotechnology Inc), with the following parameters: 300 kV accelerating voltage, 2.7 mm spherical aberration, 0.1 amplitude contrast fraction, 130,000 nominal magnification (calibrated pixel size of 0.647 Å/pixel). All subsequent processing steps were carried out in CryoSparc v3. Key parameters and statistics for data collection are presented in Supplementary Table 1.

In general, the workflow for image analysis and 3D reconstruction was the same for all four data sets. The specific steps are described in Supplementary Fig. 3A for native A2ML1, Supplementary Fig. 6A for A2ML1-CE, Supplementary Fig. 7A for A2ML1-CC, and Supplementary Fig. 8A for the A2ML1-CA dimer. For each movie, motion correction and CTF parameters were estimated in patches. Particles were picked through blob picker followed by 2D classification to generate templates for template picker. Template-picked particles were cleaned with another 2D classification, although this step was skipped for A2ML1-CC. For further cleaning of particles ab initio volumes were generated by 3D classification, followed by heterogenous refinement. Eventually, non-uniform refinement was applied on the cleaned particle stacks. In the three datasets with TEV-P cleaved A2ML1 where both monomers and dimers are present, these were separated by 2D classification, multi-class ab initio reconstructions and heterogeneous refinement as indicated in Supplementary Figs. 6, 7, and 8.

An atomic model for A2ML1-CC was generated by homology modeling starting from the structures methylamine-treated A2M in pdb entry 4ACQ (22). After overall fitting in Chimera-X, each domain of the starting model was placed by rigid-body refinement in Coot and manually rebuilt to the density. The model was refined in phenix.real_space_refine using rigid-body refinement, positional refinement and grouped B-factor refinement^52^ and rebuilt with Coot in an iterative manner until convergence. Starting models for the A2ML1-CE monomer and the A2ML1-CA dimer were generated from the structure of A2ML1-CC and first fitted by rigid-body refinement with phenix.real_space_refine into the relevant map after which the model was fitted in Coot and refined again in an iterative manner until convergence. A starting model for native A2ML1 was constructed from domains taken from the A2ML1-CC structure combined with a homology model for the TE domain based on the structure of native C3 (pdb entry 6RU5^53^) and the crystal structure of the isolated MG8 domain of bovine A2M (pdb entry 1AYO^54^). The model was refined and rebuilt as described for A2ML1-CC. Model quality was evaluated with phenix.validation_cryoem^55^. The structures of native A2ML1 and A2ML1-CE were refined to a slightly lower maximum resolution compared to the map resolution estimated by cryosparc as presented in Supplementary Table 1 due to very large differences in map resolution throughout the molecule.

All Figures were prepared with PYMOL 2.3.0, https://pymol.org/pymol.html, or Chimera-X 1.1 or 1.3^56^. An intact model of native A2ML1 was prepared starting from the cryo-EM structure of A2ML1. Unmodelled regions were adopted from the X-ray and alphafold2^57^ structures of A2ML1, and the bait region was manually adjusted to the putative path. Proper geometry was enforced by real-space refinement of distorted regions in Coot. To create the equivalent structure of protease-cleaved A2ML1, missing regions in A2M-CC were replaced as done for the native structure, where after residues 691–710 were rotated to a more exposed position to illustrate bait region collapse. Finally, the conformation of LNK residues not observed in A2ML1-CC was modified by hand in Coot compared to their conformation in the model of full native A2ML1.

### Phylogenetic analysis of A2MF sequences

Human A2MF protein sequences were downloaded from NCBI. Human A2ML1 (XP_011518868) and human CD109 (Q6YHK3) were used as queries for NCBI blastp^58^ searches against annotations from genome sequences of the placazoa Trichoplax (*Trichoplax adhaerens*), the cniderias swiftia (*Swiftia exserta*) and starfish (*Asterias rubens*), the deuterostomes littorina snail (*Littorina littorea*) and horseshoe crab (*Limulus polyphemus*), the urochordate vase tunicate (*Ciona intestinalis*) and the nematode C. elegans (*Caenorhabditis elegans*). Only full-length coding sequences were considered. Using CLC Main Workbench 21.0.5 (QIAGEN), the sequences were aligned with Clustal Omega^59^. A maximum-likelihood phylogeny was reconstructed on the multiple sequence alignment. Bootstrap values from 1000 bootstrap replicates were computed. To investigate A2MFs in Porifera, tblastn searches limited to nucleotide entries assigned to Porifera taxonomy (taxid: 6040) were conducted using the *Trichoplax* CD109-like protein (NCBI: XP_002111588.1) as query. Searching non-redundant (NR) and whole genome shotgun contigs (WGS) databases gave no results, whereas transcriptome shotgun assembles (TSA and the database assembled by Manousaki et al.^60^ returned several hits (TRINITY_DN158914_c0_g1_i1, ACY74611, GIUN01026505.1, GIUN01023177.1, HBWD01391903.1). Using these hits as queries in blastx searches of the entire NR database revealed that these sequences most likely originate from other taxa (cnidaria, rat, and mussels) and therefore do not support the presence of A2MFs in Porifera. No Porifera reference genome has been sequenced to date. To investigate A2MFs in fungi and plant kingdoms, tblastn searches limited to nucleotide entries assigned to Fungi (taxid:4751) and green plants (taxid:33090) were conducted, again using the *Trichoplax* CD109-like protein (NCBI: XP_002111588.1) as query. No hits were returned for fungi. The five hits returned for plants (XP_030540977.1, XP_030549320.1, XP_002499844.1, GHP02778.1, XP_007512798.1) were then used as queries in blastp searches of the entire NR database. This analysis revealed that two sequences from land plants (XP_030540977.1, XP_030549320.1) most likely originate from metazoan taxa (mites) and are hence not supporting the presence of A2MPs in land plants. However, the three hits originating from green algae (XP_002499844.1, GHP02778.1, and XP_007512798.1, originating from the Mamiellales species *Micromonas commoda*, *Pycnococcus provasolii*, and *Bathycoccus prasinos*, respectively) most likely represent authentic sequences. The lack of wider taxonomic presence of A2MFs in Mamiellales genomes indicates that these sequences probably have been obtained through horizontal transfer with metazoan origin.

### Reporting summary

Further information on research design is available in the Nature Research Reporting Summary linked to this article.

## Supplementary information

Supplementary Information

Reporting Summary

## Data Availability

All the relevant data have been deposited or are available from the corresponding authors upon request. Source data are provided in the accompanying Source Data file. Sequences for the recombinant A2ML1 proteins are given in Supplementary Note 7. All mass spectrometry data have been deposited to the ProteomeXchange Consortium via the PRIDE partner repository^61^ with the dataset identifier PXD032010. New atomic structures for the EM and X-ray structures are deposited at the Protein Data Bank as entries 7Q1Y (X-ray native A2ML1), 7Q5Z (EM native A2ML1), 7Q60 (EM A2ML1-CE monomer), 7Q61 (EM A2ML1-CC monomer), and 7Q62 (EM A2ML1-CA dimer). Previously published structures used in this article are available at the Protein Data bank: 4ACQ (A2M-MA), 7S63 (native A2Moo), 6RU5 (native C3), 1AYO (bovine A2M MG8). The cryo-EM maps corresponding to the EM-derived structures are deposited at the EMDB (https://www.ebi.ac.uk/emdb/) as entries EMD-13847 (native A2ML1), EMD-13848 (A2ML1-CE monomer), EMD-13849 (A2ML1-CC monomer), EMD-13850 (A2ML1-CA dimer). Source data are provided with this paper.

## Source data

Source Data
