# Supplementary material for: Cryo-EM structures of human A2ML1 elucidate the protease-inhibitory mechanism of the A2M family
Source: Nat Commun. 2022 May 31;13:3033. doi: 10.1038/s41467-022-30758-x (PMC9156758; doi:10.1038/s41467-022-30758-x)
Supplement: Supplementary file 1 — Supplementary Information [file 41467_2022_30758_MOESM1_ESM.pdf]

Supplementary Information

Cryo-EM structures of human A2ML1 elucidate the protease-inhibitory mechanism of the A2M family.

Supplementary Note 1.

Native and protease-A2ML1 complexes are monomeric. The cysteine residues involved in inter-subunit disulfide bond formation in tetrameric A2M (Cys278, Cys431) and dimeric PZP (Cys277, Cys427) are not conserved in A2ML1, suggesting that it is a monomer. The absence of disulfide-bonded dimers has been experimentally confirmed[1], but noncovalent oligomerization has not been ruled out. Our recombinant A2ML1 migrated similarly to A1I3[2], a monomeric A2MF inhibitor from rat, during native pore-limited PAGE and size exclusion chromatography (SEC), suggesting that A2ML1 is solely expressed as a monomer (Sup. Fig. 1A-B). In addition, authentic A2ML1 was extracted from human skin and analyzed by native pore-limited PAGE (Sup. Fig. 1E). Directed by the migration of the monomeric A1I3 and the tetrameric A2M, A2ML1 was identified by in-gel digestion and LC-MS/MS-based peptide identification migrating at a distance that corresponds to an A2ML1 monomer (Sup. Fig. 1E). Based on these observations, we hypothesized that the inhibitory unit of A2ML1 is also the monomer.

To obtain specific cleavage at a single site in the bait region for structure determination, we introduced a tobacco etch virus protease (TEV-P) cleavage recognition sequence (ENLYFQS) at positions 700-706 in A2ML1. This recombinant A2ML1-TEV also migrated as a monomer in SEC when cleaved by TEV-P (Sup. Fig. 1C). Furthermore, wildtype recombinant A2ML1 cleaved by thermolysin also migrated as a monomer in native pore-limited PAGE regardless of whether thermolysin was conjugated to A2ML1 (Sup. Fig. 1D). The slightly slower migration of methylamine- and thermolysin-activated A2ML1 likely reflects a major conformational change upon A2ML1 activation. Overall, these experiments did not indicate oligomerization of either native A2ML1 or protease-cleaved A2ML1, suggesting that the monomer is the functional unit and sufficient for protease inhibition.

Supplementary Note 2.

Non-specific binding of TEV-P. We did not expect TEV-P to associate non-covalently with A2ML1, as was observed in the A2ML1-CA sample (Fig. 3B), because protease inhibition had been shown to depend on covalent protease trapping. To investigate the relevance of this non-covalent protease interaction, we incubated A2ML1 with Cy5-labelled thermolysin and chymotrypsin, with and without BAPN, and then purified A2ML1 by SEC. Cy5-thermolysin, but not Cy5-chymotrypsin, co-migrated with A2ML1 during SEC in comparable amounts both with and without covalent protease conjugation (Sup. Fig. 5A-B). However, A2ML1 did not non-covalently trap Cy5-thermolysin during bait region cleavage and the subsequent conformational change. This was concluded because cleaved A2ML1 remained able to associate with Cy5-thermolysin added after its cleavage (Sup. Fig. 5D). The inability of Cy5-chymotrypsin to associate with cleaved A2ML1 was probably due to chymotrypsin degradation of A2ML1, as A2ML1 that was pre-cleaved by non-labelled chymotrypsin was unable to associate with Cy5-thermolysin afterwards and no longer showed the full-size bait region-cleaved product bands in

SDS-PAGE (Sup. Fig. 5C). When A2ML1 was incubated with inactive Cy5-thermolysin, Cy5-thermolysin did not co-elute with A2ML1 during SEC, indicating that this non-covalent association is specific to A2ML1's activated conformation (Sup. Fig. 5E). These results, alongside the activity of proteases incubated with A2ML1 in the presence of BAPN towards substrates from 20-720 kDa in size (Fig 1A-B, Sup. Fig. 2B), indicate that non-covalently associated proteases do not interact with activated A2ML1 in the same manner as proteases that are covalently trapped during A2ML1's conformational change and are not inhibited to any meaningful extent. These non-specific interactions parallel those previously shown for A2M, which in its activated conformation will bind proteases in a non-sequestered position on its surface [3] as well as cytokines such as TGFβ and PDGF-BB [4].

Supplementary Note 3.

A fraction of TEV-P cleaved A2ML1 formed dimers on EM grids. In the three different samples of TEV-P cleaved A2ML1, we consistently observed that roughly 1/6 of the particles picked from EM micrographs were dimers. Considering that the concentration of TEV-P cleaved A2ML1 during formation of the grids was 1 mg/ml, a dissociation constant (K_D) of 15 μM can be estimated for the dimer-monomer equilibrium. Consistent with this low-affinity K_D value, dimers were not noticeable during SEC analysis of TEV-P cleaved A2ML1 at a protein concentration of 0.1 mg/ml (Sup. Fig. 1C). The structure of the dimer found in the A2ML1-CA sample was determined at a resolution of 3.2 Å (Sup. Fig. 7A-B, Sup. Fig. 8B, and Sup. Table 1). The cross-correlation between map and model was higher for the A2ML1-CA dimer (0.77) compared to the A2ML1-CC and A2ML1-CE dimers although structurally they all were very similar. The overall conformation of the MG-ring in each of the two monomers participating in the A2ML1-CA dimer is close to that of the A2ML1-CC monomer's structure, with overall RMSD values on the Cα atoms below 1 Å. However, the density map of the CUB and TE domains was weaker in one of the dimer-participating monomers in all three TEV-P cleaved A2ML1 samples compared to the monomer and further modelling was not attempted (Sup. Fig. 8A). In contrast, the other dimer-participating monomer, could be modelled and a RMSD of 1Å was obtained with the A2ML1-CC monomer structure. Hence, within the cleaved A2ML1 dimer, one monomer has a conformation close to that of the A2ML1-CC monomer, whereas in the second monomer, the CUB and TE domains must have more variation in their orientation relative to the MG-ring and the MG7 domain.

Within the dimer, the two molecules are related by an almost perfect two-fold rotation axis perpendicular to the plane of the dimer (Sup. Fig. 8A, 8C). Analysis with PISA suggests a rather larger buried surface area of 2500 Å^2 with a P-value of 0.40. The dimer is maintained by side chain-main chain and side chain-side chain interactions between the MG1 domain in one monomer and the MG6 domain in the opposite monomer (Sup. Fig. 8D). In MG1, Tyr68, His77, and Ser80 engage in multiple inter-subunit contacts, whereas in MG6 Pro743, Asn746, Glu750, and His753 contribute significantly to contacting the opposite monomer. An aromatic core of the interface is formed by the interaction of

Tyr68, His75, and His77 with His753 from the opposite subunit (Sup. Fig. 8D). It is not clear whether this dimerization is a conserved feature in A2ML1 from mammals after analysis of the conservation of these residues within the interface (Sup. Fig. 8E).

Cleaved A2ML1's dimer arrangement is not related to the dimerization interfaces present in the structure of A2M-MA where subunits are interfaced to each other non-covalently through either TE-TE and TE-MG1 domain interactions or LNK-LNK interactions, or covalently through MG3-MG4 disulfides[5]. Dimerization of complement C3b is also well established, but the two major axes of the monomers are parallel in the classical C3b crystal packing dimer [6], whereas the major axes are anti-parallel for cleaved A2ML1. In addition, dimer particles were not observed in the native cryo-EM sample and no monomer-monomer interaction resembling that of the TEV-P cleaved A2ML1 dimer is present in the crystal packing of native A2ML1. This lack of dimerization in native A2ML1 is probably due to a difference in the orientation of the MG1 domain by 10° relative to the MG6 domain, compared to cleaved A2ML1. This also makes heterodimerization of one native A2ML1 molecule with another cleaved A2ML1 molecule unlikely. In summary, dimerization appears to be exclusive to cleaved A2ML1 and its dimer organization is unlike that of other activated A2MF proteins.

Supplementary Note 4.
Post-translational modifications observed in A2ML1 structures. The structures of native and TEV-P-cleaved A2ML1 allow a validation of the predicted disulfide bridge pattern. The disulfide bridge pattern (Fig. 2A) is confirmed in structures of both A2ML1 and A2ML1-CC . As an example, two closely located disulfide bridges in the MG7 domain, Cys819-Cys847 and Cys845-Cys881, can be distinguished without ambiguity in the A2ML1-CC map (Fig. 3F). The presence of Asn-linked glycans in A2ML1 has previously been shown[1], but which of the 12 predicted sites carry glycans is unknown. To obtain experimental evidence for such Asn-linked glycans by an orthogonal approach, recombinant A2ML1 was digested with trypsin alone or in combination with chymotrypsin and subsequently analyzed by LC-MS/MS. The identified N-glycosylation sites confirmed by this MS analysis (Sup. Table 3) are located in MG1 (Asn104 and Asn120), MG3 (Asn281), MG4 (Asn409), MG6 (Asn609), MG7 (Asn857 and 867), and the TE domain (Asn1020). In the EM and X-ray maps, we consistently observed glycan modification of asparagine residues 120, 328, 609, 857, and 867.

Supplementary Note 5.
Structural interpretation of disease-linked A2ML1 mutations. Otitis media is a common disorder of the middle ear in children, and even though there is strong evidence for the existence of genetic dispositions to this disease, only a few associated loci have been mapped [7]. Recently, rare variants of A2ML1 have been found associated with otitis media susceptibility, particularly in an indigenous Filipino population, although some cases were also reported in populations from other parts of the world

[8,9]. Several disease-associated A2ML1 variants giving rise to intact proteins with single site mutations (Sup. Table 4). As one prominent example, the phenotype induced by mutations of the highly conserved residues Arg730 and Phe733 are likely to affect the stability of native A2ML1, as they are located in the BR-C region that runs through a narrow interdomain channel (Fig. 2H). As discussed below, in the activated state these two residues are anchored between the MG6 and MG7 domains. Their mutation may therefore perturb the conformational change induced upon protease cleavage affecting the inhibitory function of A2ML1. Other single-point mutations may also have consequences based on their role in the structure of A2ML1 and A2ML1-CC (Sup. Fig. 9). In summary, we predict that the variants Q561H, L671P, and A991P are likely to destabilize domain interfaces, variants G777R and A1431V are incompatible with correct folding of MG6 and MG8, and the variant R1001W may destabilize an N-terminal region in the TE domain.

Supplementary Note 6.

Evolution of the A2MF protein superfamily. In order to elucidate the ancestral A2MF protein, we investigated A2MF sequences in the genomes of distantly related organisms. A2MF proteins have almost exclusively been identified in animals, although they are also found in some bacteria[10] and green algae (Mamiellales) species[11], likely as the result of horizontal gene transfer. A2MF protease inhibitors from arthropod (*Limulus polyphemus*) and mollusc (*Octopus vulgaris*) have been functionally characterized[12,13], demonstrating that A2MF proteins are widespread in bilaterian animals. We therefore investigated A2MF proteins in even more distant animals by searching annotated genomes from two cnidarian species (*Swiftia exserta* and the starfish *Asterias rubens*) and the placozoan *Trichoplax adhaerens*, as well as four additional bilaterian species (the nematode *Caenorhabditis elegans,* the snail *Littorina littorea*, the horseshoe crab *Limulus polyphemus*, and the vase tunicate *Ciona intestinalis*). The resulting phylogeny can be divided into three major branches, (i) A2M-like proteins, (ii) C3-like proteins, and (iii) CD109-like proteins (Sup. Fig. 12). All three branches were represented in cnidarians, showing that the ancestral A2MF and its gene duplication predates the divergence of bilaterians and cnidarians, in agreement with previous analysis[14]. Two CD109-like proteins, but no other A2MFs, were found in the placozoan *Trichoplax*, whereas no convincing evidence of A2MF proteins was found in *Porifera* (sponges). These results suggest that the ancestral A2MF originates after the divergence of poriferans and eumetazoans, but before the divergence of placozoans from planulozoans (bilaterians and cnidarians). Furthermore, they suggest that the first A2MF was CD109-like, as proposed previously[15]. This interpretation assumes that the inability to identify non-CD109-like A2MFs from *Trichoplax* and A2MFs in general in *Porifera* is in fact due to their absence, and not issues with genomic coverage. While the *Trichoplax* genome used was high-quality[16], the *Porifera* genomic data was incomplete and contaminated with sequences from other species.

Human CD109 is a monomeric A2MF protein with a well-substantiated role as a TGFβ co-receptor [17-19]. It is membrane-bound through a GPI anchor, as is predicted for one of the *Trichoplax* CD109-like proteins (NCBI: XP_002111588.1). In a primitive animal lacking a circulatory system, membrane-bound A2MFs may be advantageous in that they will not diffuse away from the animal, in contrast to secreted A2MFs. Human CD109 has not been characterized as an A2MF protein further than demonstration of its thioester and bait region cleavage by proteases [17], and it is not known whether CD109-like A2MFs can inhibit proteases. This makes it difficult to speculate on the function of the putatively CD109-like ancestral A2MF protein. The *Trichoplax* CD109-like sequences support the ubiquity of the plug-in-channel arrangement, which we describe for A2ML1, as sequences in the MG2-MG3 linker, first LNK alpha-helix, and BR-C region are highly conserved even in these distantly related proteins. Our structures pinpoint the plug-in-channel motif as central to the structure and conformational change of A2MFs that allows this unique family of proteins to fulfill diverse roles as protease-responsive effector or signal-propagating proteins.

Supplementary Note 7.

Sequences of recombinant A2ML1 proteins.

>A2ML1 DNA sequence

ATGTGGGCTCAGCTCCTTCTAGGAATGTTGGCCCTATCACCAGCCATTGCAGAAGAACTTCCAAACTACCTGGT
GACATTACCAGCCCGGCTAAATTTCCCCTCCGTTCAGAAGGTTTGTTTGGACCTGAGCCCTGGGTACAGTGAT
GTTAAATTCACGGTTACTCTGGAGACCAAGGACAAGACCCAGAAGTTGCTAGAATACTCTGGACTGAAGAAG
AGGCACTTACATTGTATCTCCTTTCTTGTACCACCTCCTGCTGGTGGCACAGAAGAAGTGGCCACAATCCGGGT
GTCGGGAGTTGGAAATAACATCAGCTTTGAGGAGAAGAAAAAGGTTCTAATTCAGAGGCAGGGGAACGGCA
CCTTTGTACAGACTGACAAACCTCTCTACACCCCAGGGCAGCAAGTGTATTTCCGCATTGTCACCATGGATAGC
AACTTCGTTCCAGTGAATGACAAGTACTCCATGGTGGAACTACAGGATCCAAATAGCAACAGGATTGCACAGT
GGCTGGAAGTGGTACCTGAGCAAGGCATTGTAGACCTGTCCTTCCAACTGGCACCAGAGGCAATGCTGGGCA
CCTACACTGTGGCAGTGGCTGAGGGCAAGACCTTTGGTACTTTCAGTGTGGAGGAATATGTGCTGCCGAAGT
TTAAGGTGGAAGTGGTGGAACCCAAGGAGTTATCAACGGTGCAGGAATCTTTCTTAGTAAAAATTTGTTGTAG
GTACACCTATGGAAAGCCCATGCTAGGGGCAGTGCAGGTATCTGTGTGTCAGAAGGCAAATACTTACTGGTA
TCGAGAGGTGGAACGGGAACAGCTTCCTGACAAATGCAGGAACCTCTCTGGACAGACTGACAAAACAGGAT
GTTTCTCAGCACCTGTGGACATGGCCACCTTTGACCTCATTGGATATGCGTACAGCCATCAAATCAATATTGTG
GCTACTGTTGTGGAGGAAGGGACAGGTGTGGAGGCCAATGCCACTCAGAATATCTACATTTCTCCACAAATG
GGATCAATGACCTTTGAAGACACCAGCAATTTTTACCATCCAAATTTCCCCTTCAGTGGGAAGATAAGAGTTAG
GGGCCATGATGACTCCTTCCTCAAGAACCATCTAGTGTTTCTGGTGATTTATGGCACAAATGGAACCTTCAACC
AGACCCTGGTTACTGATAACAATGGCCTAGCTCCCTTTACCTTGGAGACATCCGGTTGGAATGGGACAGACGT

TTCTCTGGAGGGAAAGTTTCAAATGGAAGACTTAGTATATAATCCGGAACAAGTGCCACGTTACTACCAAAAT
GCCTACCTGCACCTGCGACCCTTCTACAGCACAACCCGCAGCTTCCTTGGCATCCACCGGCTAAACGGCCCCTT
GAAATGTGGCCAGCCCCAGGAAGTGCTGGTGGATTATTACATCGACCCGGCCGATGCAAGCCCTGACCAAGA
GATCAGCTTCTCCTACTATTTAATAGGGAAAGGAAGTTTGGTGATGGAGGGGCAGAAACACCTGAACTCTAA
GAAGAAAGGACTGAAAGCCTCCTTCTCTCTCTCACTGACCTTCACTTCGAGACTGGCCCCTGATCCTTCCCTGG
TGATCTATGCCATTTTTCCCAGTGGAGGTGTTGTAGCTGACAAAATTCAGTTCTCAGTCGAGATGTGCTTTGAC
AATCAGGTTTCCCTTGGCTTCTCCCCCTCCCAGCAGCTTCCAGGAGCAGAAGTGGAGCTGCAGCTGCAGGCAG
CTCCCGGATCCCTGTGTGCGCTCCGGGCGGTGGATGAGAGTGTCTTACTGCTTAGGCCAGACAGAGAGCTGA
GCAACCGCTCTGTCTATGGGATGTTTCCATTCTGGTATGGTCACTACCCCTATCAAGTGGCTGAGTATGATCAG
TGTCCAGTGTCTGGCCCATGGGACTTTCCTCAGCCCCTCATTGACCCAATGCCCCAAGGGCATTCGAGCCAGC
GTTCCATTATCTGGAGGCCCTCGTTCTCTGAAGGCACGGACCTTTTCAGCTTTTTCCGGGACGTGGGCCTGAAA
ATACTGTCCAATGCCAAAATCAAGAAGCCAGTAGATTGCAGTCACAGATCTCCAGAATACAGCACTGCTATGG
GTGCAGGCGGTGGTCATCCAGAGGCTTTTGAGTCATCAACTCCTTTACATCAAGCAGAGGATTCTCAGGTCCG
CCAGTACTTCCCAGAGACCTGGCTCTGGGATCTGTTTCCTATTGGTAACTCGGGGAAGGAGGCGGTCCACGTC
ACAGTTCCTGACGCCATCACCGAGTGGAAGGCGATGAGTTTCTGCACTTCCCAGTCAAGAGGCTTCGGGCTTT
CACCCACTGTTGGACTAACTGCTTTCAAGCCGTTCTTTGTTGACCTGACTCTCCCTTACTCAGTAGTCCGTGGGG
AATCCTTTCGTCTTACTGCCACCATCTTCAATTACCTAAAGGATTGCATCAGGGTTCAGACTGACCTGGCTAAA
TCGCATGAGTACCAGCTAGAATCATGGGCAGATTCTCAGACCTCCAGTTGTCTCTGTGCTGATGACGCAAAAA
CCCACCACTGGAACATCACAGCTGTCAAATTGGGTCACATTAACTTTACTATTAGTACAAAGATTCTGGACAGC
AATGAACCATGTGGGGGCCAGAAGGGGTTTGTTCCCCAAAAGGGCCGAAGTGACACGCTCATCAAGCCAGTT
CTCGTCAAACCTGAGGGAGTCCTGGTGGAGAAGACACACAGCTCATTGCTGTGCCCAAAAGGAAAGGTGGCA
TCTGAATCTGTCTCCCTGGAGCTCCCAGTGGACATTGTTCCTGACTCGACCAAGGCTTATGTTACGGTTCTGGG
AGACATTATGGGCACAGCCCTGCAGAACCTGGATGGTCTGGTGCAGATGCCCAGTGGCTGTGGCGAGCAGA
ACATGGTCTTGTTTGCTCCCATCATCTATGTCTTGCAGTACCTGGAGAAGGCAGGGCTGCTGACGGAGGAGAT
CAGGTCTCGGGCAGTGGGTTTCCTGGAAATAGGGTACCAGAAGGAGCTGATGTACAAACACAGCAATGGCTC
ATACAGTGCCTTTGGGGAGCGAGATGGAAATGGAAACACATGGCTGACAGCGTTTGTCACAAAATGCTTTGG
CCAAGCTCAGAAATTCATCTTCATTGATCCCAAGAACATCCAGGATGCTCTCAAGTGGATGGCAGGAAACCAG
CTCCCCAGTGGCTGCTATGCCAACGTGGGAAATCTCCTTCACACAGCTATGAAGGGTGGTGTTGATGATGAGG
TCTCCTTGACTGCGTATGTCACAGCTGCATTGCTGGAGATGGGAAAGGATGTAGATGACCCAATGGTGAGTC
AGGGTCTACGGTGTCTCAAGAATTCGGCCACCTCCACGACCAACCTCTACACACAGGCCCTGTTGGCTTACATT
TTCTCCCTGGCTGGGGAAATGGACATCAGAAACATTCTCCTTAAACAGTTAGATCAACAGGCTATCATCTCAG
GAGAATCCATTTACTGGAGCCAGAAACCTACTCCATCATCGAACGCCAGCCCTTGGTCTGAGCCTGCGGCTGT
AGATGTGGAACTCACAGCATATGCATTGTTGGCCCAGCTTACCAAGCCCAGCCTGACTCAAAAGGAGATAGC
GAAGGCCACTAGCATAGTGGCTTGGTTGGCCAAGCAACACAATGCATATGGGGGCTTCTCTTCTACTCAGGAT

ACTGTAGTTGCTCTCCAAGCTCTTGCCAAATATGCCACTACCGCCTACATGCCATCTGAGGAGATCAACCTGGT

TGTAAAATCCACTGAGAATTTCCAGCGCACATTCAACATACAGTCAGTTAACAGATTGGTATTTCAGCAGGATA

CCCTGCCCAATGTCCCTGGAATGTACACGTTGGAGGCCTCAGGCCAGGGCTGTGTCTATGTGCAGACGGTGTT

GAGATACAATATTCTCCCTCCCACAAATATGAAGACCTTTAGTCTTAGTGTGGAAATAGGAAAAGCTAGATGT

GAGCAACCGACTTCACCTCGATCCTTGACTCTCACTATTCACACCAGTTATGTGGGGAGCCGTAGCTCTTCCAA

TATGGCTATTGTGGAAGTGAAGATGCTATCTGGGTTCAGTCCCATGGAGGGCACCAATCAGTTACTTCTCCAG

CAACCCCTGGTGAAGAAGGTTGAATTTGGAACTGACACACTTAACATTTACTTGGATGAGCTCATTAAGAACA

CTCAGACTTACACCTTCACCATCAGCCAAAGTGTGCTGGTCACCAACTTGAAACCAGCAACCATCAAGGTCTAT

GACTACTACCTACCAGATGAACAGGCAACAATTCAGTATTCTGATCCCTGTGAATGA

>A2ML1 amino acid sequence

MWAQLLLGMLALSPAIAEELPNYLVTLPARLNFPSVQKVCLDLSPGYSDVKFTVTLETKDKTQKLLEYSGLKKRHLH

CISFLVPPPAGGTEEVATIRVSGVGNNISFEEKKKVLIQRQGNGTFVQTDKPLYTPGQQVYFRIVTMDSNFVPVNDK

YSMVELQDPNSNRIAQWLEVVPEQGIVDLSFQLAPEAMLGTYTVAVAEGKTFGTFSVEEYVLPKFKVEVVEPKELS

TVQESFLVKICCRYTYGKPMLGAVQVSVCQKANTYWYREVEREQLPDKCRNLSGQTDKTGCFSAPVDMATFDLIG

YAYSHQINIVATVVEEGTGVEANATQNIYISPQMGSMTFEDTSNFYHPNFPFSGKIRVRGHDDSFLKNHLVFLVIYG

TNGTFNQTLVTDNNGLAPFTLETSGWNGTDVSLEGKFQMEDLVYNPEQVPRYYQNAYLHLRPFYSTTRSFLGIHRL

NGPLKCGQPQEVLVDYYIDPADASPDQEISFSYYLIGKGSLVMEGQKHLNSKKKGLKASFSLSLTFTSRLAPDPSLVIY

AIFPSGGVVADKIQFSVEMCFDNQVSLGFSPSQQLPGAEVELQLQAAPGSLCALRAVDESVLLLRPDRELSNRSVYG

MFPFWYGHYPYQVAEYDQCPVSGPWDFPQPLIDPMPQGHSSQRSIIWRPSFSEGTDLFSFFRDVGLKILSNAKIKK

PVDCSHRSPEYSTAMGAGGGHPEAFESSTPLHQAEDSQVRQYFPETWLWDLFPIGNSGKEAVHVTVPDAITEWK

AMSFCTSQSRGFGLSPTVGLTAFKPFFVDLTLPYSVVRGESFRLTATIFNYLKDCIRVQTDLAKSHEYQLESWADSQT

SSCLCADDAKTHHWNITAVKLGHINFTISTKILDSNEPCGGQKGFVPQKGRSDTLIKPVLVKPEGVLVEKTHSSLLCP

KGKVASESVSLELPVDIVPDSTKAYVTVLGDIMGTALQNLDGLVQMPSGCGEQNMVLFAPIIYVLQYLEKAGLLTEE

IRSRAVGFLEIGYQKELMYKHSNGSYSAFGERDGNGNTWLTAFVTKCFGQAQKFIFIDPKNIQDALKWMAGNQLP

SGCYANVGNLLHTAMKGGVDDEVSLTAYVTAALLEMGKDVDDPMVSQGLRCLKNSATSTTNLYTQALLAYIFSLA

GEMDIRNILLKQLDQQAIISGESIYWSQKPTPSSNASPWSEPAAVDVELTAYALLAQLTKPSLTQKEIAKATSIVAWL

AKQHNAYGGFSSTQDTVVALQALAKYATTAYMPSEEINLVVKSTENFQRTFNIQSVNRLVFQQDTLPNVPGMYTL

EASGQGCVYVQTVLRYNILPPTNMKTFSLSVEIGKARCEQPTSPRSLTLTIHTSYVGSRSSSNMAIVEVKMLSGFSP

MEGTNQLLLQQPLVKKVEFGTDTLNIYLDELIKNTQTYTFTISQSVLVTNLKPATIKVYDYYLPDEQATIQYSDPCE

>A2ML1-TEV DNA sequence

ATGTGGGCTCAGCTCCTTCTAGGAATGTTGGCCCTATCACCAGCCATTGCAGAAGAACTTCCAAACTACCTGGT

GACATTACCAGCCCGGCTAAATTTCCCCTCCGTTCAGAAGGTTTGTTTGGACCTGAGCCCTGGGTACAGTGAT

GTTAAATTCACGGTTACTCTGGAGACCAAGGACAAGACCCAGAAGTTGCTAGAATACTCTGGACTGAAGAAG

AGGCACTTACATTGTATCTCCTTTCTTGTACCACCTCCTGCTGGTGGCACAGAAGAAGTGGCCACAATCCGGGT

GTCGGGAGTTGGAAATAACATCAGCTTTGAGGAGAAGAAAAAGGTTCTAATTCAGAGGCAGGGGAACGGCA

CCTTTGTACAGACTGACAAACCTCTCTACACCCCAGGGCAGCAAGTGTATTTCCGCATTGTCACCATGGATAGC

AACTTCGTTCCAGTGAATGACAAGTACTCCATGGTGGAACTACAGGATCCAAATAGCAACAGGATTGCACAGT

GGCTGGAAGTGGTACCTGAGCAAGGCATTGTAGACCTGTCCTTCCAACTGGCACCAGAGGCAATGCTGGGCA

CCTACACTGTGGCAGTGGCTGAGGGCAAGACCTTTGGTACTTTCAGTGTGGAGGAATATGTGCTGCCGAAGT

TTAAGGTGGAAGTGGTGGAACCCAAGGAGTTATCAACGGTGCAGGAATCTTTCTTAGTAAAAATTTGTTGTAG

GTACACCTATGGAAAGCCCATGCTAGGGGCAGTGCAGGTATCTGTGTGTCAGAAGGCAAATACTTACTGGTA

TCGAGAGGTGGAACGGGAACAGCTTCCTGACAAATGCAGGAACCTCTCTGGACAGACTGACAAAACAGGAT

GTTTCTCAGCACCTGTGGACATGGCCACCTTTGACCTCATTGGATATGCGTACAGCCATCAAATCAATATTGTG

GCTACTGTTGTGGAGGAAGGGACAGGTGTGGAGGCCAATGCCACTCAGAATATCTACATTTCTCCACAAATG

GGATCAATGACCTTTGAAGACACCAGCAATTTTTACCATCCAAATTTCCCCTTCAGTGGGAAGATAAGAGTTAG

GGGCCATGATGACTCCTTCCTCAAGAACCATCTAGTGTTTCTGGTGATTTATGGCACAAATGGAACCTTCAACC

AGACCCTGGTTACTGATAACAATGGCCTAGCTCCCTTTACCTTGGAGACATCCGGTTGGAATGGGACAGACGT

TTCTCTGGAGGGAAAGTTTCAAATGGAAGACTTAGTATATAATCCGGAACAAGTGCCACGTTACTACCAAAAT

GCCTACCTGCACCTGCGACCCTTCTACAGCACAACCCGCAGCTTCCTTGGCATCCACCGGCTAAACGGCCCCTT

GAAATGTGGCCAGCCCCAGGAAGTGCTGGTGGATTATTACATCGACCCGGCCGATGCAAGCCCTGACCAAGA

GATCAGCTTCTCCTACTATTTAATAGGGAAAGGAAGTTTGGTGATGGAGGGGCAGAAACACCTGAACTCTAA

GAAGAAAGGACTGAAAGCCTCCTTCTCTCTCTCACTGACCTTCACTTCGAGACTGGCCCCTGATCCTTCCCTGG

TGATCTATGCCATTTTTCCCAGTGGAGGTGTTGTAGCTGACAAAATTCAGTTCTCAGTCGAGATGTGCTTTGAC

AATCAGGTTTCCCTTGGCTTCTCCCCCTCCCAGCAGCTTCCAGGAGCAGAAGTGGAGCTGCAGCTGCAGGCAG

CTCCCGGATCCCTGTGTGCGCTCCGGGCGGTGGATGAGAGTGTCTTACTGCTTAGGCCAGACAGAGAGCTGA

GCAACCGCTCTGTCTATGGGATGTTTCCATTCTGGTATGGTCACTACCCCTATCAAGTGGCTGAGTATGATCAG

TGTCCAGTGTCTGGCCCATGGGACTTTCCTCAGCCCCTCATTGACCCAATGCCCCAAGGGCATTCGAGCCAGC

GTTCCATTATCTGGAGGCCCTCGTTCTCTGAAGGCACGGACCTTTTCAGCTTTTTCCGGGACGTGGGCCTGAAA

ATACTGTCCAATGCCAAAATCAAGAAGCCAGTAGATTGCAGTCACAGATCTCCAGAAAACCTGTATTTTCAGA

GCGCAGGCGGTGGTCATCCAGAGGCTTTTGAGTCATCAACTCCTTTACATCAAGCAGAGGATTCTCAGGTCCG

CCAGTACTTCCCAGAGACCTGGCTCTGGGATCTGTTTCCTATTGGTAACTCGGGGAAGGAGGCGGTCCACGTC

ACAGTTCCTGACGCCATCACCGAGTGGAAGGCGATGAGTTTCTGCACTTCCCAGTCAAGAGGCTTCGGGCTTT

CACCCACTGTTGGACTAACTGCTTTCAAGCCGTTCTTTGTTGACCTGACTCTCCCTTACTCAGTAGTCCGTGGGG

AATCCTTTCGTCTTACTGCCACCATCTTCAATTACCTAAAGGATTGCATCAGGGTTCAGACTGACCTGGCTAAA

TCGCATGAGTACCAGCTAGAATCATGGGCAGATTCTCAGACCTCCAGTTGTCTCTGTGCTGATGACGCAAAAA

CCCACCACTGGAACATCACAGCTGTCAAATTGGGTCACATTAACTTTACTATTAGTACAAAGATTCTGGACAGC

AATGAACCATGTGGGGGCCAGAAGGGGTTTGTTCCCCAAAAGGGCCGAAGTGACACGCTCATCAAGCCAGTT

CTCGTCAAACCTGAGGGAGTCCTGGTGGAGAAGACACACAGCTCATTGCTGTGCCCAAAAGGAAAGGTGGCA
TCTGAATCTGTCTCCCTGGAGCTCCCAGTGGACATTGTTCCTGACTCGACCAAGGCTTATGTTACGGTTCTGGG
AGACATTATGGGCACAGCCCTGCAGAACCTGGATGGTCTGGTGCAGATGCCCAGTGGCTGTGGCGAGCAGA
ACATGGTCTTGTTTGCTCCCATCATCTATGTCTTGCAGTACCTGGAGAAGGCAGGGCTGCTGACGGAGGAGAT
CAGGTCTCGGGCAGTGGGTTTCCTGGAAATAGGGTACCAGAAGGAGCTGATGTACAAACACAGCAATGGCTC
ATACAGTGCCTTTGGGGAGCGAGATGGAAATGGAAACACATGGCTGACAGCGTTTGTCACAAAATGCTTTGG
CCAAGCTCAGAAATTCATCTTCATTGATCCCAAGAACATCCAGGATGCTCTCAAGTGGATGGCAGGAAACCAG
CTCCCCAGTGGCTGCTATGCCAACGTGGGAAATCTCCTTCACACAGCTATGAAGGGTGGTGTTGATGATGAGG
TCTCCTTGACTGCGTATGTCACAGCTGCATTGCTGGAGATGGGAAAGGATGTAGATGACCCAATGGTGAGTC
AGGGTCTACGGTGTCTCAAGAATTCGGCCACCTCCACGACCAACCTCTACACACAGGCCCTGTTGGCTTACATT
TTCTCCCTGGCTGGGGAAATGGACATCAGAAACATTCTCCTTAAACAGTTAGATCAACAGGCTATCATCTCAG
GAGAATCCATTTACTGGAGCCAGAAACCTACTCCATCATCGAACGCCAGCCCTTGGTCTGAGCCTGCGGCTGT
AGATGTGGAACTCACAGCATATGCATTGTTGGCCCAGCTTACCAAGCCCAGCCTGACTCAAAAGGAGATAGC
GAAGGCCACTAGCATAGTGGCTTGGTTGGCCAAGCAACACAATGCATATGGGGGCTTCTCTTCTACTCAGGAT
ACTGTAGTTGCTCTCCAAGCTCTTGCCAAATATGCCACTACCGCCTACATGCCATCTGAGGAGATCAACCTGGT
TGTAAAATCCACTGAGAATTTCCAGCGCACATTCAACATACAGTCAGTTAACAGATTGGTATTTCAGCAGGATA
CCCTGCCCAATGTCCCTGGAATGTACACGTTGGAGGCCTCAGGCCAGGGCTGTGTCTATGTGCAGACGGTGTT
GAGATACAATATTCTCCCTCCCACAAATATGAAGACCTTTAGTCTTAGTGTGGAAATAGGAAAAGCTAGATGT
GAGCAACCGACTTCACCTCGATCCTTGACTCTCACTATTCACACCAGTTATGTGGGGAGCCGTAGCTCTTCCAA
TATGGCTATTGTGGAAGTGAAGATGCTATCTGGGTTCAGTCCCATGGAGGGCACCAATCAGTTACTTCTCCAG
CAACCCCTGGTGAAGAAGGTTGAATTTGGAACTGACACACTTAACATTTACTTGGATGAGCTCATTAAGAACA
CTCAGACTTACACCTTCACCATCAGCCAAAGTGTGCTGGTCACCAACTTGAAACCAGCAACCATCAAGGTCTAT
GACTACTACCTACCAGATGAACAGGCAACAATTCAGTATTCTGATCCCTGTGA

>A2ML1-TEV amino acid sequence

MWAQLLLGMLALSPAIAEELPNYLVTLPARLNFPSVQKVCLDLSPGYSDVKFTVTLETKDKTQKLLEYSGLKKRHLH
CISFLVPPPAGGTEEVATIRVSGVGNNISFEEKKKVLIQRQGNGTFVQTDKPLYTPGQQVYFRIVTMDSNFVPVNDK
YSMVELQDPNSNRIAQWLEVVPEQGIVDLSFQLAPEAMLGTYTVAVAEGKTFGTFSVEEYVLPKFKVEVVEPKELS
TVQESFLVKICCRYTYGKPMLGAVQVSVCQKANTYWYREVEREQLPDKCRNLSGQTDKTGCFSAPVDMATFDLIG
YAYSHQINIVATVVEEGTGVEANATQNIYISPQMGSMTFEDTSNFYHPNFPFSGKIRVRGHDDSFLKNHLVFLVIYG
TNGTFNQTLVTDNNGLAPFTLETSGWNGTDVSLEGKFQMEDLVYNPEQVPRYYQNAYLHLRPFYSTTRSFLGIHRL
NGPLKCGQPQEVLVDYYIDPADASPDQEISFSYYLIGKGSLVMEGQKHLNSKKKGLKASFSLSLTFTSRLAPDPSLVIY
AIFPSGGVVADKIQFSVEMCFDNQVSLGFSPSQQLPGAEVELQLQAAPGSLCALRAVDESVLLLRPDRELSNRSVYG
MFPFWYGHYPYQVAEYDQCPVSGPWDFPQPLIDPMPQGHSSQRSIIWRPSFSEGTDLFSFFRDVGLKILSNAKIKK
PVDCSHRSPENLYFQSAGGGHPEAFESSTPLHQAEDSQVRQYFPETWLWDLFPIGNSGKEAVHVTVPDAITEWKA

MSFCTSQSRGFGLSPTVGLTAFKPFFVDLTLPYSVVRGESFRLTATIFNYLKDCIRVQTDLAKSHEYQLESWADSQTS

SCLCADDAKTHHWNITAVKLGHINFTISTKILDSNEPCGGQKGFVPQKGRSDTLIKPVLVKPEGVLVEKTHSSLLCPK

GKVASESVSLELPVDIVPDSTKAYVTVLGDIMGTALQNLDGLVQMPSGCGEQNMVLFAPIIYVLQYLEKAGLLTEEI

RSRAVGFLEIGYQKELMYKHSNGSYSAFGERDGNGNTWLTAFVTKCFGQAQKFIFIDPKNIQDALKWMAGNQLP

SGCYANVGNLLHTAMKGGVDDEVSLTAYVTAALLEMGKDVDDPMVSQGLRCLKNSATSTTNLYTQALLAYIFSLA

GEMDIRNILLKQLDQQAIISGESIYWSQKPTPSSNASPWSEPAAVDVELTAYALLAQLTKPSLTQKEIAKATSIVAWL

AKQHNAYGGFSSTQDTVVALQALAKYATTAYMPSEEINLVVKSTENFQRTFNIQSVNRLVFQQDTLPNVPGMYTL

EASGQGCVYVQTVLRYNILPPTNMKTFSLSVEIGKARCEQPTSPRSLTLTIHTSYVGSRSSSNMAIVEVKMLSGFSP

MEGTNQLLLQQPLVKKVEFGTDTLNIYLDELIKNTQTYTFTISQSVLVTNLKPATIKVYDYYLPDEQATIQYSDPCE

>A2ML1-TEV fRBD DNA sequence

ATGTGGGCTCAGCTCCTTCTAGGAATGTTGGCCCTATCACCAGCCATTGCAGAAGAACTTCCAAACTACCTGGT

GACATTACCAGCCCGGCTAAATTTCCCCTCCGTTCAGAAGGTTTGTTTGGACCTGAGCCCTGGGTACAGTGAT

GTTAAATTCACGGTTACTCTGGAGACCAAGGACAAGACCCAGAAGTTGCTAGAATACTCTGGACTGAAGAAG

AGGCACTTACATTGTATCTCCTTTCTTGTACCACCTCCTGCTGGTGGCACAGAAGAAGTGGCCACAATCCGGGT

GTCGGGAGTTGGAAATAACATCAGCTTTGAGGAGAAGAAAAAGGTTCTAATTCAGAGGCAGGGGAACGGCA

CCTTTGTACAGACTGACAAACCTCTCTACACCCCAGGGCAGCAAGTGTATTTCCGCATTGTCACCATGGATAGC

AACTTCGTTCCAGTGAATGACAAGTACTCCATGGTGGAACTACAGGATCCAAATAGCAACAGGATTGCACAGT

GGCTGGAAGTGGTACCTGAGCAAGGCATTGTAGACCTGTCCTTCCAACTGGCACCAGAGGCAATGCTGGGCA

CCTACACTGTGGCAGTGGCTGAGGGCAAGACCTTTGGTACTTTCAGTGTGGAGGAATATGTGCTGCCGAAGT

TTAAGGTGGAAGTGGTGGAACCCAAGGAGTTATCAACGGTGCAGGAATCTTTCTTAGTAAAAATTTGTTGTAG

GTACACCTATGGAAAGCCCATGCTAGGGGCAGTGCAGGTATCTGTGTGTCAGAAGGCAAATACTTACTGGTA

TCGAGAGGTGGAACGGGAACAGCTTCCTGACAAATGCAGGAACCTCTCTGGACAGACTGACAAAACAGGAT

GTTTCTCAGCACCTGTGGACATGGCCACCTTTGACCTCATTGGATATGCGTACAGCCATCAAATCAATATTGTG

GCTACTGTTGTGGAGGAAGGGACAGGTGTGGAGGCCAATGCCACTCAGAATATCTACATTTCTCCACAAATG

GGATCAATGACCTTTGAAGACACCAGCAATTTTTACCATCCAAATTTCCCCTTCAGTGGGAAGATAAGAGTTAG

GGGCCATGATGACTCCTTCCTCAAGAACCATCTAGTGTTTCTGGTGATTTATGGCACAAATGGAACCTTCAACC

AGACCCTGGTTACTGATAACAATGGCCTAGCTCCCTTTACCTTGGAGACATCCGGTTGGAATGGGACAGACGT

TTCTCTGGAGGGAAAGTTTCAAATGGAAGACTTAGTATATAATCCGGAACAAGTGCCACGTTACTACCAAAAT

GCCTACCTGCACCTGCGACCCTTCTACAGCACAACCCGCAGCTTCCTTGGCATCCACCGGCTAAACGGCCCCTT

GAAATGTGGCCAGCCCCAGGAAGTGCTGGTGGATTATTACATCGACCCGGCCGATGCAAGCCCTGACCAAGA

GATCAGCTTCTCCTACTATTTAATAGGGAAAGGAAGTTTGGTGATGGAGGGGCAGAAACACCTGAACTCTAA

GAAGAAAGGACTGAAAGCCTCCTTCTCTCTCTCACTGACCTTCACTTCGAGACTGGCCCCTGATCCTTCCCTGG

TGATCTATGCCATTTTTCCCAGTGGAGGTGTTGTAGCTGACAAAATTCAGTTCTCAGTCGAGATGTGCTTTGAC
AATCAGGTTTCCCTTGGCTTCTCCCCCTCCCAGCAGCTTCCAGGAGCAGAAGTGGAGCTGCAGCTGCAGGCAG
CTCCCGGATCCCTGTGTGCGCTCCGGGCGGTGGATGAGAGTGTCTTACTGCTTAGGCCAGACAGAGAGCTGA
GCAACCGCTCTGTCTATGGGATGTTTCCATTCTGGTATGGTCACTACCCCTATCAAGTGGCTGAGTATGATCAG
TGTCCAGTGTCTGGCCCATGGGACTTTCCTCAGCCCCTCATTGACCCAATGCCCCAAGGGCATTCGAGCCAGC
GTTCCATTATCTGGAGGCCCTCGTTCTCTGAAGGCACGGACCTTTTCAGCTTTTTCCGGGACGTGGGCCTGAAA
ATACTGTCCAATGCCAAAATCAAGAAGCCAGTAGATTGCAGTCACAGATCTCCAGAAAACCTGTATTTTCAGA
GCGCAGGCGGTGGTCATCCAGAGGCTTTTGAGTCATCAACTCCTTTACATCAAGCAGAGGATTCTCAGGTCCG
CCAGTACTTCCCAGAGACCTGGCTCTGGGATCTGTTTCCTATTGGTAACTCGGGGAAGGAGGCGGTCCACGTC
ACAGTTCCTGACGCCATCACCGAGTGGAAGGCGATGAGTTTCTGCACTTCCCAGTCAAGAGGCTTCGGGCTTT
CACCCACTGTTGGACTAACTGCTTTCAAGCCGTTCTTTGTTGACCTGACTCTCCCTTACTCAGTAGTCCGTGGGG
AATCCTTTCGTCTTACTGCCACCATCTTCAATTACCTAAAGGATTGCATCAGGGTTCAGACTGACCTGGCTAAA
TCGCATGAGTACCAGCTAGAATCATGGGCAGATTCTCAGACCTCCAGTTGTCTCTGTGCTGATGACGCAAAAA
CCCACCACTGGAACATCACAGCTGTCAAATTGGGTCACATTAACTTTACTATTAGTACAAAGATTCTGGACAGC
AATGAACCATGTGGGGGCCAGAAGGGGTTTGTTCCCCAAAAGGGCCGAAGTGACACGCTCATCAAGCCAGTT
CTCGTCAAACCTGAGGGAGTCCTGGTGGAGAAGACACACAGCTCATTGCTGTGCCCAAAAGGAAAGGTGGCA
TCTGAATCTGTCTCCCTGGAGCTCCCAGTGGACATTGTTCCTGACTCGACCAAGGCTTATGTTACGGTTCTGGG
AGACATTATGGGCACAGCCCTGCAGAACCTGGATGGTCTGGTGCAGATGCCCAGTGGCTGTGGCGAGCAGA
ACATGGTCTTGTTTGCTCCCATCATCTATGTCTTGCAGTACCTGGAGAAGGCAGGGCTGCTGACGGAGGAGAT
CAGGTCTCGGGCAGTGGGTTTCCTGGAAATAGGGTACCAGAAGGAGCTGATGTACAAACACAGCAATGGCTC
ATACAGTGCCTTTGGGGAGCGAGATGGAAATGGAAACACATGGCTGACAGCGTTTGTCACAAAATGCTTTGG
CCAAGCTCAGAAATTCATCTTCATTGATCCCAAGAACATCCAGGATGCTCTCAAGTGGATGGCAGGAAACCAG
CTCCCCAGTGGCTGCTATGCCAACGTGGGAAATCTCCTTCACACAGCTATGAAGGGTGGTGTTGATGATGAGG
TCTCCTTGACTGCGTATGTCACAGCTGCATTGCTGGAGATGGGAAAGGATGTAGATGACCCAATGGTGAGTC
AGGGTCTACGGTGTCTCAAGAATTCGGCCACCTCCACGACCAACCTCTACACACAGGCCCTGTTGGCTTACATT
TTCTCCCTGGCTGGGGAAATGGACATCAGAAACATTCTCCTTAAACAGTTAGATCAACAGGCTATCATCTCAG
GAGAATCCATTTACTGGAGCCAGAAACCTACTCCATCATCGAACGCCAGCCCTTGGTCTGAGCCTGCGGCTGT
AGATGTGGAACTCACAGCATATGCATTGTTGGCCCAGCTTACCAAGCCCAGCCTGACTCAAAAGGAGATAGC
GAAGGCCACTAGCATAGTGGCTTGGTTGGCCAAGCAACACAATGCATATGGGGGCTTCTCTTCTACTCAGGAT
ACTGTAGTTGCTCTCCAAGCTCTTGCCAAATATGCCACTACCGCCTACATGCCATCTGAGGAGATCAACCTGGT
TGTAAAATCCACTGAGAATTTCCAGCGCACATTCAACATACAGTCAGTTAACAGATTGGTATTTCAGCAGGATA
CCCTGCCCAATGTCCCTGGAATGTACACGTTGGAGGCCTCAGGCCAGGGCTGTGTCTATGTGCAGACGGTGTT
GAGATACAATATTCTCCCTCCCTCTGGGTCTTCCAGAAGACGAAGATCTACAAATATGAAGACCTTTAGTCTTA
GTGTGGAAATAGGAAAAGCTAGATGTGAGCAACCGACTTCACCTCGATCCTTGACTCTCACTATTCACACCAG

TTATGTGGGGAGCCGTAGCTCTTCCAATATGGCTATTGTGGAAGTGAAGATGCTATCTGGGTTCAGTCCCATG
GAGGGCACCAATCAGTTACTTCTCCAGCAACCCCTGGTGAAGAAGGTTGAATTTGGAACTGACACACTTAACA
TTTACTTGGATGAGCTCATTAAGAACACTCAGACTTACACCTTCACCATCAGCCAAAGTGTGCTGGTCACCAAC
TTGAAACCAGCAACCATCAAGGTCTATGACTACTACCTACCAGATGAACAGGCAACAATTCAGTATTCTGATCC
CTGTGA

>A2ML1-TEV fRBD amino acid sequence

MWAQLLLGMLALSPAIAEELPNYLVTLPARLNFPSVQKVCLDLSPGYSDVKFTVTLETKDKTQKLLEYSGLKKRHLH
CISFLVPPPAGGTEEVATIRVSGVGNNISFEEKKKVLIQRQGNGTFVQTDKPLYTPGQQVYFRIVTMDSNFVPVNDK
YSMVELQDPNSNRIAQWLEVVPEQGIVDLSFQLAPEAMLGTYTVAVAEGKTFGTFSVEEYVLPKFKVEVVEPKELS
TVQESFLVKICCRYTYGKPMLGAVQVSVCQKANTYWYREVEREQLPDKCRNLSGQTDKTGCFSAPVDMATFDLIG
YAYSHQINIVATVVEEGTGVEANATQNIYISPQMGSMTFEDTSNFYHPNFPFSGKIRVRGHDDSFLKNHLVFLVIYG
TNGTFNQTLVTDNNGLAPFTLETSGWNGTDVSLEGKFQMEDLVYNPEQVPRYYQNAYLHLRPFYSTTRSFLGIHRL
NGPLKCGQPQEVLVDYYIDPADASPDQEISFSYYLIGKGSLVMEGQKHLNSKKKGLKASFSLSLTFTSRLAPDPSLVIY
AIFPSGGVVADKIQFSVEMCFDNQVSLGFSPSQQLPGAEVELQLQAAPGSLCALRAVDESVLLLRPDRELSNRSVYG
MFPFWYGHYPYQVAEYDQCPVSGPWDFPQPLIDPMPQGHSSQRSIIWRPSFSEGTDLFSFFRDVGLKILSNAKIKK
PVDCSHRSPENLYFQSAGGGHPEAFESSTPLHQAEDSQVRQYFPETWLWDLFPIGNSGKEAVHVTVPDAITEWKA
MSFCTSQSRGFGLSPTVGLTAFKPFFVDLTLPYSVVRGESFRLTATIFNYLKDCIRVQTDLAKSHEYQLESWADSQTS
SCLCADDAKTHHWNITAVKLGHINFTISTKILDSNEPCGGQKGFVPQKGRSDTLIKPVLVKPEGVLVEKTHSSLLCPK
GKVASESVSLELPVDIVPDSTKAYVTVLGDIMGTALQNLDGLVQMPSGCGEQNMVLFAPIIYVLQYLEKAGLLTEEI
RSRAVGFLEIGYQKELMYKHSNGSYSAFGERDGNGNTWLTAFVTKCFGQAQKFIFIDPKNIQDALKWMAGNQLP
SGCYANVGNLLHTAMKGGVDDEVSLTAYVTAALLEMGKDVDDPMVSQGLRCLKNSATSTTNLYTQALLAYIFSLA
GEMDIRNILLKQLDQQAIISGESIYWSQKPTPSSNASPWSEPAAVDVELTAYALLAQLTKPSLTQKEIAKATSIVAWL
AKQHNAYGGFSSTQDTVVALQALAKYATTAYMPSEEINLVVKSTENFQRTFNIQSVNRLVFQQDTLPNVPGMYTL
EASGQGCVYVQTVLRYNILPPSGSSRRRRSTNMKTFSLSVEIGKARCEQPTSPRSLTLTIHTSYVGSRSSSNMAIVEV
KMLSGFSPMEGTNQLLLQQPLVKKVEFGTDTLNIYLDELIKNTQTYTFTISQSVLVTNLKPATIKVYDYYLPDEQATIQ
YSDPCE

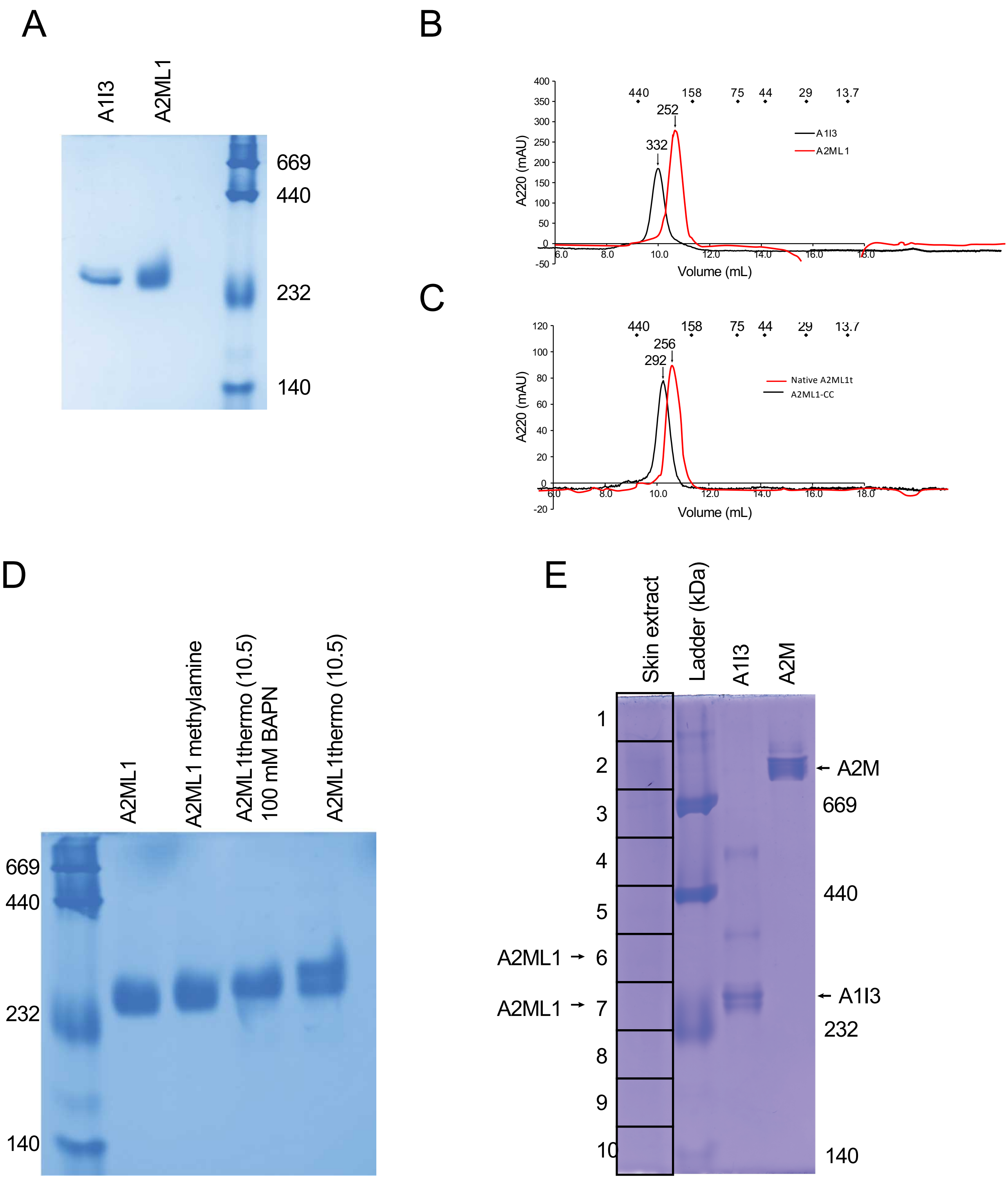

A
A1I3
A2ML1
669
440
232
140
B
A220 (mAU)
Volume (mL)
440
158
75
44
29
13.7
252
332
A1I3
A2ML1
C
A220 (mAU)
Volume (mL)
256
292
Native A2ML1t
A2ML1-CC
D
A2ML1
A2ML1 methylamine
A2ML1thermo (10.5) 100 mM BAPN
A2ML1thermo (10.5)
669
440
232
140
E
Skin extract
Ladder (kDa)
A1I3
A2M
A2M
A1I3
A2ML1
A2ML1
669
440
232
140

Supplementary Figure 1. A2ML1 is a monomer in skin. A) Native pore-limited PAGE of rat A1I3 and human A2ML1, which both migrate as monomers. This experiment was performed once. Molecular weight markers are shown on the right-hand side in kDa. B) SEC of A1I3 and A2ML1 with size markers from 13.7 kDa to 440 kDa, which further indicates that A2ML1 is a monomer in solution. C) SEC of native A2ML1 and TEV-P-cleaved A2ML1 confirms that protease-cleaved A2ML1 is a monomer in solution. D) Native pore-limited PAGE of native A2ML1, methylamine (MA) treated A2ML1, and thermolysin-cleaved A2ML1 with and without BAPN. MA-treated and protease-cleaved A2ML1 migrate as a monomer, however with a slight difference in migration compared to native A2ML1, indicating that A2ML1 undergoes a conformational change after either form of activation. These results are representative of duplicate experiments. Molecular weight markers are shown in the left-hand side in kDa. E) Native pore-limited PAGE of a skin protein extract, rat plasma-purified A1I3, and human plasma-purified A2M. A2ML1 has been identified in gel slice 6 and 7 with LC-MS/MS corresponding to the migration of a monomer. This experiment was performed once. Molecular weight markers are shown in the right-hand side in kDa.

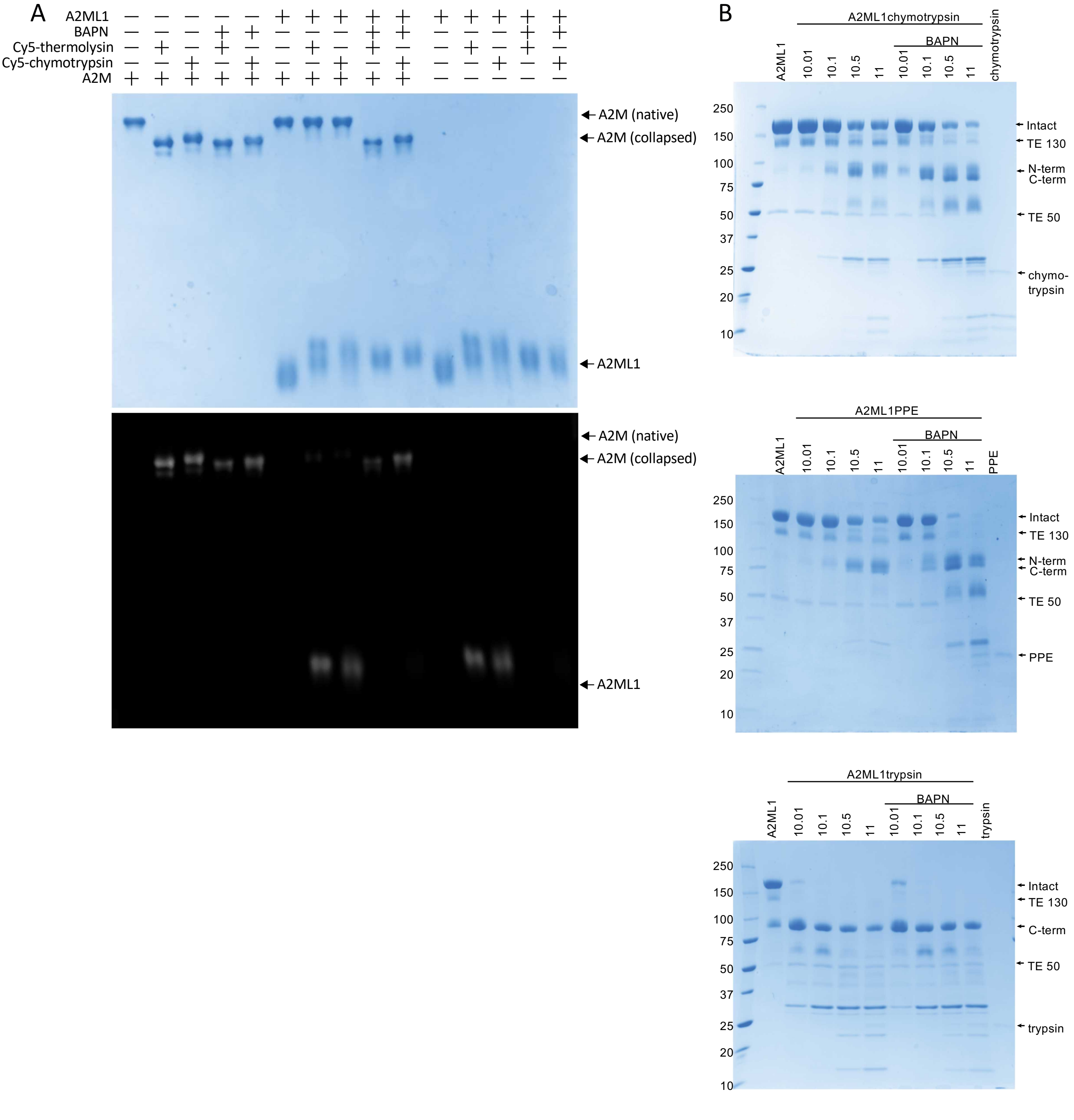
A
A2ML1
BAPN
Cy5-thermolysin
Cy5-chymotrypsin
A2M
A2M (native)
A2M (collapsed)
A2ML1
B
A2ML1chymotrypsin
BAPN
A2ML1
10.01
10.1
10.5
11
chymotrypsin
250
150
100
75
50
37
25
20
10
Intact
TE 130
N-term
C-term
TE 50
chymo-
trypsin
A2ML1PPE
PPE
A2ML1trypsin
trypsin

Supplementary Figure 2. Bait region cleavage of A2ML1 and resultant covalent protease binding. (A) A2ML1 was cleaved by Cy5-labelled chymotrypsin or thermolysin with a 4:1 molar ratio of A2ML1 to protease, with or without 200 mM BAPN. The 720 kDa A2M was then added to a molar ratio of 1:2 A2M per protease. Control samples without A2ML1 or A2M but the same amount of protease was also included. Samples were analyzed by native pore limited PAGE. Coomassie-stained (above) and Cy5 fluorescence (below) images are shown. A2ML1 is able to prevent chymotrypsin and thermolysin from accessing and cleaving A2M, but only if A2ML1 able to covalently bind to proteases (i.e. BAPN is not included). In the absence of BAPN, proteases co-migrate with A2ML1 in native PAGE. This experiment was performed once. (B) A2ML1 was cleaved by the indicated molar ratios of chymotrypsin, pig pancreatic elastase (PPE), or trypsin, with or without 50 mM BAPN as indicated. Bait region cleavage results in the formation of two ≈90 kDa N-terminal and C-terminal product bands, as are apparent after cleavage by chymotrypsin and PPE but not trypsin. BAPN prevents covalent protease conjugation and therefore prevents protease inhibition by A2ML1; A2ML1 is cleaved to a greater extent by chymotrypsin and PPE in the presence of BAPN, indicating that these proteases are inhibited by A2ML1 without BAPN. These results are representative of duplicate experiments. Molecular weight markers are shown in the left-hand side in kDa.

A

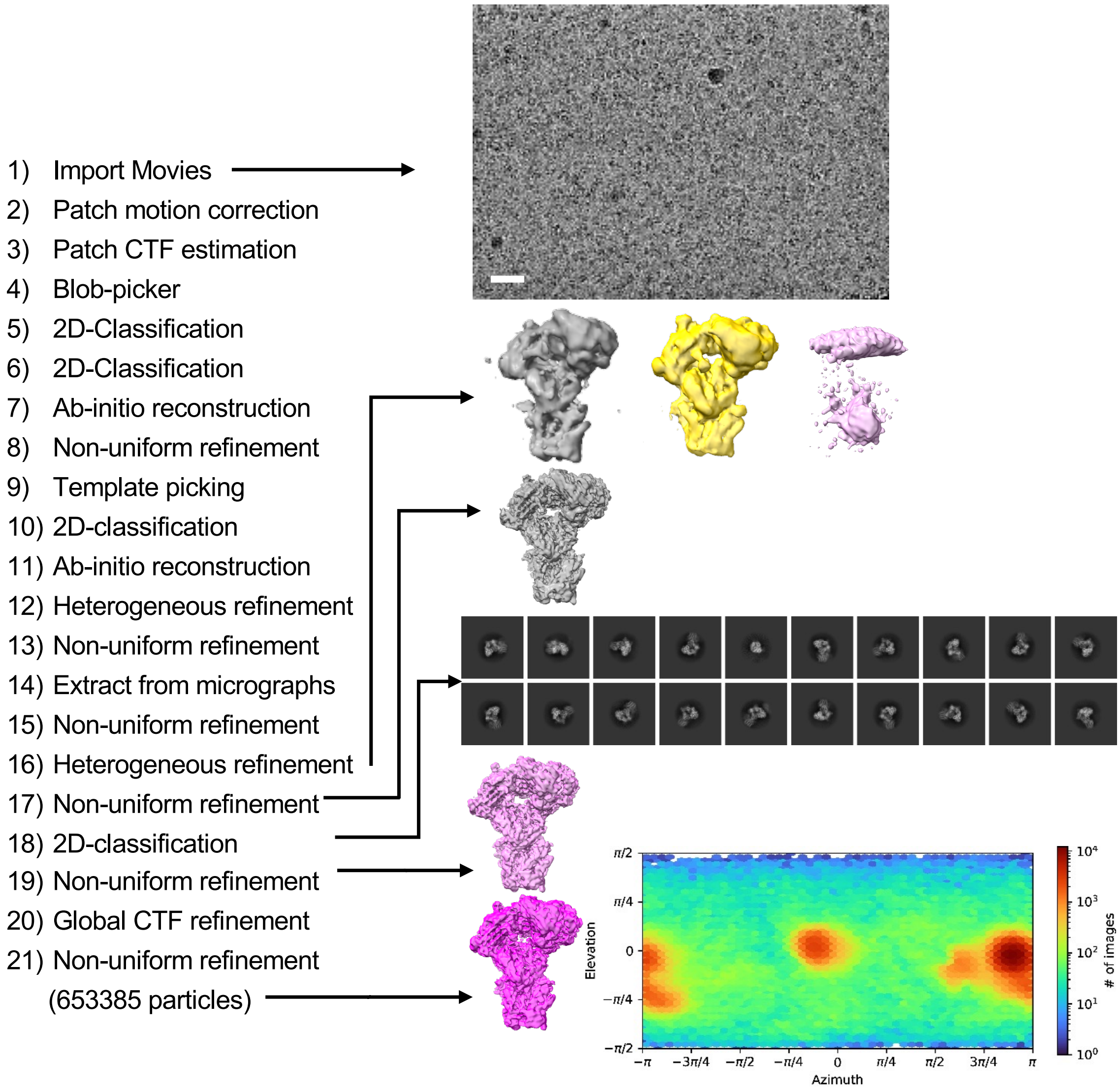

B

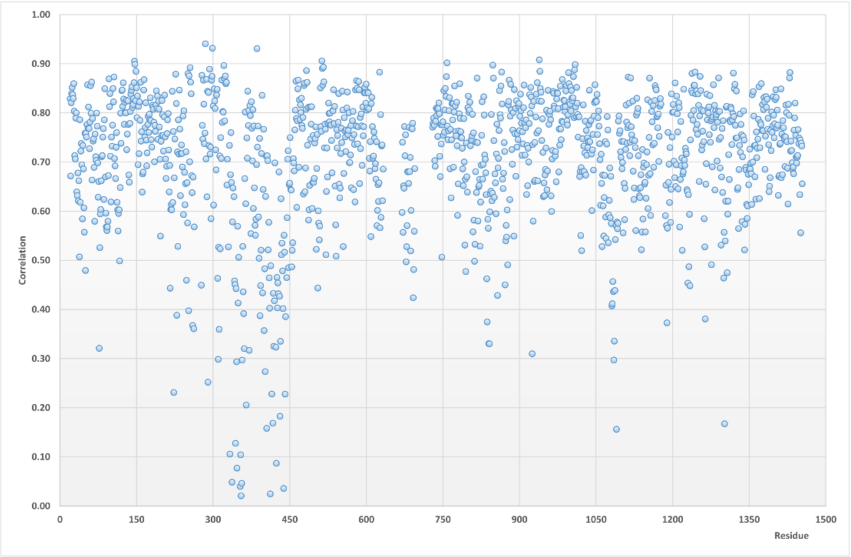

Domain	Correlation
MG1	0.71
MG2	0.78
MG3	0.68
MG4	0.47
MG5	0.73
MG6	0.77
LNK	0.70
BR	0.75
MG7	0.70
CUB	0.73
TE	0.72
MG8	0.73

Supplementary Figure 3. Cryo-EM data analysis leading to the 3D volume of native A2ML1. A) Flow of data processing conducted in cryosparc with examples of the 3D volumes from the associated step. For the final step, the viewing direction distribution is presented. The scale bar on the displayed micrograph is 30 nm long. In the selected 2D classification output, the 20 highest populated classes are displayed. The map obtained in the final non-uniform refinement has a resolution of 2.9 Å according to the Fourier correlation plot presented in figure 2B. In contrast to the three samples of activated A2ML1, 2D classes and 3D volumes suggesting dimers of native A2ML1 was not observed. B) Correlation between map and model calculated by residue in phenix.validation_cryoem. To the right are listed the correlations for the individual domains illustrating the low quality of the map in particular for the MG4 domain.

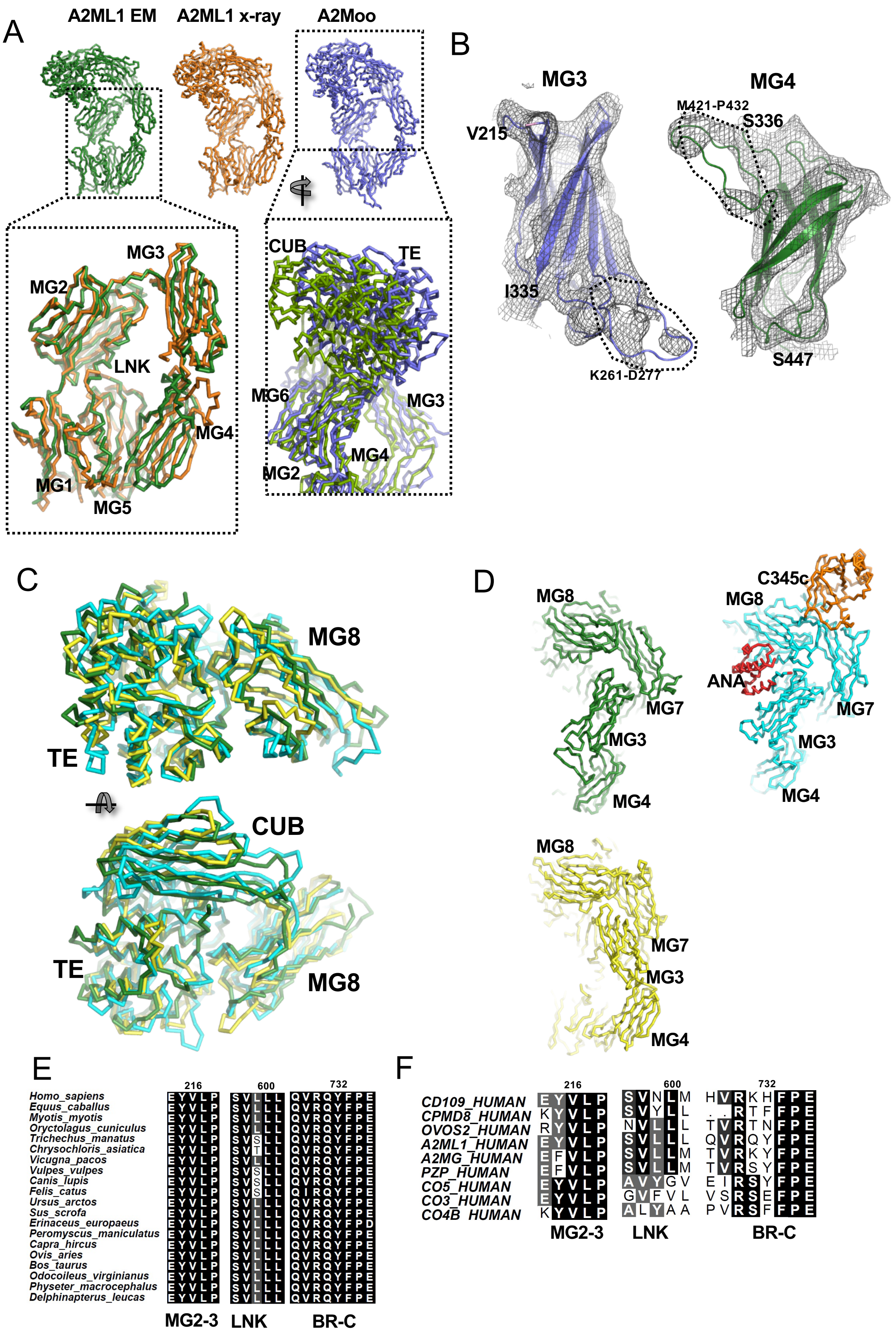
A
A2ML1 EM
A2ML1 x-ray
A2Moo
MG3
MG2
LNK
MG4
MG1
MG5
CUB
TE
MG6
MG3
MG4
MG2
B
MG3
MG4
V215
I335
K261-D277
M421-P432
S336
S447
C
MG8
TE
CUB
TE
MG8
D
MG8
MG7
MG3
MG4
C345c
MG8
ANA
MG7
MG3
MG4
MG8
MG7
MG3
MG4
E
Homo_sapiens
Equus_caballus
Myotis_myotis
Oryctolagus_cuniculus
Trichechus_manatus
Chrysochloris_asiatica
Vicugna_pacos
Vulpes_vulpes
Canis_lupis
Felis_catus
Ursus_arctos
Sus_scrofa
Erinaceus_europaeus
Peromyscus_maniculatus
Capra_hircus
Ovis_aries
Bos_taurus
Odocoileus_virginianus
Physeter_macrocephalus
Delphinapterus_leucas
216
600
732
MG2-3
LNK
BR-C
F
CD109_HUMAN
CPMD8_HUMAN
OVOS2_HUMAN
A2ML1_HUMAN
A2MG_HUMAN
PZP_HUMAN
CO5_HUMAN
CO3_HUMAN
CO4B_HUMAN
216
600
732
MG2-3
LNK
BR-C

Supplementary Figure 4. Structural details of the native A2ML1. A) Comparison of the overall conformation of native A2ML1 EM structure (green), A2ML1 X-ray structure (orange) and the native A2Moo subunit (blue). In the magnified view to the left, a significant difference in the orientations of the MG3 and MG4 domains in the two different structures of native A2ML1 is observed. In the magnified view to the right (rotated 90° relative to top row), a 19° difference between A2ML1 and A2M in the rotation of the CTM8 relative to the MG2 and MG6 domains is apparent. B) Left, final $2mF_o$-DF_c crystallographic map contoured around the A2ML1 MG3 domain at 0.8 σ contour level. Right, $2mF_o$-DF_c map contoured around the MG4 domain at 0.7 σ. The dashed lines feature apparently flexible loops protruding from these domain. C) Superposition of the CUB, TE and MG8 domains of non-activated A2ML1 (green), complement C3 (light blue), and TEP1r (yellow). The three domains pack together in the CTM8 unit in a highly conserved manner. D) Comparison of the MG3-MG7 interactions in A2ML1 (top left), C3 (top right) and TEP1r (bottom) as in panel C. Notice the very tight interaction of MG3 and MG7 in TEP1r compared to A2ML1 and C3. In complement C3, the ANA and C345c domains not present in A2ML1 are featured. E) Details of a multiple sequence alignment of mammalian A2ML1 sequences demonstrating that regions forming the plug-channel interaction in Figure 2H are very highly conserved. F) As in panel E, but with all sequences for the human A2MF proteins suggesting that the plug-channel arrangement in A2ML1 is conserved in all A2MF members regardless of function. The full alignments for mammalian A2ML1 and human A2MF proteins are provided in Supplementary Figures 11 and 12, respectively.

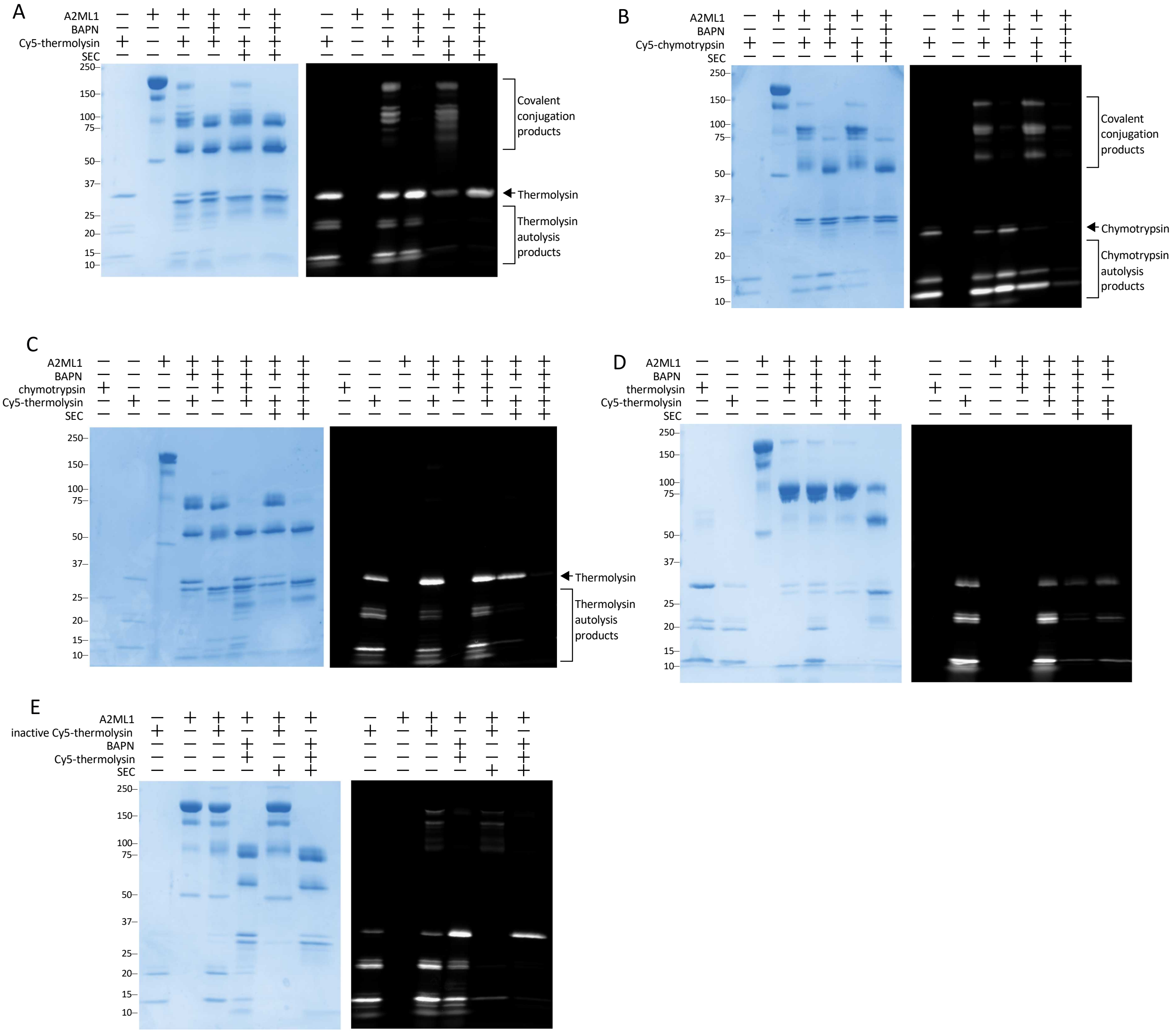

Supplementary Figure 5. Investigating non-covalent association of proteases with A2ML1. A) A2ML1 was cleaved by Cy5-labelled thermolysin at a 0.7:1 molar ratio of protease:A2ML, with or without 200 mM BAPN. The samples were then separated by SEC on a Superdex 200 Increase. The samples before SEC and the A2ML1-containing SEC fractions were then analyzed by reducing SDS-PAGE. Coomassie-stained and Cy5 fluorescence images are shown. Thermolysin co-elutes with A2ML1 in SEC, both with and without covalent conjugation (i.e. without or with the inclusion of BAPN during A2ML1's cleavage). In this experiment and in all subsequent panels, thermolysin was inactivated by 25 mM EDTA prior to SEC. B) As in panel A, but with Cy5-labelled chymotrypsin instead of thermolysin. Chymotrypsin co-elutes with A2ML1 in SEC only if it is covalently conjugated.

In this experiment and in all subsequent panels, chymotrypsin was inactivated with 2 mM PMSF prior to SEC. C) With 200 mM of BAPN present, A2ML1 was cleaved by Cy5-labelled thermolysin at a 0.7:1 molar ratio, with and without pre-cleavage using 0.1:1 molar ratio of non-labelled chymotrypsin. Samples were run on SEC. If A2ML1 is pre-cleaved with chymotrypsin, thermolysin does not co-elute with A2ML1 during SEC. D) As in panel C, but with pre-cleavage using non-labelled thermolysin instead of chymotrypsin. If A2ML1 is pre-cleaved by thermolysin, it can still co-elute with Cy5-labelled thermolysin, showing that the non-covalent association does not arise as a direct consequence of bait region cleavage and A2ML1's protease-trapping conformational change. The failure of pre-cleavage with chymotrypsin to abrogate non-covalent thermolysin conjugation, as well as the failure of chymotrypsin to non-covalently associate with A2ML1, may be due to the tendency of chymotrypsin to cleave A2ML1 extensively outside of its bait region. E) A2ML1 was incubated with inactivated Cy5-labelled thermolysin (inactivated by 25 mM EDTA) without BAPN, or active Cy5-labelled thermolysin with 200 mM BAPN, and then run on SEC. Inactive thermolysin did not co-elute with (uncleaved) A2ML1, indicating that the non-covalent association with thermolysin is specific to the cleaved conformation of A2ML1. A-E) These experiments were performed once. Molecular weight markers are shown on the left-hand side in kDa in all panels.

A

1. Import Movies
2. Patch Motion correction
3. Patch CTF estimate
4. Blob picker
5. 2D-classification
6. 2D-classification
7. Ab initio reconstruction
8. Heterogenous refinement
9. Non-uniform refinement
10. Create templates
11. Template picking
12. 2D-classification
13. 2D-classification
14. Ab initio reconstruction (M)
15. Ab initio reconstruction (D)
16. Heterogenous refinement (M+D)
17. Extract from micrographs (M)
18. Non-uniform refinement (M)
19. 2D-classification (M)
20. Non-uniform refinement (M)
21. Heterogenous refinement (M)
22. Non-uniform refinement (M)
23. Non-uniform refinement (M)
24. Local refinement (M)

(M 228619 particles)

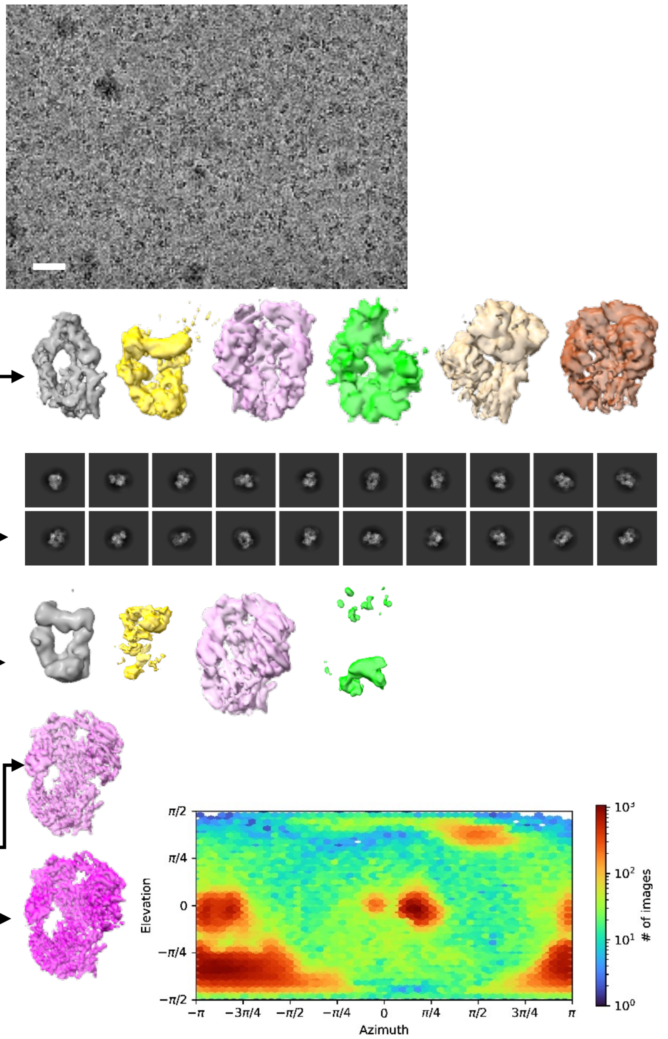

B

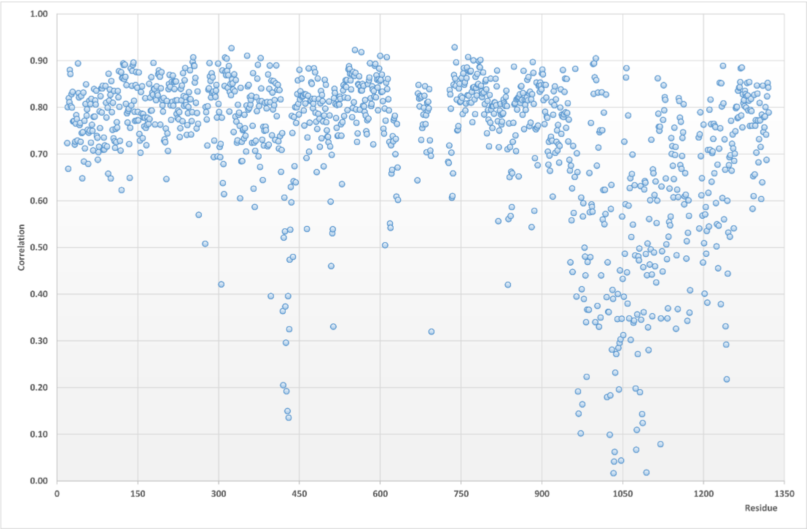

Domain	Correlation
MG1	0.78
MG2	0.81
MG3	0.80
MG4	0.71
MG5	0.79
MG6	0.83
LNK	0.77
BR	0.68
MG7	0.79
CUB	0.77
TE	0.53

Supplementary Figure 6. Cryo-EM data analysis leading to the 3D volume of the A2ML1-CE monomer. A) Flow of data processing conducted in cryosparc with examples of the 3D volumes from the associated step. The scale bar on the displayed micrograph is 30 nm long. For the final step, the viewing direction distribution is presented. In the selected 2D classification output, the 20 highest populated classes are displayed. The map obtained in the final non-uniform refinement has a resolution of 3.0 Å according to the Fourier correlation plot presented in figure 3D. In the later steps, the inclusion of monomer (M) and dimer (D) in the processing step is shown. B) Correlation between map and model calculated by residue in phenix.validation_cryoem for the two structures. Correlation is generally lower in the TE domain compared to the remaining molecule.

A

1. Import Movies
2. Patch motion correction
3. Patch CTF estimation
4. Blob-picker
5. 2D-Classicfication
6. Ab-initio reconstruction
7. Heterogenous refinement
8. Heterogenous refinement
9. Ab-initio reconstruction
10. Heterogenous refinement (M)
11. Ab-initio reconstruction (D)
12. Heterogenous refinement (D)
13. Ab-initio reconstruction (M)
14. Heterogenous refinement (M)
15. 2D-Classicfication (M)
16. Non-uniform refinement (M)
17. 2D-Classicfication (D)
18. Heterogenous refinement (M+D)
19. Non-uniform refinement
    (M 463317 particles)

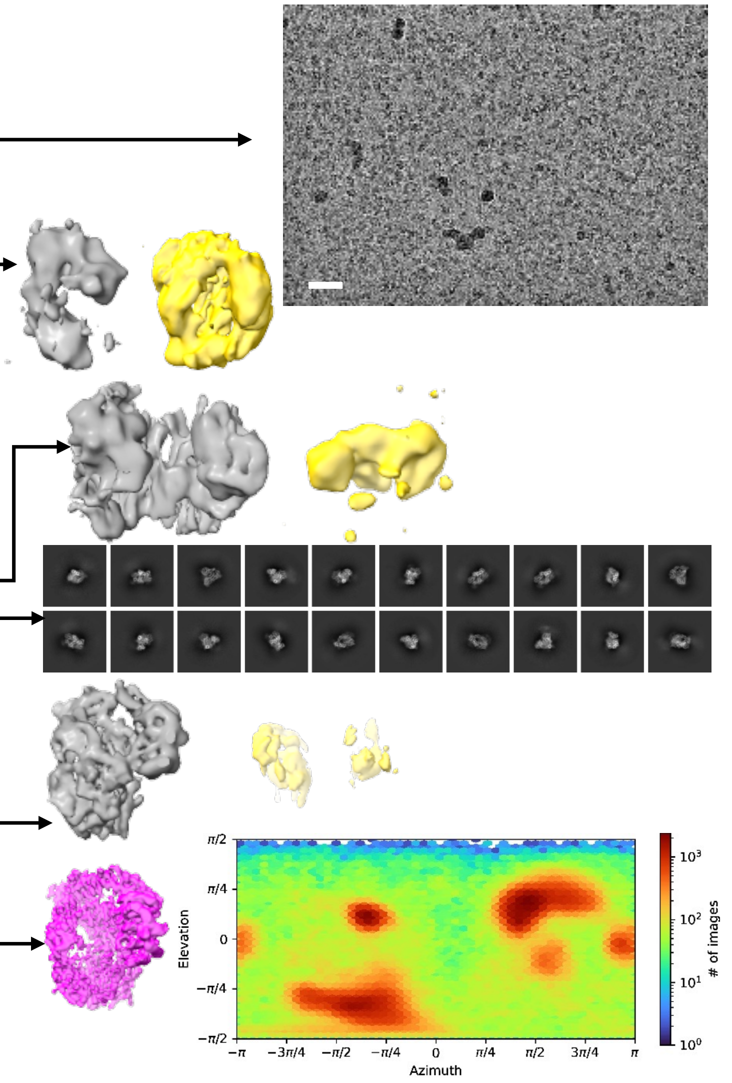

B

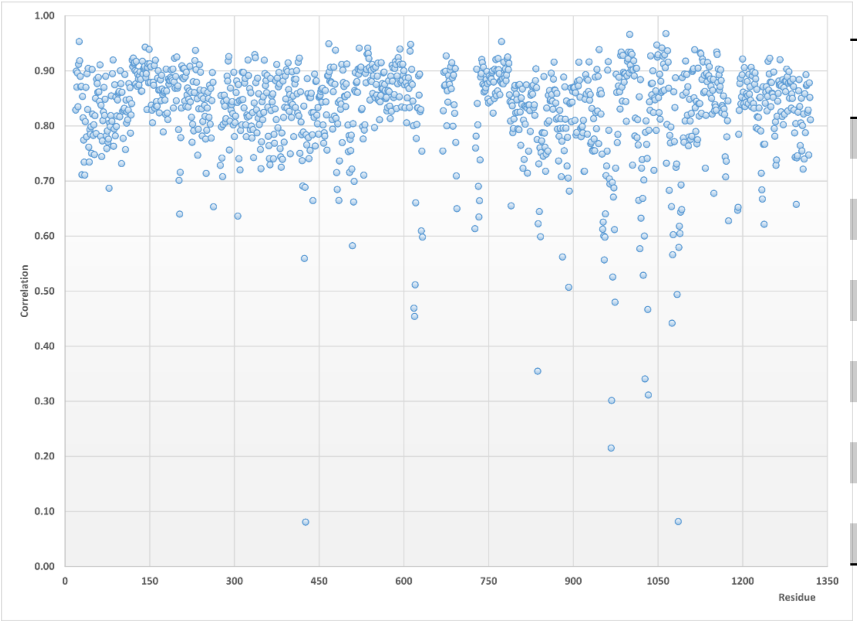

Domain	Correlation
MG1	0.83
MG2	0.87
MG3	0.83
MG4	0.82
MG5	0.84
MG6	0.88
LNK	0.83
BR	0.74
MG7	0.80
CUB	0.83
TE	0.80

Supplementary Figure 7. Cryo-EM data analysis leading to the 3D volume of the A2ML1-CC monomer. A) Flow of data processing conducted in cryosparc with examples of the 3D volumes from the associated step. The scale bar on the displayed micrograph is 30 nm long. For the final step, the viewing direction distribution is presented. In the selected 2D classification output, the 20 highest populated classes are displayed. The map obtained in the final non-uniform refinement has a resolution of 2.9 Å according to the Fourier correlation plot presented in figure 3D. In the later steps, the inclusion of monomer (M) and dimer (D) in the processing step is shown. B) Correlation between map and model calculated by residue in phenix.validation_cryoem.

A

1. Import Movies
2. Patch Motion correction
3. Patch CTF estimation
4. Blob-picker
5. 2D-classification
6. Ab initio reconstruction
7. Heterogenous refinement
8. Template picker
9. Heterogenous refinement
10. 2D-classification (D)
11. 2D-classification (M)
12. Non-uniform refinement (D)
13. Non-uniform refinement (M)
14. Heterogenous refinement (M+D)
15. Non-uniform refinement (D 174680 particles)

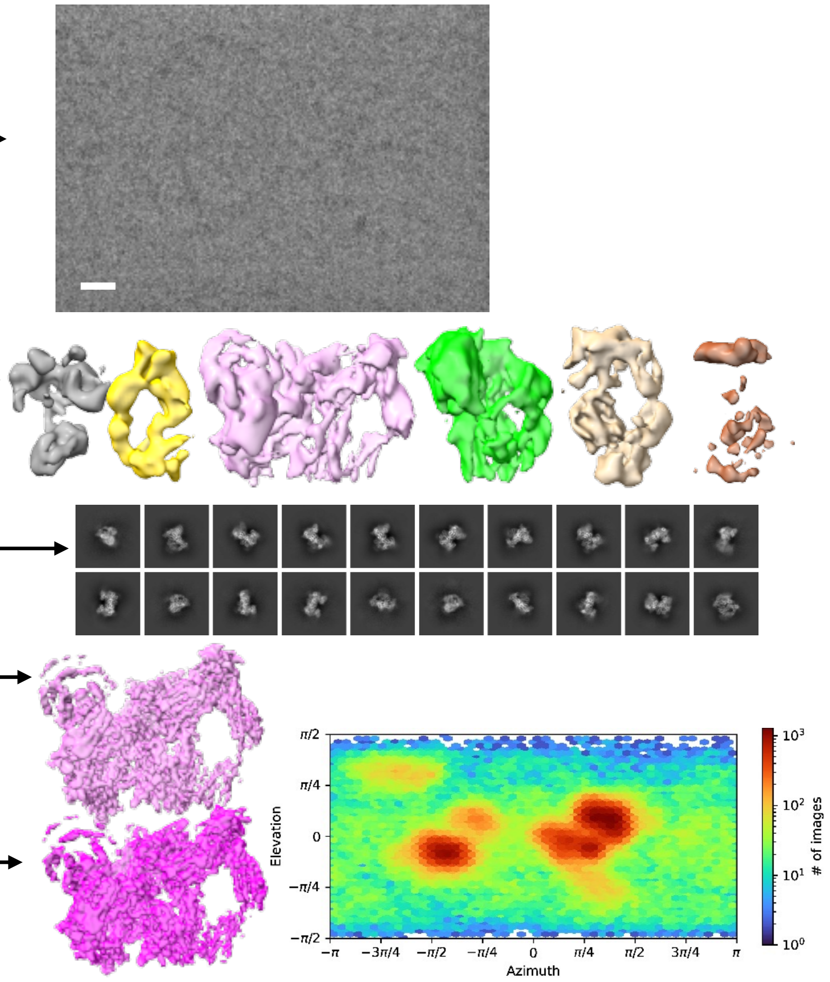

B

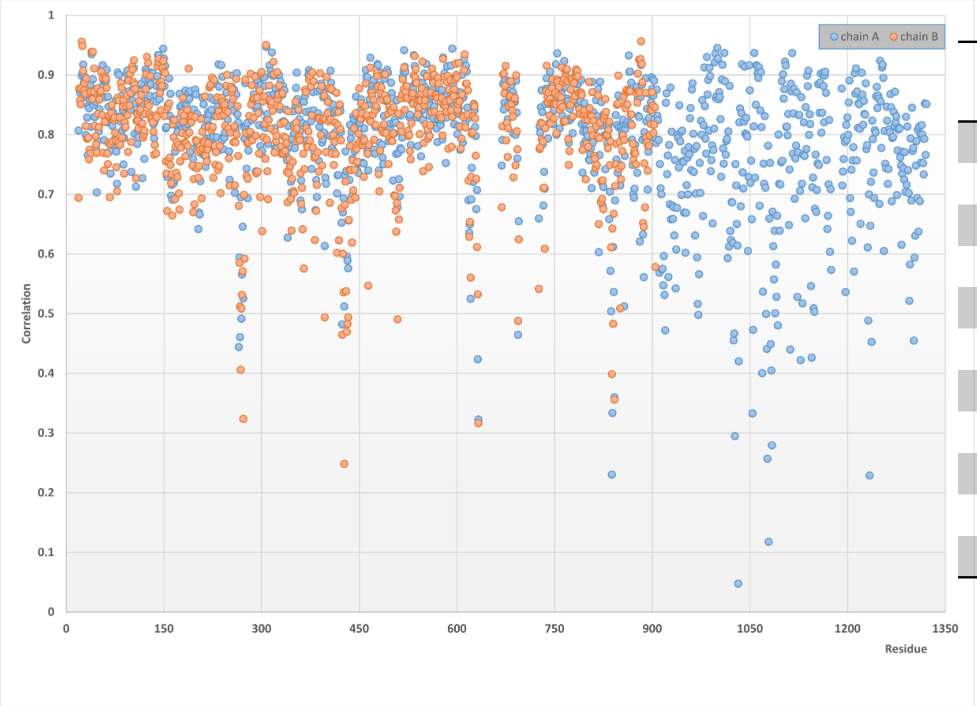

Domain	Correlation
MG1	0.84/0.84
MG2	0.83/0.80
MG3	0.82/0.80
MG4	0.79/0.75
MG5	0.84/0.82
MG6	0.86/0.86
LNK	0.80/0.80
BR	0.77/0.74
MG7	0.78/0.79
CUB	0.74/ NA
TE	0.75/ NA

Supplementary Figure 8. Cryo-EM data analysis leading to the 3D volume of the A2ML1-CA dimer. A) Flow of data processing conducted in cryosparc with examples of the 3D volumes from the associated step. The scale bar on the displayed micrograph is 30 nm long. For the final step, the viewing direction distribution is presented. In the selected 2D classification output, the 20 highest populated classes are displayed. In the later steps, the inclusion of monomer (M) and dimer (D) in the processing step is shown. B) Correlation between map and model calculated by residue in phenix.validation_cryoem for the two chains of the structure. As the CUB and TE domains are not included in chain B, map-model correlation is only calculated for chain A.

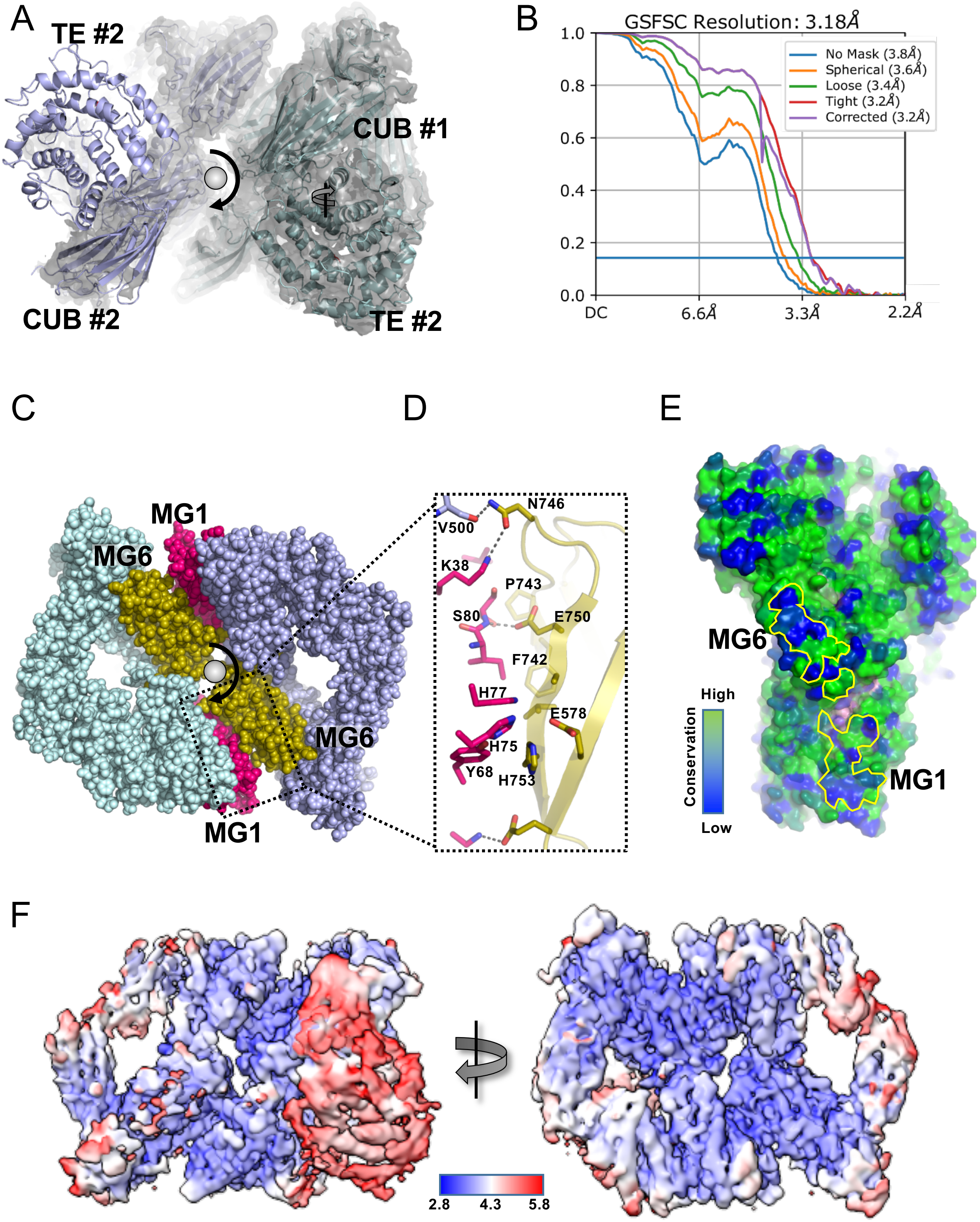
A
TE #2
CUB #1
CUB #2
TE #2
B
GSFSC Resolution: 3.18Å
No Mask (3.8Å)
Spherical (3.6Å)
Loose (3.4Å)
Tight (3.2Å)
Corrected (3.2Å)
1.0
0.8
0.6
0.4
0.2
0.0
DC
6.6Å
3.3Å
2.2Å
C
MG1
MG6
MG6
MG1
D
N746
V500
K38
P743
S80
E750
F742
H77
E578
H75
Y68
H753
E
MG6
High
Conservation
Low
MG1
F
2.8
4.3
5.8

Supplementary Figure 9. Cleaved A2ML1 forms a dimer through a pseudo two-fold symmetric MG1-MG6 interface. A) EM map and full model of the A2ML1-CA dimer. The second pair of CUB and TE domains are placed through superposition since the map quality does not allow experimental modeling. The view is along the central two-fold axis. B) The Fourier shell correlation plot suggests a resolution of 3.2 Å for the A2ML1-CA dimer. C) The dimer interface with MG1 and MG6 domains colored red and yellow, respectively. Compared to panel A, the opposite face of the dimer is displayed. D) Magnified view of one half of the dimer interface. E) Sequence conservation calculated with ConSurf [20] based on the alignment presented in Supplementary Figure 10. The dimer interface is outlined in yellow on one subunit. Residues at the dimer interface appear not to be highly conserved. F) Local resolution of the A2ML1-CA dimer cryo-EM map in two orientations differing by a rotation of 180°. In the left part, the orientation is similar to that in panel A.

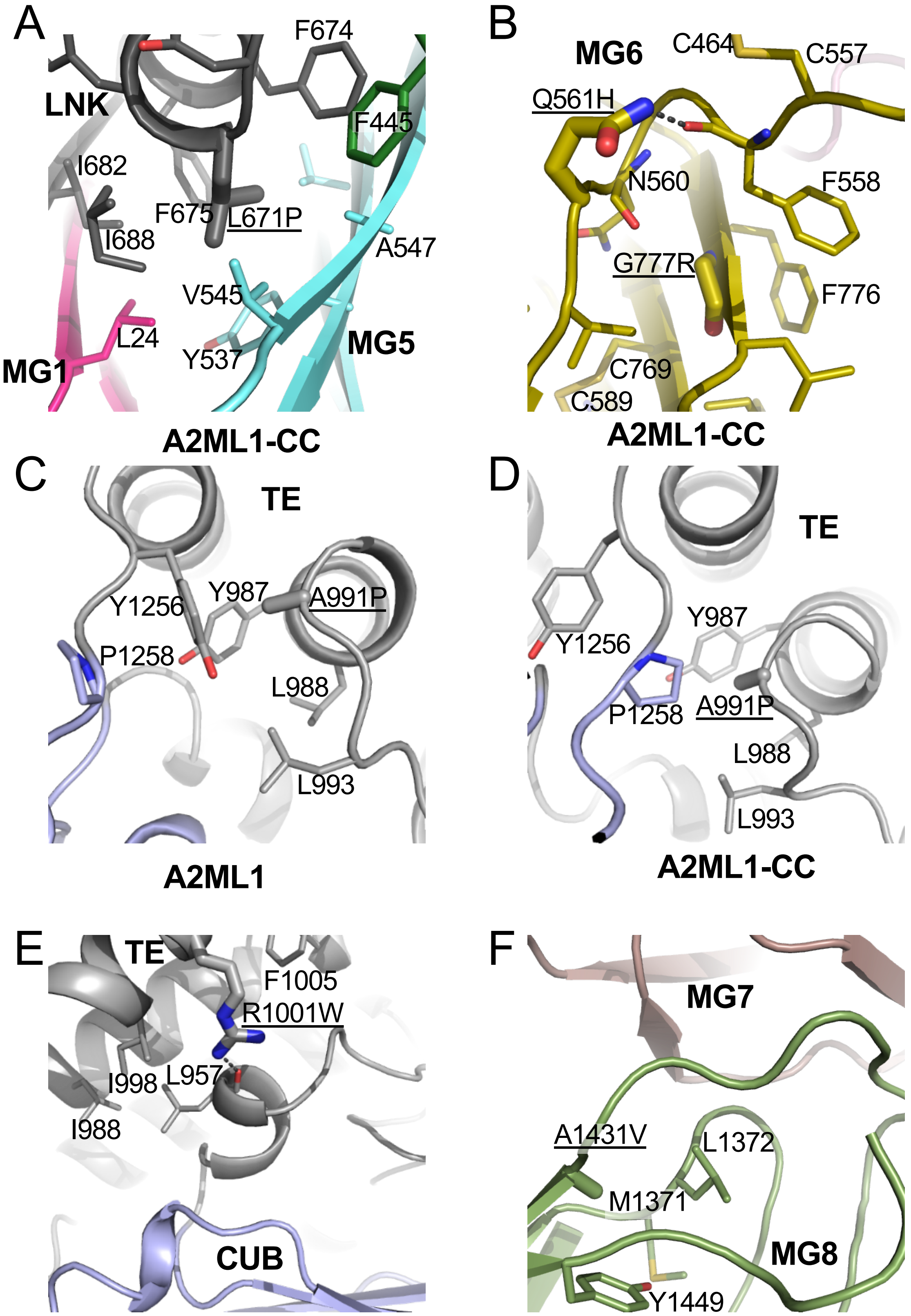
A
LNK
F674
F445
I682
F675
L671P
I688
A547
V545
L24
Y537
MG5
MG1
A2ML1-CC
B
MG6
C464
C557
Q561H
N560
F558
G777R
F776
C769
C589
A2ML1-CC
C
TE
Y1256
Y987
A991P
P1258
L988
L993
A2ML1
D
TE
Y987
Y1256
P1258
A991P
L988
L993
A2ML1-CC
E
TE
F1005
R1001W
I998
L957
I988
CUB
F
MG7
A1431V
L1372
M1371
MG8
Y1449

Supplementary Figure 10. Structural aspects of A2ML1 variants associated with Otitis media. A) Environment around Leu671, which has its side chain buried in nonpolar pocket formed between MG1, MG5 and LNK. Mutation of residue 671 (label underlined in all variants) to proline may weaken LNK interactions with the MG1 and MG5 domains. B) The two residues Gly777 and Gln561 are located close to each other. In the variant G777R, the large arginine side chain is likely to destabilize the MG6 domain fold, and this may alter the interactions between the nearby MG5 domain and the MG6 domain. The effect of the Q561H mutation is likely to be more subtle. C-D) Mutation of Ala991 to proline may have consequences in both native and protease activated A2ML1. A proline substitution will interfere with the following α-helix, but may also exert steric hindrance on Tyr1256 in native A2ML1 and the TE-CUB domain linker in activated A2ML1. E) Mutation of Arg1001 to tryptophan is likely to break the interaction with the small α-helix in the CUB-CE linker. This may destabilize the loop located prior to the Cys970-Gln973 thioester and the TE-CUB domain interface. F) the mutation of Ala1431 to valine is predicted to destabilize the folding of the MG8 domain and may compromise the stability of the nearby MG7-MG8 domain interface.

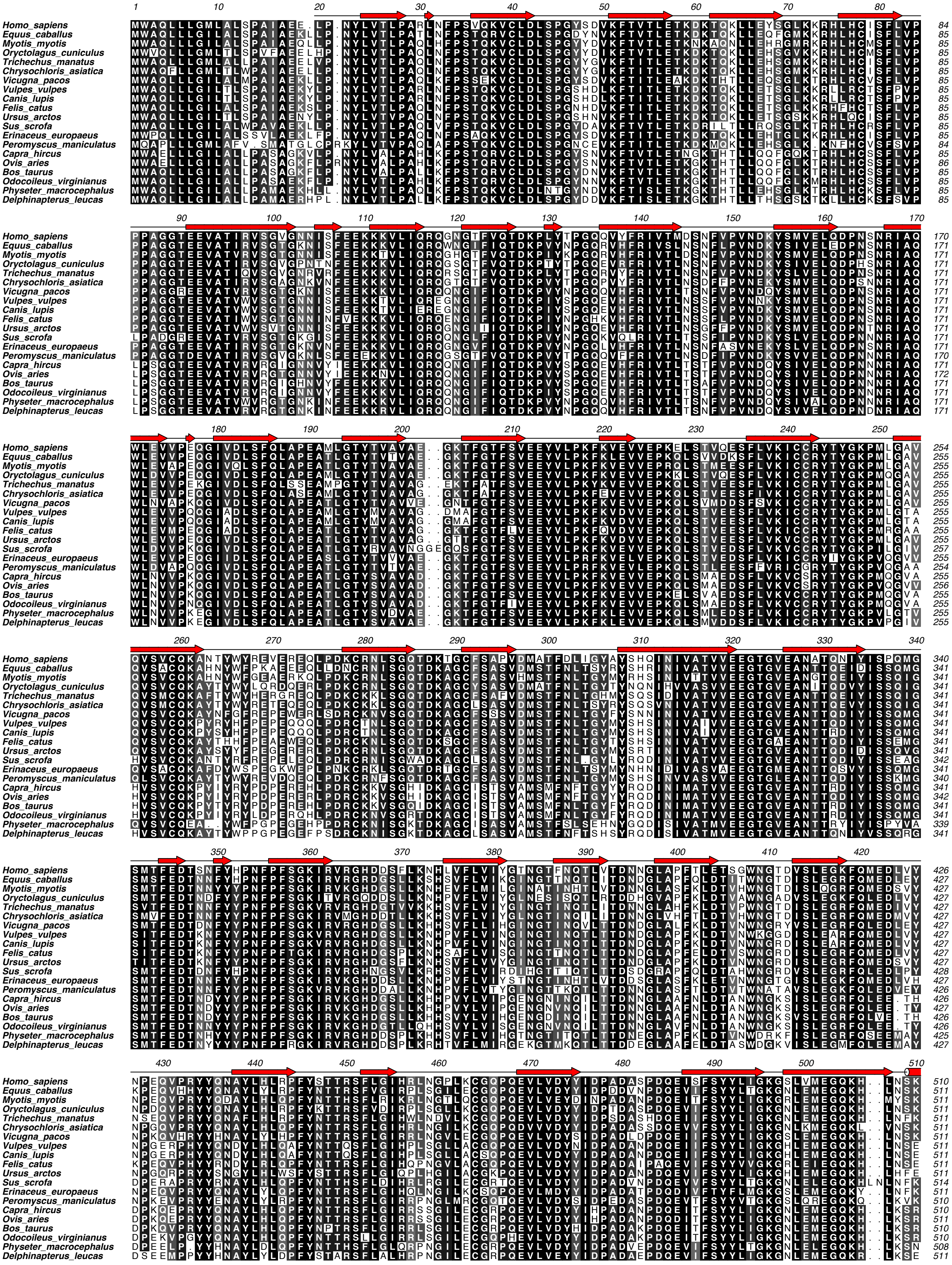
Homo_sapiens
Equus_caballus
Myotis_myotis
Oryctolagus_cuniculus
Trichechus_manatus
Chrysochloris_asiatica
Vicugna_pacos
Vulpes_vulpes
Canis_lupis
Felis_catus
Ursus_arctos
Sus_scrofa
Erinaceus_europaeus
Peromyscus_maniculatus
Capra_hircus
Ovis_aries
Bos_taurus
Odocoileus_virginianus
Physeter_macrocephalus
Delphinapterus_leucas

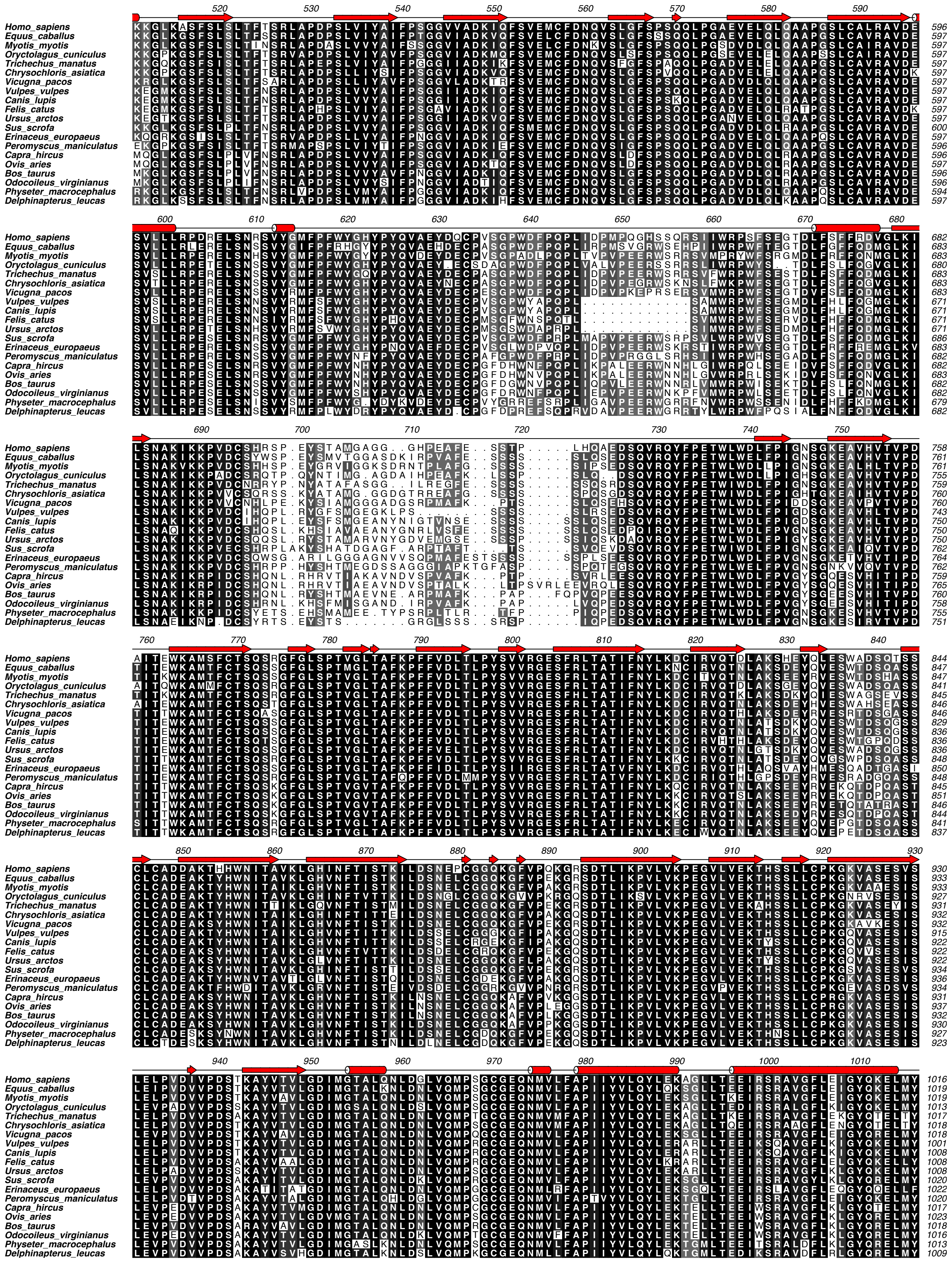
Homo_sapiens
Equus_caballus
Myotis_myotis
Oryctolagus_cuniculus
Trichechus_manatus
Chrysochloris_asiatica
Vicugna_pacos
Vulpes_vulpes
Canis_lupis
Felis_catus
Ursus_arctos
Sus_scrofa
Erinaceus_europaeus
Peromyscus_maniculatus
Capra_hircus
Ovis_aries
Bos_taurus
Odocoileus_virginianus
Physeter_macrocephalus
Delphinapterus_leucas

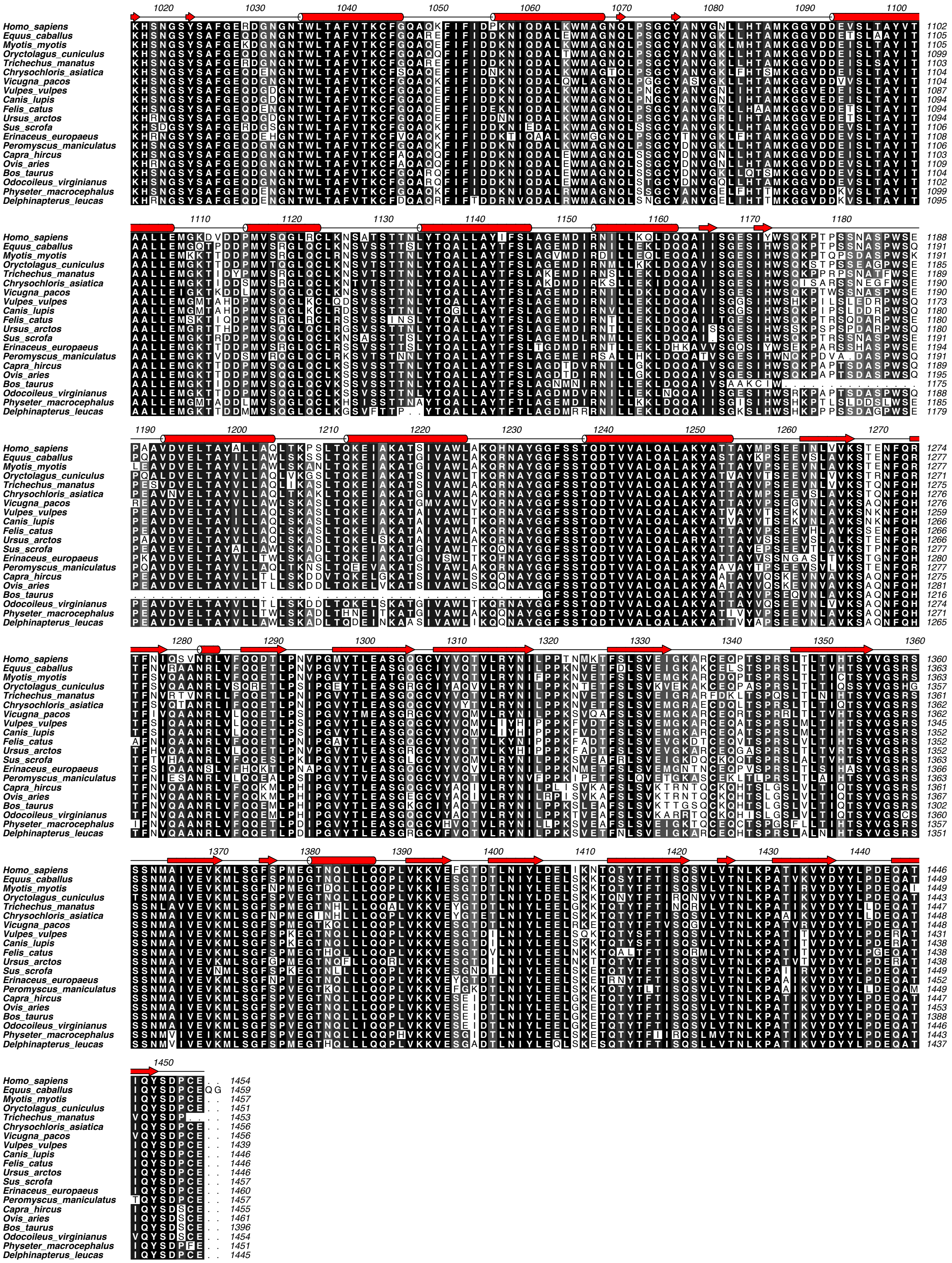
Homo_sapiens
Equus_caballus
Myotis_myotis
Oryctolagus_cuniculus
Trichechus_manatus
Chrysochloris_asiatica
Vicugna_pacos
Vulpes_vulpes
Canis_lupis
Felis_catus
Ursus_arctos
Sus_scrofa
Erinaceus_europaeus
Peromyscus_maniculatus
Capra_hircus
Ovis_aries
Bos_taurus
Odocoileus_virginianus
Physeter_macrocephalus
Delphinapterus_leucas

Supplementary Figure 11. Alignment of representative mammalian A2ML1 sequences. Alignment created with MUSCLE [21] and presented in ALINE [22] of sequences from the UniProt entries for A2ML1 from the listed species. Residue numbers at the top refer to the human A2ML1 sequence. The secondary structure is based on the EM structure of native A2ML1 and calculated by DSSP [23].

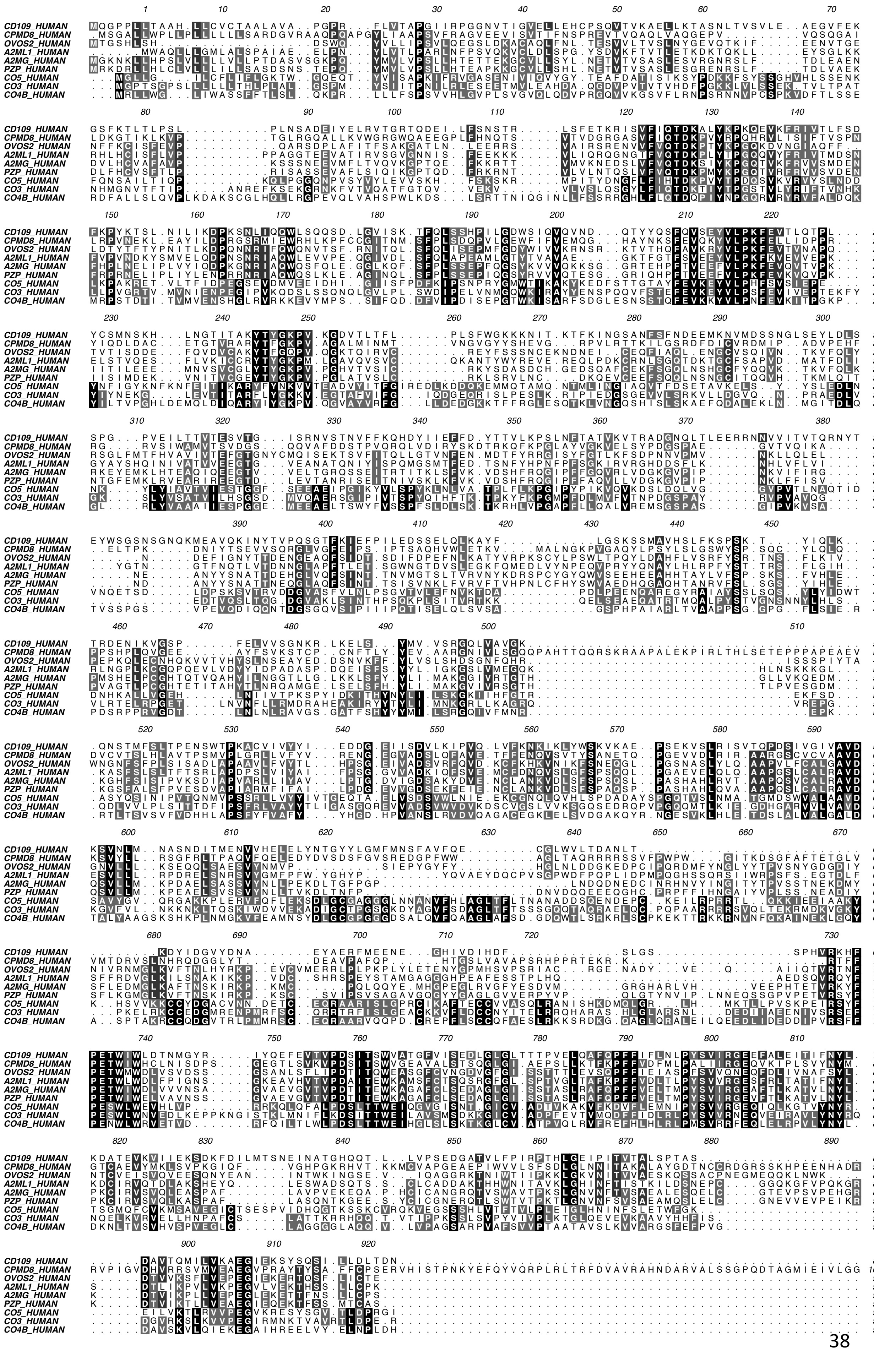

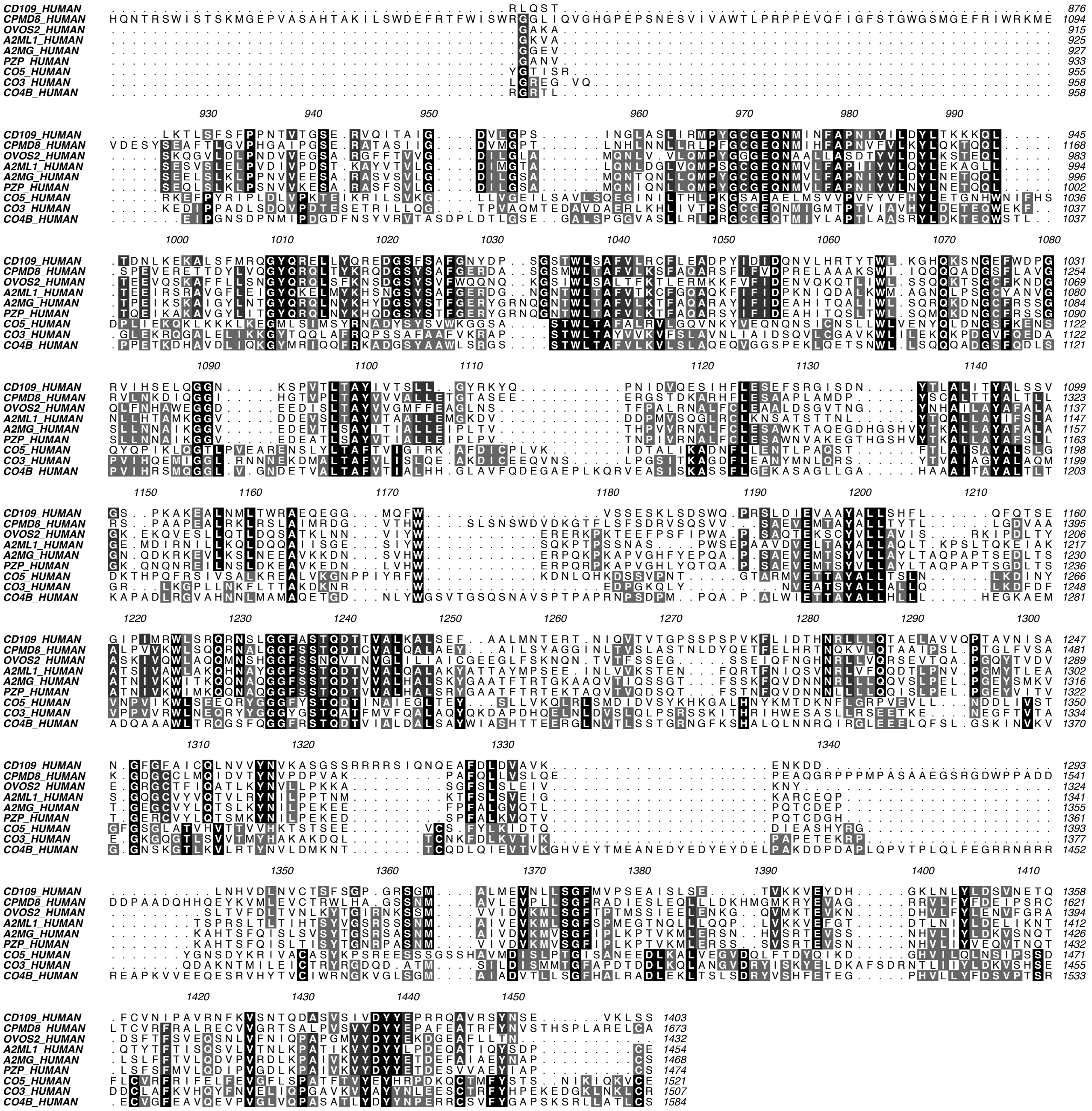

Supplementary Figure 12. Alignment of human members of the A2MF. Alignment created with MUSCLE and presented in ALINE of sequences from the listed UniProt entries. Residue numbers at the top refer to the A2ML1 sequence. CD109_HUMAN; CD109 antigen; CPMD8_HUMAN: C3 and PZP-like alpha-2-macroglobulin domain-containing protein 8; OVOS2_HUMAN: Ovostatin homolog 2; A2ML1_HUMAN: Alpha-2-macroglobulin-like protein 1; A2MG_HUMAN: Alpha-2-macroglobulin; PZP_HUMAN: Pregnancy zone protein; CO5_HUMAN: Complement C5; CO3_HUMAN: Complement C3; CO4B_HUMAN: Complement C4-B.

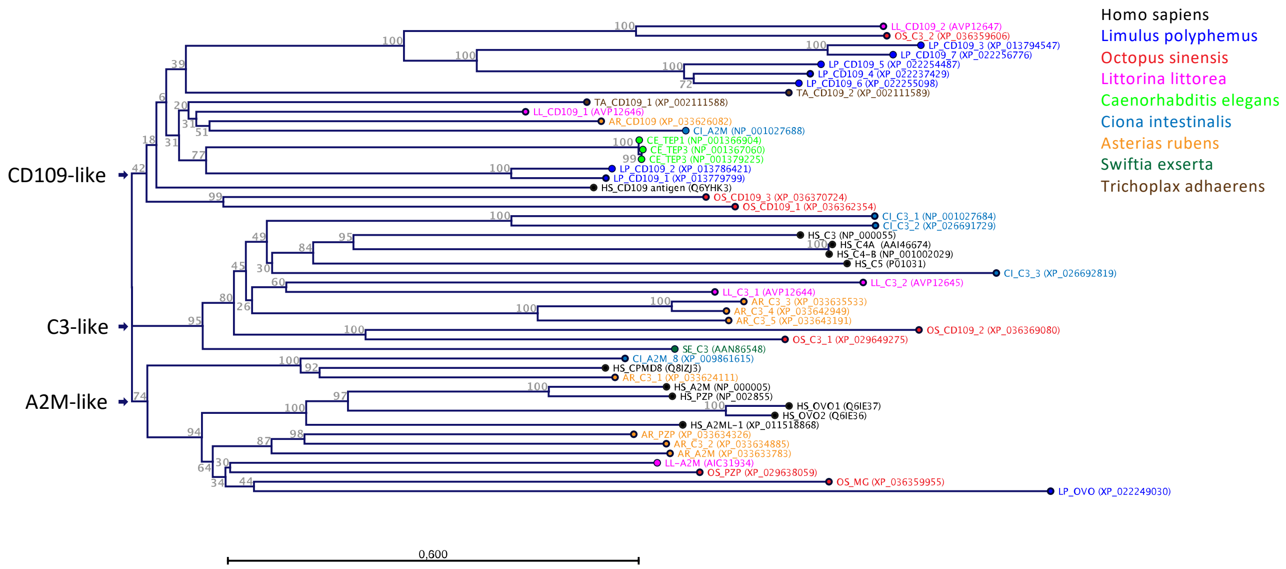

Supplementary Figure 13. A phylogenetic tree of A2MFs from selected representative species of diverse phyla. Human A2MF sequences were retrieved from NCBI and used to search public sequence databases for related sequences from selected species representing diverse phyla. Multiple sequence alignment was created using Clustal-Omega [24] in the CLC Main Workbench, and an unrooted maximum likelihood phylogeny created using default parameter settings and 1000 bootstrap. Branch lengths are given in terms of expected numbers of substitutions per site.

Structure	Native A2ML1	A2ML1-CE monomer	A2ML1-CC monomer	A2ML1-CA dimer
**Data collection**				
Electron microscope	Titan Krios G3i	Titan Krios G3i	Titan Krios G3i	Titan Krios G3i
Camera	Gatan K3	Gatan K3	Gatan K3	Gatan K3
Voltage (kV)	300	300	300	300
Spherical Aberration (mm)	2.7	2.7	2.7	2.7
Pixel size (Å)	0.647	0.647	0.647	0.647
Total Exposure (e/pixel)	60.0	61.1	61.0	60.1
Dose rate (e/pixel/s)	18.2	17.9	18.1	17.9
Defocus min-max (µm)	0.6-1.5	0.6-1.5	0.6-1.5	0.6-1.5
Number of exposure fractions	56	56	56	56
**Image processing**				
Motion correction	cryoSPARC v3	cryoSPARC v3	cryoSPARC v3	cryoSPARC v3
CTF estimate	cryoSPARC v3	cryoSPARC v3	cryoSPARC v3	cryoSPARC v3
Particle selection	cryoSPARC v3	cryoSPARC v3	cryoSPARC v3	cryoSPARC v3
3D classification and refinement	cryoSPARC v3	cryoSPARC v3	cryoSPARC v3	cryoSPARC v3
Micrographs used	7,823	5,845	6,067	5,318
Particles picked	2,104,886	2,429,314	5,605,064	2,394,233
Particles used	653,385	228,619	464,317	174,680
**Refinement**				
PDB entry	7Q5Z	7Q60	7Q61	7Q62
EMDB entry	EMD-13847	EMD-13848	EMD-13849	EMD-13850
Resolution (Å)	3.1	3.2	2.9	3.2
Composition (#)				
Chains	1	1	1	2
Atoms	10493	9455	9538	16193
Protein residues	1344	1212	1210	2045
Bonds (RMSD)				
Length (Å) (# > 4σ)	0.005 (1)	0.007 (1)	0.006 (1)	0.005 (1)
Angles (°) (# > 4σ)	1.008 (16)	1.264 (40)	1.086 (14)	1.122 (27)
MolProbity score	1.72	1.62	1.51	1.59
Clash score	5.22	5.00	3.96	4.86
Ramachandran plot (%)				
Outliers	0.3	0.08	0.25	0.15
Allowed	6.45	5.16	4.50	4.63
Favored	93.25	94.76	95.25	95.22
Ramachandran plot Z-score RMSD				
whole (N = 1335)	-2.00 (0.21)	-2.64 (0.22)	-1.84 (0.22)	-1.74 (0.17)
helix (N = 195)	-2.60 (0.26)	-3.24 (0.24)	-3.00 (0.24)	-2.90 (0.26)
sheet (N = 520)	0.14 (0.23)	-0.26 (0.23)	-0.23 (0.23)	-0.23 (0.17)
loop (N = 620)	-2.21 (0.22)	-2.66 (0.23)	-1.47 (0.24)	-1.65 (0.18)
Rotamer outliers (%)	0.09	0.57	0.1	0.06
Cβ outliers (%)	0.00	0.09	0.00	0.00
Peptide plane (%)				
Cis proline/general	1.4/0.0	1.6/0.0	1.6/0.0	1.8/0.1
Twisted proline/general	0.0/0.1	3.2/0.1	1.6/0.0	0.0/0.1
CaBLAM outliers (%)	4.38	3.44	3.19	2.88
ADP (B-factors)				
Iso/Aniso (#)	10493/0	9455/0	9538/0	16193/0
min/max/mean				
Protein	68.18/388.48/153.99	53.79/309.21/142.31	60.65/236.68/127.22	76.77/365.35/154.37
Ligand			98.41/131.15/111.35	115.22/153.41/133.14
Resolution Estimates (Å)				
d FSC model (0/0.143/0.5)	2.8/2.9/3.9	2.9/3.1/3.7	2.8/2.9/3.3	3.1/3.2/3.6
Model vs. Data				
CC (mask)	0.69	0.74	0.81	0.78
CC (box)	0.80	0.77	0.82	0.80
CC (peaks)	0.64	0.62	0.67	0.65
CC (volume)	0.69	0.74	0.80	0.78

Supplementary Table 1. Statistics for data acquisition, image processingthe and refinement.
Parts of the table were created with phenix.validation_cryoem.

Supplementary Table 2. Data collection and refinement statistics (molecular replacement)

	Native A2ML1#
**Data collection**	
Space group	R 3 2 :H
Cell dimensions	
a, *b*, *c* (Å)	320.15, 320.15, 321.94
α, β, γ (°)	90.00, 90.00, 120.00
Resolution (Å)	49.37- 4.4 (4.557- 4.4)
R_{sym} or R_{merge}	0.2968 (6.112)
$I / \sigma I$	9.46 (0.89)
Completeness (%)	99.71 (99.55)
Redundancy	62.0 (41.8)
**Refinement**	
Resolution (Å)	4.40
No. reflections (work/free)	40159/1846
R_{work} / R_{free}	0.2367/0.2767
No. atoms	
Protein	21458
Ligand/ion	307
Water	0
B-factors	
Protein	342.8
Ligand/ion	342.4
Water	
R.m.s. deviations	
Bond lengths (Å)	0.007
Bond angles (°)	0.89

#Four data sets collected from three crystals were merged. *Values in parentheses are for highest-resolution shell.

Supplementary Table 3. The glycosylation pattern of recombinant A2ML1.

Glycosylation sites predicted by GPMAW	Listed on UniProt	Site identified with MS without glycosylation	Site identified with MS with glycosylation	Glycans identified
104 (NIS)		x	x	HexNAc(5)Hex(4)Fuc(2)
120 (NGT)	X	X	x	HexNAc(2)Hex(7)<br>HexNAc(2)Hex(8)<br>HexNAc(2)Hex(9)<br>HexNAc(2)Hex(6)<br>HexNAc(2)Hex(7)Fuc(1)<br>HexNAc(2)Hex(6)Fuc(1)<br>HexNAc(2)Hex(4)Fuc(1)
281 (NLS)	X		X	HexNAc(4)Hex(5)Fuc(1)NeuAc(1)<br>HexNAc(5)Hex(6)Fuc(1)NeuAc(1)<br>HexNAc(3)Hex(6)

				HexNAc(2)Hex(6)<br>HexNAc(4)Hex(5)Fuc(1)<br>HexNAc(4)Hex(4)Fuc(1)<br>HexNAc(5)Hex(5)Fuc(1)NeuAc(1)
328 (NAT)				
384 (NGT)				
388 (NQT)				
409 (NGT)	X		X	HexNAc(2)Hex(5)<br>HexNAc(5)Hex(3)Fuc(1)<br>HexNAc(5)Hex(4)Fuc(1)<br>HexNAc(4)Hex(5)Fuc(1)NeuAc(1)<br>HexNAc(5)Hex(6)Fuc(2)<br>HexNAc(6)Hex(5)Fuc(2)<br>HexNAc(5)Hex(6)Fuc(1)NeuAc(1)<br>HexNAc(4)Hex(4)Fuc(1)<br>HexNAc(5)Hex(3)Fuc(1)

609 (NRS)		x	x	HexNAc(2)Hex(7)<br>HexNAc(2)Hex(8)<br>HexNAc(2)Hex(9)<br>HexNAc(2)Hex(10)<br>HexNAc(2)Hex(6)<br>HexNAc(6)Hex(6)Fuc(1)NeuAc(3)
857 (NIT)	X		x	HexNAc(2)Hex(2)<br>HexNAc(2)Hex(2)Fuc(1)<br>HexNAc(2)Hex(5)<br>HexNAc(2)Hex(7)<br>HexNAc(2)Hex(7)Fuc(1)<br>HexNAc(2)Hex(8)<br>HexNAc(3)Hex(3)<br>HexNAc(3)Hex(3)Fuc(1)

				HexNAc(3)Hex(4)<br>HexNAc(3)Hex(4)Fuc(1)<br>HexNAc(3)Hex(4)Fuc(1)NeuAc(1)<br>HexNAc(3)Hex(4)Fuc(2)NeuAc(1)<br>HexNAc(3)Hex(5)<br>HexNAc(4)Hex(3)<br>HexNAc(4)Hex(3)Fuc(1)<br>HexNAc(4)Hex(4)<br>HexNAc(4)Hex(4)Fuc(1)<br>HexNAc(4)Hex(4)NeuAc(1)<br>HexNAc(4)Hex(5)<br>HexNAc(4)Hex(5)Fuc(1)<br>HexNAc(4)Hex(5)Fuc(1)NeuAc(1)

				HexNAc(4)Hex(5)Fuc(1)NeuAc(2)<br>HexNAc(4)Hex(5)NeuAc(1)<br>HexNAc(4)Hex(5)NeuAc(2)<br>HexNAc(4)Hex(5)Fuc(2)NeuAc(1)<br>HexNAc(4)Hex(6)Fuc(1)<br>HexNAc(5)Hex(3)<br>HexNAc(5)Hex(3)Fuc(1)<br>HexNAc(5)Hex(4)<br>HexNAc(5)Hex(4)Fuc(1)<br>HexNAc(5)Hex(4)Fuc(2)<br>HexNAc(5)Hex(5)<br>HexNAc(5)Hex(5)Fuc(1)<br>HexNAc(5)Hex(5)Fuc(1)NeuAc(1)

				HexNAc(5)Hex(5)Fuc(1)NeuAc(2)<br>HexNAc(5)Hex(5)Fuc(2)<br>HexNAc(5)Hex(5)Fuc(2)NeuAc(1)<br>HexNAc(5)Hex(5)NeuAc(1)<br>HexNAc(5)Hex(5)NeuAc(2)<br>HexNAc(5)Hex(6)<br>HexNAc(5)Hex(6)Fuc(1)<br>HexNAc(5)Hex(6)Fuc(1)NeuAc(1)<br>HexNAc(5)Hex(6)Fuc(1)NeuAc(3)<br>HexNAc(5)Hex(6)NeuAc(1)<br>HexNAc(5)Hex(6)NeuAc(3)<br>HexNAc(6)Hex(6)Fuc(2)NeuAc(1)<br>HexNAc(6)Hex(6)NeuAc(2)

				HexNAc(6)Hex(6)NeuAc(3)<br>HexNAc(6)Hex(7)Fuc(1)NeuAc(4)<br>HexNAc(6)Hex(7)NeuAc(4)
867 (NFT)		x	X	HexNAc(4)Hex(5)NeuAc(1)<br>HexNAc(3)Hex(3)<br>HexNAc(2)Hex(5)<br>HexNAc(3)Hex(4)<br>HexNAc(4)Hex(3)<br>HexNAc(2)Hex(6)<br>HexNAc(3)Hex(5)<br>HexNAc(4)Hex(4)<br>HexNAc(3)Hex(4)NeuAc(1)<br>HexNAc(3)Hex(6)<br>HexNAc(3)Hex(6)NeuAc(1)

				HexNAc(4)Hex(4)Fuc(1)<br>HexNAc(4)Hex(5)<br>HexNAc(5)Hex(3)Fuc(1)<br>HexNAc(5)Hex(4)<br>HexNAc(3)Hex(5)NeuAc(1)<br>HexNAc(5)Hex(4)Fuc(1)<br>HexNAc(5)Hex(5)<br>HexNAc(2)Hex(9)<br>HexNac(2)Hex(8)<br>HexNAc(3)Hex(6)NeuAc(1)<br>HexNAc(4)Hex(5)Fuc(1)NeuAc(1)<br>HexNAc(5)Hex(5)Fuc(1)NeuAc(1)<br>HexNAc(2)Hex(7)<br>HexNAc(3)Hex(4)NeuAc(1)

				HexNAc(4)Hex(4)Fuc(1)<br>HexNAc(4)Hex(5)Fuc(1)<br>HexNAc(4)Hex(4)NeuAc(1)<br>HexNAc(5)Hex(6)Fuc(1)
1020 (NGS)	X		X	HexNAc(4)Hex(5)Fuc(1)NeuAc(1)<br>HexNAc(2)Hex(5)<br>HexNAc(2)Hex(6)<br>HexNAc(6)Hex(4)<br>HexNAc(2)Hex(6)Phospho(1)<br>HexNAc(2)Hex(9)<br>HexNAc(3)Hex(3)<br>HexNAc(3)Hex(6)NeuAc(1)<br>HexNAc(4)Hex(4)NeuAc(1)<br>HexNAc(4)Hex(4)Fuc(1)

				HexNAc(4)Hex(3)Fuc(1)<br>HexNAc(5)Hex(3)Fuc(1)<br>HexNAc(5)Hex(3)<br>HexNAc(5)Hex(4)<br>HexNAc(5)Hex(4)Fuc(1)<br>HexNAc(5)Hex(4)Fuc(2)<br>HexNAc(4)Hex(5)Fuc(1)<br>HexNAc(4)Hex(5)<br>HexNAc(4)Hex(5)NeuAc(1)<br>HexNAc(4)Hex(5)NeuAc(2)<br>HexNAc(5)Hex(5)Fuc(1)<br>HexNAc(5)Hex(5)NeuAc(1)<br>HexNAc(5)Hex(5)NeuAc(2)<br>HexNAc(5)Hex(5)Fuc(2)<br>HexNAc(5)Hex(6)Fuc(1)

				HexNAc(5)Hex(6)Fuc(1)NeuAc(1)<br>HexNAc(5)Hex(6)Fuc(1)NeuAc(2)<br>HexNAc(5)Hex(6)Fuc(1)NeuAc(3)<br>HexNAc(5)Hex(6)Fuc(2)<br>HexNAc(5)Hex(6)Fuc(2)NeuAc(1)<br>HexNAc(5)Hex(6)Fuc(3)<br>HexNAc(5)Hex(6)NeuAc(3)<br>HexNAc(6)Hex(7)Fuc(1)NeuAc(2)<br>HexNAc(6)Hex(7)Fuc(3)<br>HexNAc(4)Hex(5)Fuc(2)NeuAc(1)<br>HexNAc(4)Hex(5)Fuc(3)NeuAc(1)<br>HexNAc(5)Hex(5)Fuc(1)NeuAc(1)<br>HexNAc(5)Hex(5)Fuc(1)NeuAc(2)<br>HexNAc(5)Hex(5)Fuc(2)NeuAc(1)

				HexNAc(5)Hex(6)Fuc(2)<br><br>HexNAc(4)Hex(5)Fuc(1)NeuAc(2)<br><br>HexNAc(4)Hex(5)Fuc(1)NeuAc(1)<br><br>HexNAc(5)Hex(6)Fuc(3)
1182 (NAS)		x		

A2ML1 Mutation	Domain	References	Conservation (%)	Possible effect on structure
p.Thr55Ile	MG1	9	**High**	**Surface exposed, no obvious impact on structure**
p.Val296Ala	MG3	8	**100**	**Buried residue in β-sheet, likely to destabilize MG3 domain and interfere with MG function in activated A2ML1.**
p.Pro356Arg	MG4	8	**100**	**Possibly interferes with backbone conformation and thereby influences the MG4-MG5 packing.**
p.Gln436His	MG4	9	**82**	**Surface exposed close to putative protease interacting loop in MG4.**
p.Gln561His	MG6	9	**95**	**The glutamine forms a hydrogen bond with Phe558 in the MG5-MG6 linker. Substitution may have subtle conformational effects. Close to Gly777**
p.Leu671Pro	LNK	9	**91**	**Residue in LNK domain α-helix.<br>The lecuine side chain is buried in nonpolar pocket formed between MG1, MG5 and LNK. Mutation to proline likely to weaken LNK-interactions with MG1 and MG5**
p.Arg730His	BR-C	9	**100**	**First residue in hydrophobic plug that passes through interdomain channel. The arginine side chain is tightly recognized in activated A2ML1 as part of BRC. A histidine cannot substitute for the arginine**
p.Phe733Leu	BR-C	9	**100**	**See above for Arg730. Interferes with hydrophobic plug in A2ML1 and baitC recognition in activated A2ML1.**
p.Pro743Leu	MG6	9	**100**	**Surface exposed. Mutation may chain main chain conformation and interfere with the MG6-MG2 domain interface.**
p.Gly777Arg	MG6	9	**100**	**The large arginine side chain introduced cannot be accommodated, this is likely to interfere with MG6 folding and MG5-MG6 interactions. Close to Gln561.**
p.Ala810Thr	MG7	8	**100**	**Alanine side chain is buried in non-polar environment, the substitution to threonine will destabilize the MG7 domain**
p.Asp849Tyr	MG7	9	**95**	**Surface exposed residue. The large tyrosine side chain possibly interfere with MG3-MG7 interaction in activated A2ML1.**
p.Ala991Pro	TE	9	**55**	**Proline will destabilize α-helix in TE domain. The larger proline side chain is likely to affect the CUB-TE domain interface in both native and activated A2ML1.**
p.Arg1001Trp	TE	8	**86**	**Arginine interacts with a small a-helix in the CUB-TE domain interface. Mutation to tryptophan may destabilize this interaction and thereby destabilize the nearby Cys970-Gln973 thioester in native A2ML1.**
p.Ala1164Val	TE	9	**100**	**Alanine faces nonpolar pocket. Substitution with valine may destabilize the Ala1164-Ser1174. In native A2ML1, this may weaken TE-CUB interactions, whereas in activated A2ML1, residues after 1174 faces the putative protease binding pocket**
p.Ala1431Val	MG8	8	**100**	**The alanine is buried in a nonpolar environment. The larger valine is likely to destabilize the MG8 domain and interfere with MG7-MG8 domain interactions in native A2ML1. In activated A2ML1, the substitution could have impact on a possible MG8-receptor interaction.**

Supplementary table 4. Mutations in A2ML1 in individuals susceptible to otitis media. The conservation was calculated with the ConSurf server based on the alignment of mammalian A2ML1 sequences in Suppl Figure 11.

Supplemantry References

1 Galliano, M. F. *et al.* A novel protease inhibitor of the alpha2-macroglobulin family expressed in the human epidermis. *J Biol Chem* **281**, 5780-5789, doi:10.1074/jbc.M508017200 (2006).

2 Enghild, J. J., Salvesen, G., Thogersen, I. B. & Pizzo, S. V. Proteinase binding and inhibition by the monomeric alpha-macroglobulin rat alpha 1-inhibitor-3. *J Biol Chem* **264**, 11428-11435 (1989).

3 Sayers, C. A. & Barrett, A. J. Binding of anhydrotrypsin to alpha 2-macroglobulin. *Biochem J* **189**, 255-261, doi:10.1042/bj1890255 (1980).

4 Arandjelovic, S., Van Sant, C. L. & Gonias, S. L. Limited mutations in full-length tetrameric human alpha2-macroglobulin abrogate binding of platelet-derived growth factor-BB and transforming growth factor-beta1. *J Biol Chem* **281**, 17061-17068, doi:10.1074/jbc.M602217200 (2006).

5 Marrero, A. *et al.* The crystal structure of human alpha2-macroglobulin reveals a unique molecular cage. *Angew Chem Int Ed Engl* **51**, 3340-3344, doi:10.1002/anie.201108015 (2012).

6 Rooijakkers, S. H. *et al.* Structural and functional implications of the alternative complement pathway C3 convertase stabilized by a staphylococcal inhibitor. *Nat Immunol* **10**, 721-727, doi:10.1038/ni.1756 (2009).

7 Giese, A. P. J. *et al.* Genomics of Otitis Media (OM): Molecular Genetics Approaches to Characterize Disease Pathophysiology. *Front Genet* **11**, 313, doi:10.3389/fgene.2020.00313 (2020).

8 Santos-Cortez, R. L. *et al.* Rare A2ML1 variants confer susceptibility to otitis media. *Nat Genet* **47**, 917-920, doi:10.1038/ng.3347 (2015).

9 Larson, E. D. *et al.* A2ML1 and otitis media: novel variants, differential expression, and relevant pathways. *Hum Mutat* **40**, 1156-1171, doi:10.1002/humu.23769 (2019).

10 Wong, S. G. & Dessen, A. Structure of a bacterial alpha2-macroglobulin reveals mimicry of eukaryotic innate immunity. *Nat Commun* **5**, 4917, doi:10.1038/ncomms5917 (2014).

11 Santamaria, M. E., Diaz-Mendoza, M., Diaz, I. & Martinez, M. Plant protein peptidase inhibitors: an evolutionary overview based on comparative genomics. *BMC Genomics* **15**, 812, doi:10.1186/1471-2164-15-812 (2014).

12 Enghild, J. J. *et al.* Alpha-macroglobulin from Limulus polyphemus exhibits proteinase inhibitory activity and participates in a hemolytic system. *Biochemistry* **29**, 10070-10080, doi:10.1021/bi00495a009 (1990).

13 Thøgersen, I. B., Salvesen, G., Brucato, F. H., Pizzo, S. V. & Enghild, J. J. Purification and characterization of an alpha-macroglobulin proteinase inhibitor from the mollusc Octopus vulgaris. *Biochem J* **285 (Pt 2)**, 521-527, doi:10.1042/bj2850521 (1992).

14 Fujito, N. T., Sugimoto, S. & Nonaka, M. Evolution of thioester-containing proteins revealed by cloning and characterization of their genes from a cnidarian sea anemone, Haliplanella lineate. *Dev Comp Immunol* **34**, 775-784, doi:10.1016/j.dci.2010.02.011 (2010).

15 Prosper, J. Y. A. *Characterization of CD109* ph.D. thesis, University of Toronto, (2011).

16 Srivastava, M. *et al.* The Trichoplax genome and the nature of placozoans. *Nature* **454**, 955-960, doi:10.1038/nature07191 (2008).

17 Lin, M. *et al.* Cell surface antigen CD109 is a novel member of the alpha(2) macroglobulin/C3, C4, C5 family of thioester-containing proteins. *Blood* **99**, 1683-1691, doi:10.1182/blood.v99.5.1683 (2002).

18 Finnson, K. W. *et al.* Identification of CD109 as part of the TGF-beta receptor system in human keratinocytes. *FASEB J* **20**, 1525-1527, doi:10.1096/fj.05-5229fje (2006).

19 Bizet, A. A. *et al.* The TGF-beta co-receptor, CD109, promotes internalization and degradation of TGF-beta receptors. *Biochim Biophys Acta* **1813**, 742-753, doi:10.1016/j.bbamcr.2011.01.028 (2011).

20 Ashkenazy, H. *et al.* ConSurf 2016: an improved methodology to estimate and visualize evolutionary conservation in macromolecules. *Nucleic Acids Res* **44**, W344-350, doi:10.1093/nar/gkw408 (2016).

21 Edgar, R. C. MUSCLE: multiple sequence alignment with high accuracy and high throughput. *Nucleic Acids Res* **32**, 1792-1797, doi:10.1093/nar/gkh340 (2004).

22 Bond, C. S. & Schuttelkopf, A. W. ALINE: a WYSIWYG protein-sequence alignment editor for publication-quality alignments. *Acta Crystallogr D Biol Crystallogr* **65**, 510-512, doi:10.1107/S0907444909007835 (2009).
23 Kabsch, W. & Sander, C. Dictionary of protein secondary structure: pattern recognition of hydrogen-bonded and geometrical features. *Biopolymers* **22**, 2577-2637, doi:10.1002/bip.360221211 (1983).
24 Sievers, F. *et al.* Fast, scalable generation of high-quality protein multiple sequence alignments using Clustal Omega. *Mol Syst Biol* **7**, 539, doi:10.1038/msb.2011.75 (2011).
