# Supplementary material for: Cryo-EM structures of human A2ML1 elucidate the protease-inhibitory mechanism of the A2M family
Source: Nat Commun. 2022 May 31;13:3033. doi: 10.1038/s41467-022-30758-x (PMC9156758; doi:10.1038/s41467-022-30758-x)
Supplement: Supplementary file 2 — Reporting Summary [file 41467_2022_30758_MOESM2_ESM.pdf]

nature portfolio

Corresponding author(s): Gregers R. Andersen and Jan J. Enghild

Last updated by author(s): 04/03/2022

Reporting Summary

Nature Portfolio wishes to improve the reproducibility of the work that we publish. This form provides structure for consistency and transparency in reporting. For further information on Nature Portfolio policies, see our Editorial Policies and the Editorial Policy Checklist.

Statistics

For all statistical analyses, confirm that the following items are present in the figure legend, table legend, main text, or Methods section.

n/a	Confirmed	
☐	☒	The exact sample size (n) for each experimental group/condition, given as a discrete number and unit of measurement
☐	☒	A statement on whether measurements were taken from distinct samples or whether the same sample was measured repeatedly
☒	☐	The statistical test(s) used AND whether they are one- or two-sided<br>*Only common tests should be described solely by name; describe more complex techniques in the Methods section.*
☒	☐	A description of all covariates tested
☒	☐	A description of any assumptions or corrections, such as tests of normality and adjustment for multiple comparisons
☐	☒	A full description of the statistical parameters including central tendency (e.g. means) or other basic estimates (e.g. regression coefficient) AND variation (e.g. standard deviation) or associated estimates of uncertainty (e.g. confidence intervals)
☐	☒	For null hypothesis testing, the test statistic (e.g. F, t, r) with confidence intervals, effect sizes, degrees of freedom and P value noted<br>*Give P values as exact values whenever suitable.*
☒	☐	For Bayesian analysis, information on the choice of priors and Markov chain Monte Carlo settings
☒	☐	For hierarchical and complex designs, identification of the appropriate level for tests and full reporting of outcomes
☒	☐	Estimates of effect sizes (e.g. Cohen's d, Pearson's r), indicating how they were calculated

Our web collection on statistics for biologists contains articles on many of the points above.

Software and code

Policy information about availability of computer code

Data collection	Xray data collection was with open source MXCUBE3 (version 3) software at BioMAX . Data collection of EM data on the Titan Krios microscope was performed with the EPU 2.7 software from Thermo Fisher.
Data analysis	Xray data was reduced with XDS built 20180808 followed by structure determination with various programs in the phenix 1.19.2-4158 package. EM data was analyzed with cryosparc v3 and structures refined in phenix 1.19.2-4158 . These software packages are free to academic users. Mass spectrometry data were analyzed using the Mascot search engine (version 2.5)and Byonic search engine (Version 3.7.13. Protein Metrics).

For manuscripts utilizing custom algorithms or software that are central to the research but not yet described in published literature, software must be made available to editors and reviewers. We strongly encourage code deposition in a community repository (e.g. GitHub). See the Nature Portfolio guidelines for submitting code & software for further information.

Data

Policy information about availability of data

All manuscripts must include a data availability statement. This statement should provide the following information, where applicable:

- Accession codes, unique identifiers, or web links for publicly available datasets
- A description of any restrictions on data availability
- For clinical datasets or third party data, please ensure that the statement adheres to our policy

The mass spectrometry data have been deposited to the ProteomeXchange Consortium via the PRIDE partner repository 59 with the dataset identifier PXD032010. Atomic structures for the EM and X-ray structures of native A2ML1, A2ML1-CC, A2ML1-CE, and the A2ML1-CA dimer are deposited at the Protein Data Bank under the entries presented in supplementary table 1 and supplementary table 2. The cryo-EM maps corresponding to these EM-derived structures are deposited at the

EMDB under the entries presented in supplementary table 1.

Field-specific reporting

Please select the one below that is the best fit for your research. If you are not sure, read the appropriate sections before making your selection.

- [x] Life sciences
- [ ] Behavioural & social sciences
- [ ] Ecological, evolutionary & environmental sciences

For a reference copy of the document with all sections, see nature.com/documents/nr-reporting-summary-flat.pdf

Life sciences study design

All studies must disclose on these points even when the disclosure is negative.

Sample size	The number of micrographs used for each EM structure are listed in suppl table 1.
Data exclusions	in xray data, reflections beyond 4.4 Å were excluded. In EM data processing, low quality 2D and 3D classes were excluded manually based on resolution and visual inspection. Outlier micrographs were discarded with Curate Exposures in cryosparc.
Replication	X-ray data were collected from 3 different crystal in 4 wedges. Cryo-EM data were collected once for each sample.
Randomization	No structural biology experiment in this study involved randomization of samples.
Blinding	No structural biology experiment in this study involved blinding.

Reporting for specific materials, systems and methods

We require information from authors about some types of materials, experimental systems and methods used in many studies. Here, indicate whether each material, system or method listed is relevant to your study. If you are not sure if a list item applies to your research, read the appropriate section before selecting a response.

Materials & experimental systems

n/a	Involved in the study	
x		Antibodies
	x	Eukaryotic cell lines
x		Palaeontology and archaeology
x		Animals and other organisms
	x	Human research participants
x		Clinical data
x		Dual use research of concern

Methods

n/a	Involved in the study	
x		ChIP-seq
x		Flow cytometry
x		MRI-based neuroimaging

Eukaryotic cell lines

Policy information about cell lines

Cell line source(s)	HEK 293-F cells were purchased from Thermo Fisher Scientific (catalogue number R79007).
Authentication	The 293-F cells were exclusively used for protein expression and no authentication analysis was not performed.
Mycoplasma contamination	All cell lines tested negative for mycoplasma contamination.
Commonly misidentified lines (See ICLAC register)	The cell line used in this study in not commonly misidentified.

Human research participants

Policy information about studies involving human research participants

Population characteristics	One adult was included in this study.
Recruitment	No patients were specifically recruited for the study.
Ethics oversight	A deidentified normal human skin sample was collected at Aarhus University Hospital, with full informed consent, from an adult undergoing plastic surgery. The procedure was approved by the local ethical committee, Region Midtjylland, Denmark (M-20110027) and carried out according to the Declaration of Helsinki Principles.

Note that full information on the approval of the study protocol must also be provided in the manuscript.
